# Supplementary material for: Low-inflammatory lipid nanoparticle-based mRNA vaccine elicits protective immunity against H5N1 influenza virus with reduced adverse reactions
Source: Mol Ther. 2024 Dec 17;33(2):529–47. doi: 10.1016/j.ymthe.2024.12.032 (PMC11852987; doi:10.1016/j.ymthe.2024.12.032)
Supplement: Document S1. Figures S1–S21 and Tables S1 and S2 [file mmc1.pdf]

YMTHE, Volume *33*

Supplemental Information

Low-inflammatory lipid nanoparticle-based mRNA vaccine elicits protective immunity against H5N1 influenza virus with reduced adverse reactions

Atsushi Kawai, Taro Shimizu, Hiroki Tanaka, Shintaro Shichinohe, Jessica Anindita, Mika Hirose, Eigo Kawahara, Kota Senpuku, Makoto Shimooka, Le Thi Quynh Mai, Ryo Suzuki, Takuto Nogimori, Takuya Yamamoto, Toshiro Hirai, Takayuki Kato, Tokiko Watanabe, Hidetaka Akita, and Yasuo Yoshioka

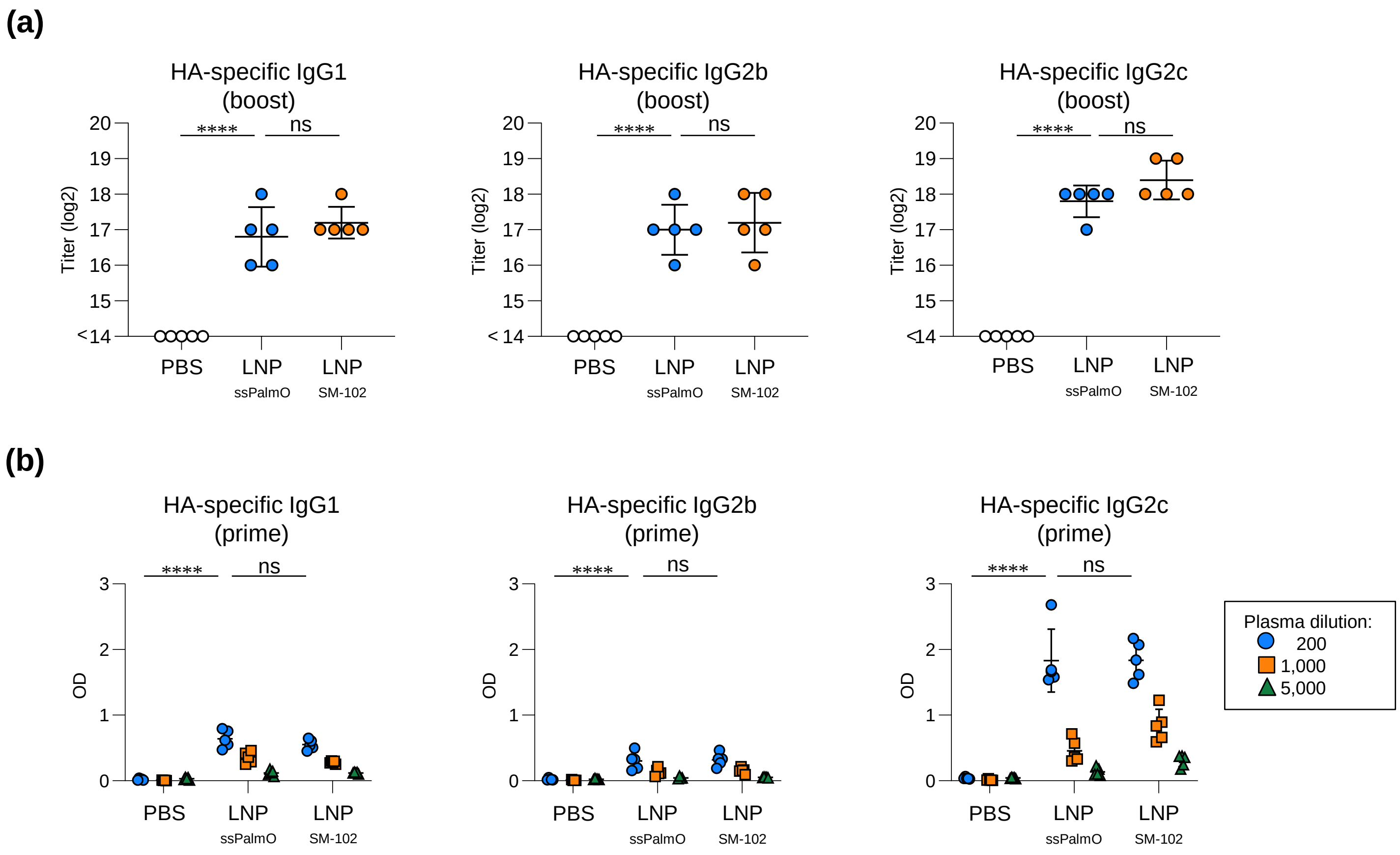

Figure S1. Antibody responses against HA following subcutaneous immunization with HA-LNP. Mice were subcutaneously immunized with HA-LNP on days 0 (prime) and 21 (boost). (a) The titers of HA-specific IgG1, IgG2b, and IgG2c in the plasma on day 35 were evaluated using ELISA. These data are related to Figure 2a. (b) Plasma levels of HA-specific IgG1, IgG2b, and IgG2c on day 14 were evaluated using ELISA. (a, b) n=5 per group. Data are means ± SD. $^{****}P < 0.0001$, Tukey's multiple-comparisons test. ns, not statistically significant. (b) Tukey's multiple comparison test performed at a dilution of 200.

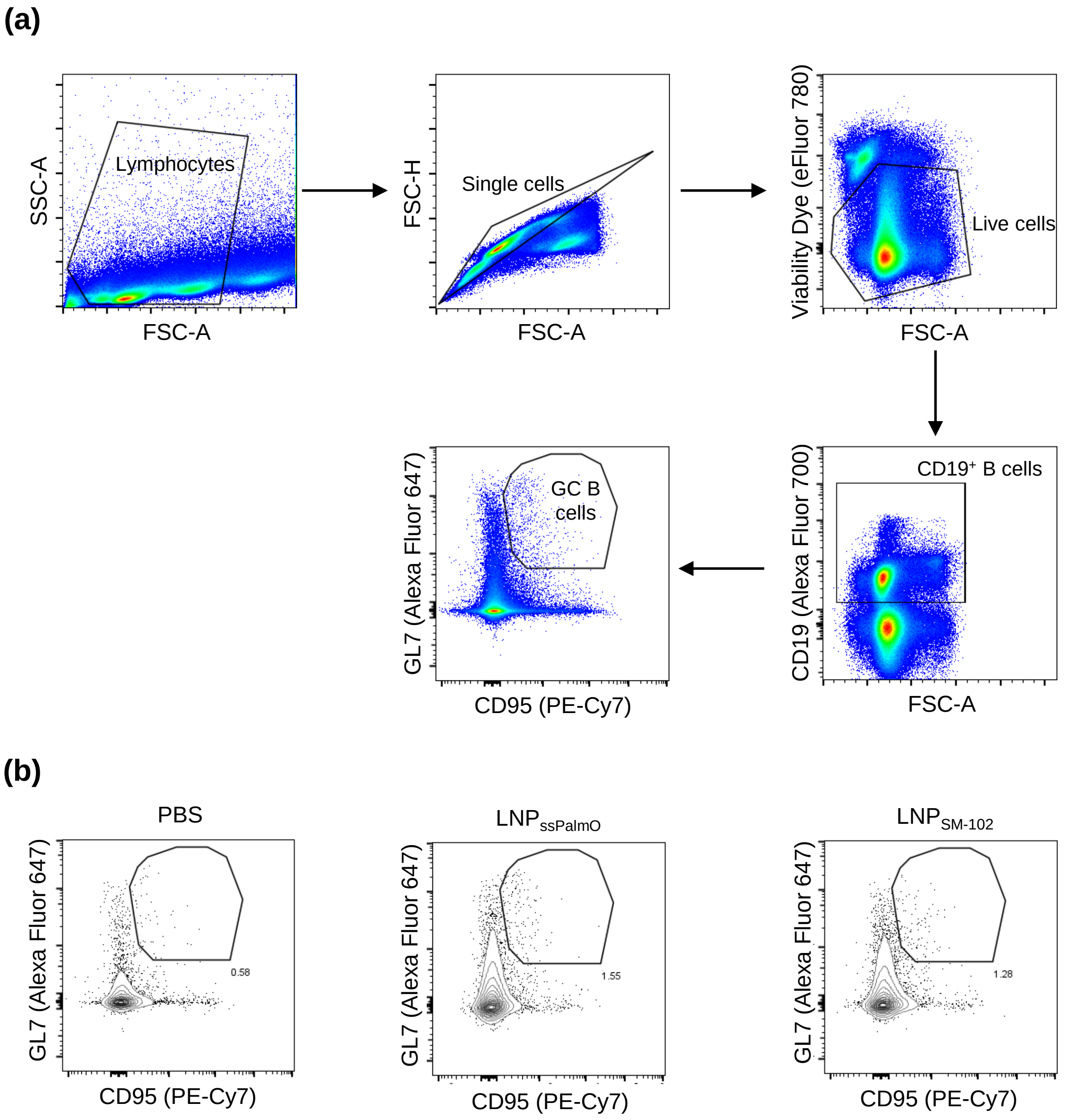

Figure S2. Flow cytometry analysis of germinal center B cells.
(a) Gating strategy and (b) representative plot of germinal center B cells. The data are related to those in Figure 2b.

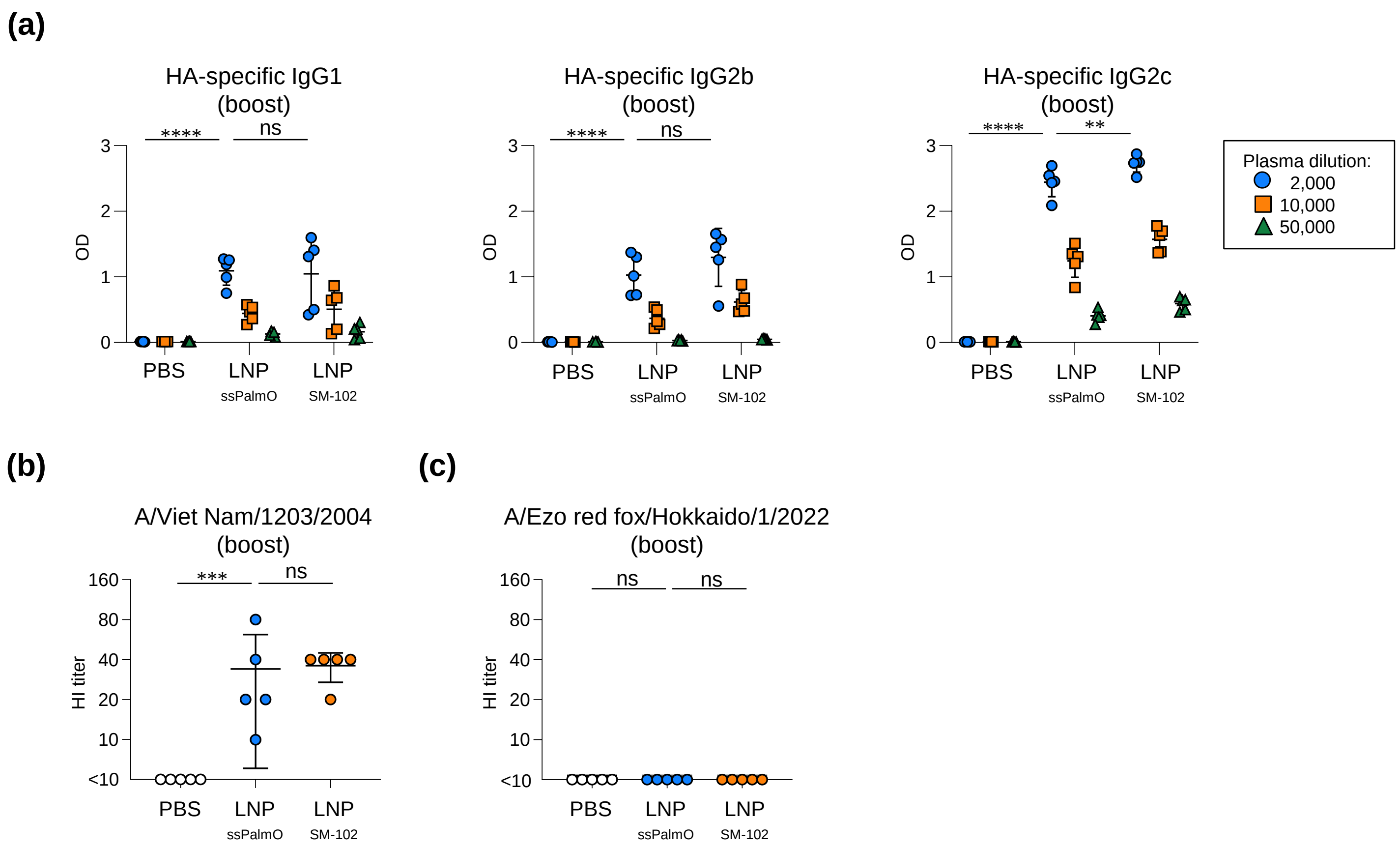

Figure S3. Antibody responses against HA following intramuscular immunization with HA-LNP. Mice were immunized intramuscularly with HA-LNP on days 0 (prime) and 21 (boost). (a) Plasma levels of HA-specific IgG1, IgG2b, and IgG2c on day 35 were analyzed using ELISA. (b, c) Hemagglutination inhibition titer of plasma on day 35 was assessed against H5N1 influenza A virus strains (b) A/Viet Nam/1203/2004 and (c) A/Ezo red fox/Hokkaido/1/2022. (a-c) n=5 per group. Data are means ± SD. $^{**}P < 0.01$; $^{***}P < 0.001$; $^{****}P < 0.0001$, Tukey's multiple-comparisons test .ns, not statistically significant. (a) Tukey's multiple comparison test was performed at a dilution of 2,000.

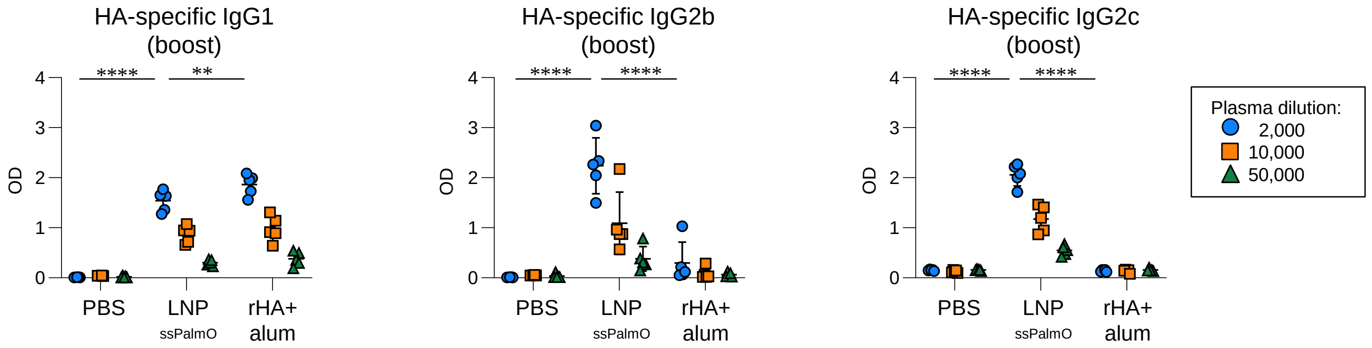

Figure S4. Comparison of antibody responses with rHA plus alum.
Mice were subcutaneously immunized with HA-LNP or rHA plus alum on days 0 (prime) and 21 (boost). Plasma levels of HA-specific IgG1, IgG2b, and IgG2c on day 35 were evaluated using ELISA. n=5 per group. Data are means ± SD. $^{**}P < 0.01$; $^{****}P < 0.0001$, Tukey's multiple-comparisons test. ns, not statistically significant. Tukey's multiple comparison test was performed at a dilution of 2,000.

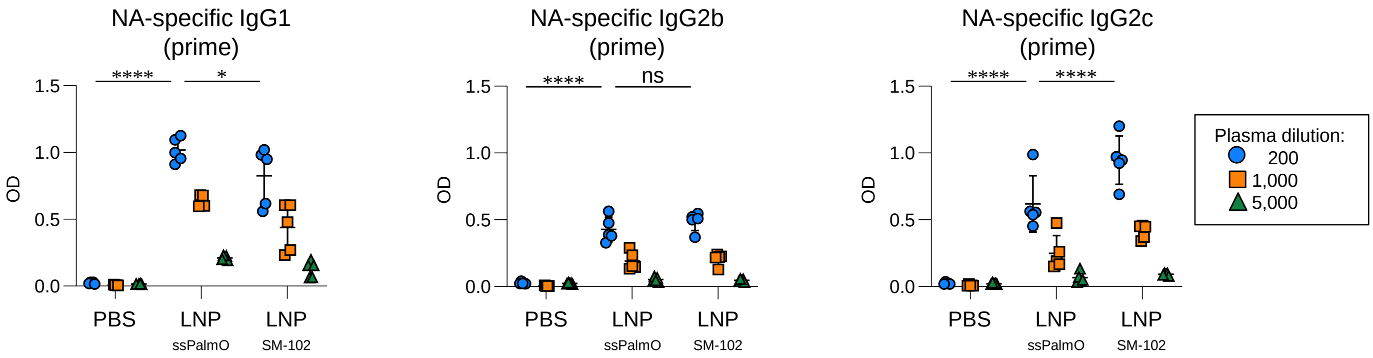

Figure S5. Antibody responses against NA following primary immunization with NA-LNP.
Mice were subcutaneously immunized with NA-LNP on day 0 (prime). The levels of NA-specific IgG1, IgG2b, and IgG2c in plasma on day 14 were measured using ELISA. The data are shown in Figure 2e. n=5 per group. Data are means ± SD. $^{*}P$ < 0.05; $^{****}P$ < 0.0001, Tukey's multiple-comparisons test. ns, not statistically significant. Tukey's multiple-comparison test was performed at a dilution of 200.

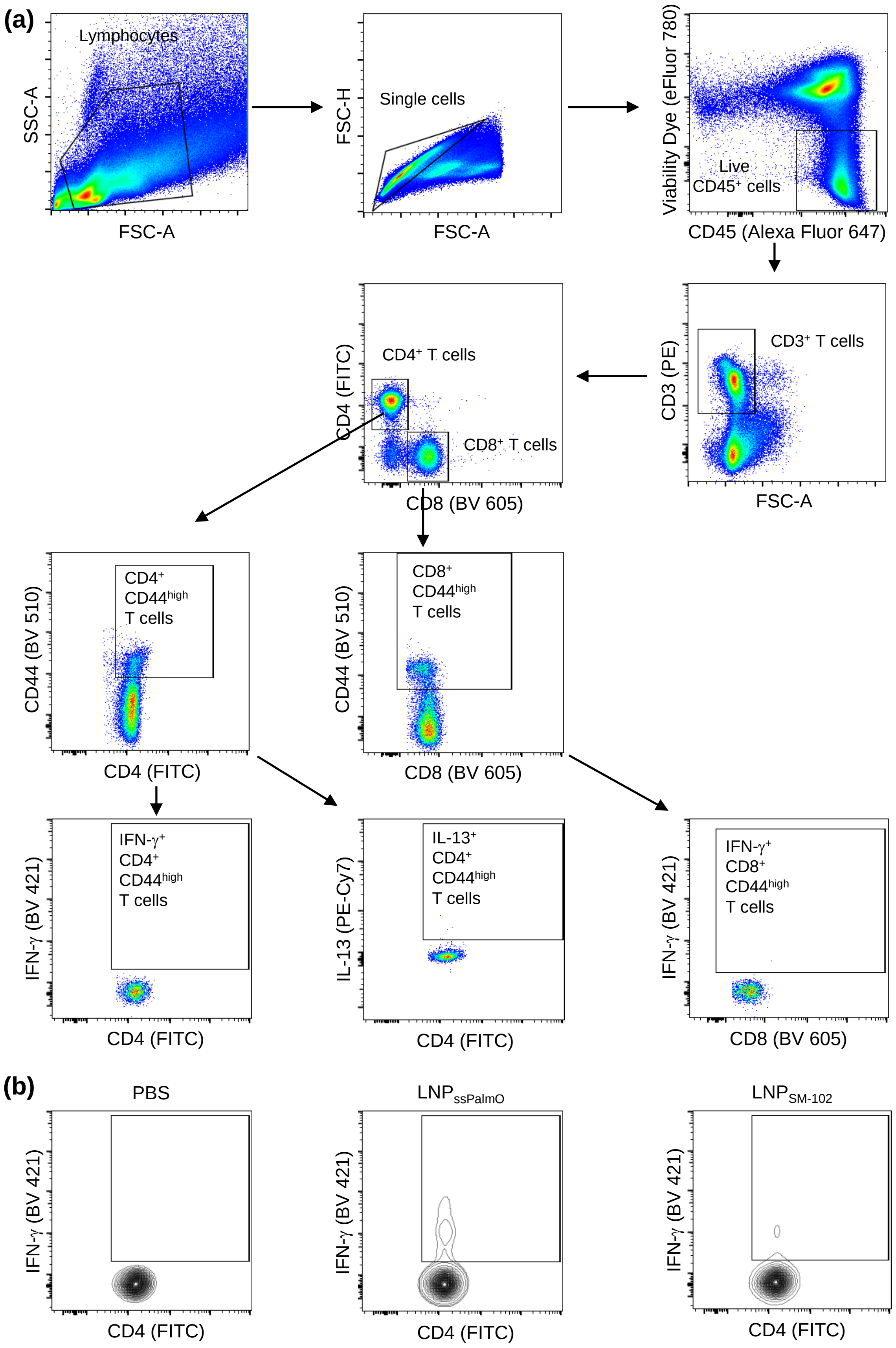

Figure S6. Flow cytometry analysis for cytokine-producing T cells.

(a) Gating strategy and (b) representative plot of IFN-γ^+ CD44^{high} CD4$^+$ T cells. The data are related to those in Figure 3.

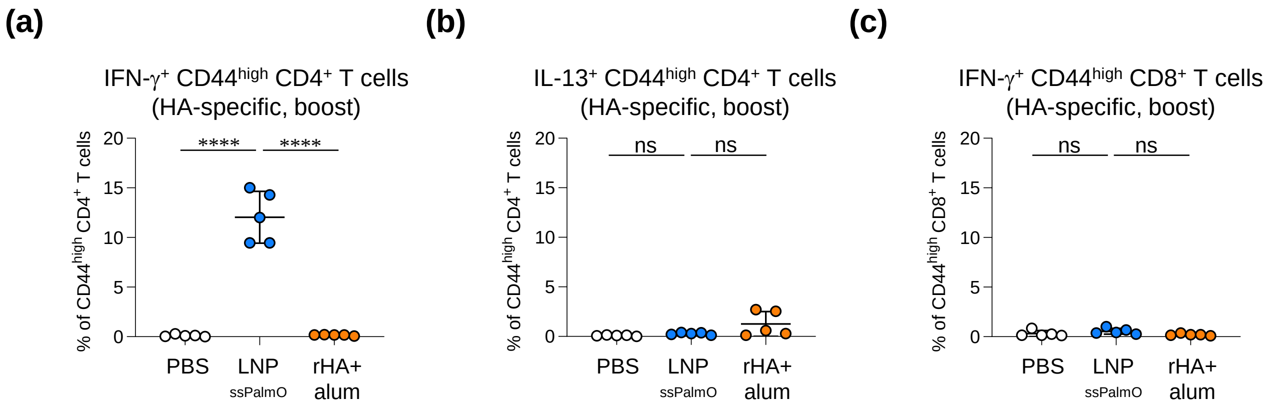

Figure S7. Comparison of T cell responses with rHA plus alum.
Mice were subcutaneously immunized with HA-LNP or rHA plus alum on days 0 (prime) and 21 (boost). On day 35, splenocytes from immunized mice were restimulated with HA. The intracellular cytokine levels in (a) IFN-γ^+ CD44^{high} CD4$^+$ T cells, (b) IL-13$^+$ CD44^{high} CD4$^+$ T cells, and (c) IFN-γ^+ CD44^{high} CD8$^+$ T cells were evaluated. (a-c) n=5 per group. The data represent the means ± SD. $^{****}P < 0.0001$, Tukey's multiple-comparisons test. ns, not statistically significant.

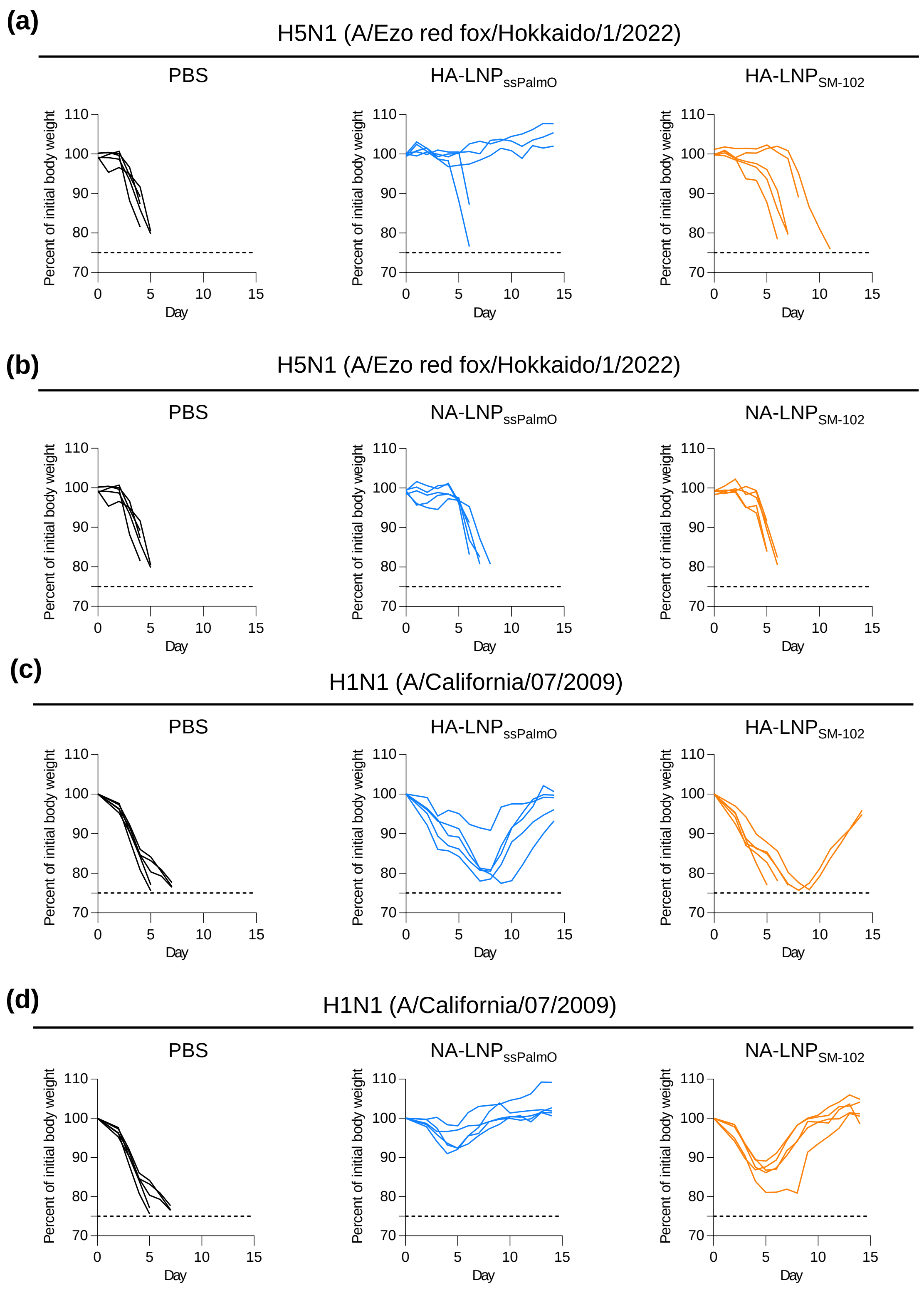

Figure S8. Body weight change in individual mouse after challenge with H5N1 or H1N1 influenza A virus after vaccination with mRNA-LNP.

Mice were subcutaneously immunized with (a, c) HA-LNP or (b, d) NA-LNP on day 0 (prime) and 21 (boost). On the day 35, the mice were intranasally challenged with (a, b) H5N1 A/Ezo red fox/Hokkaido/1/2022 or (c, d) H1N1 A/California/07/2009. Body weight loss in individual mouse was monitored for 14 days after viral challenge. The data are shown in Figure 4. (a-d) n=5 per group.

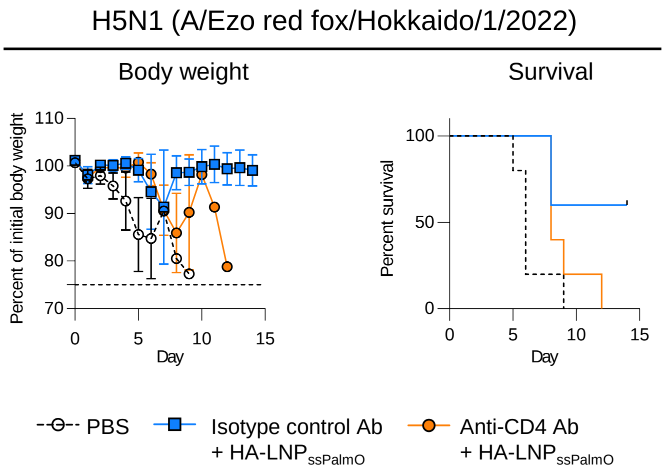

Figure S9. Contribution of $CD4^+$ T cells to cross-protection against heterologous H5N1 influenza virus in mice immunized with HA-LNP$_{ssPalmO}$.
Mice were subcutaneously immunized with HA-LNP$_{ssPalmO}$ on day 0 (prime) and day 21 (boost). On day 35, the mice were intranasally challenged with H5N1 A/Ezo red fox/Hokkaido/1/2022. $CD4^+$ T cells were depleted by anti-CD4 antibody on day 34 and 38. Body weight loss and survival of mice was monitored for 14 days after viral challenge. n=5 per group. Data presented as mean ± SD.

(a)

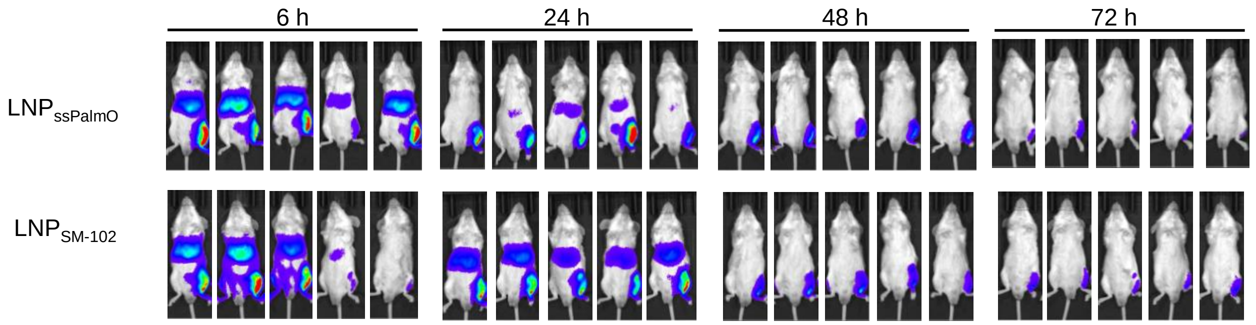

(b)

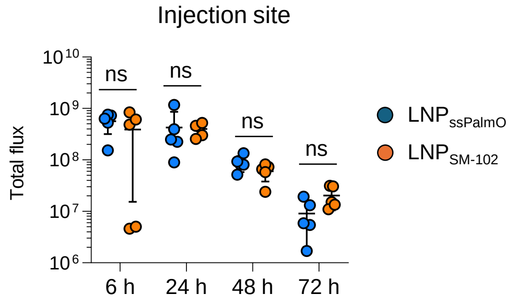

Figure S10. Antigen expression following repeated intramuscular injection with mRNA-LNP. Mice were intramuscularly injected with Luc-LNPs on days 0 (prime) and 21 (boost). At the indicated time points after the booster injection, luminescence was measured using *in vivo* imaging system. (a) Whole images captured on post injection 6, 24, 48, and 72 h. (b) Total flux at the injection site for all images was quantitated. (a-b) n=5 per group. Data are means ± SD. ns, not statistically significant.

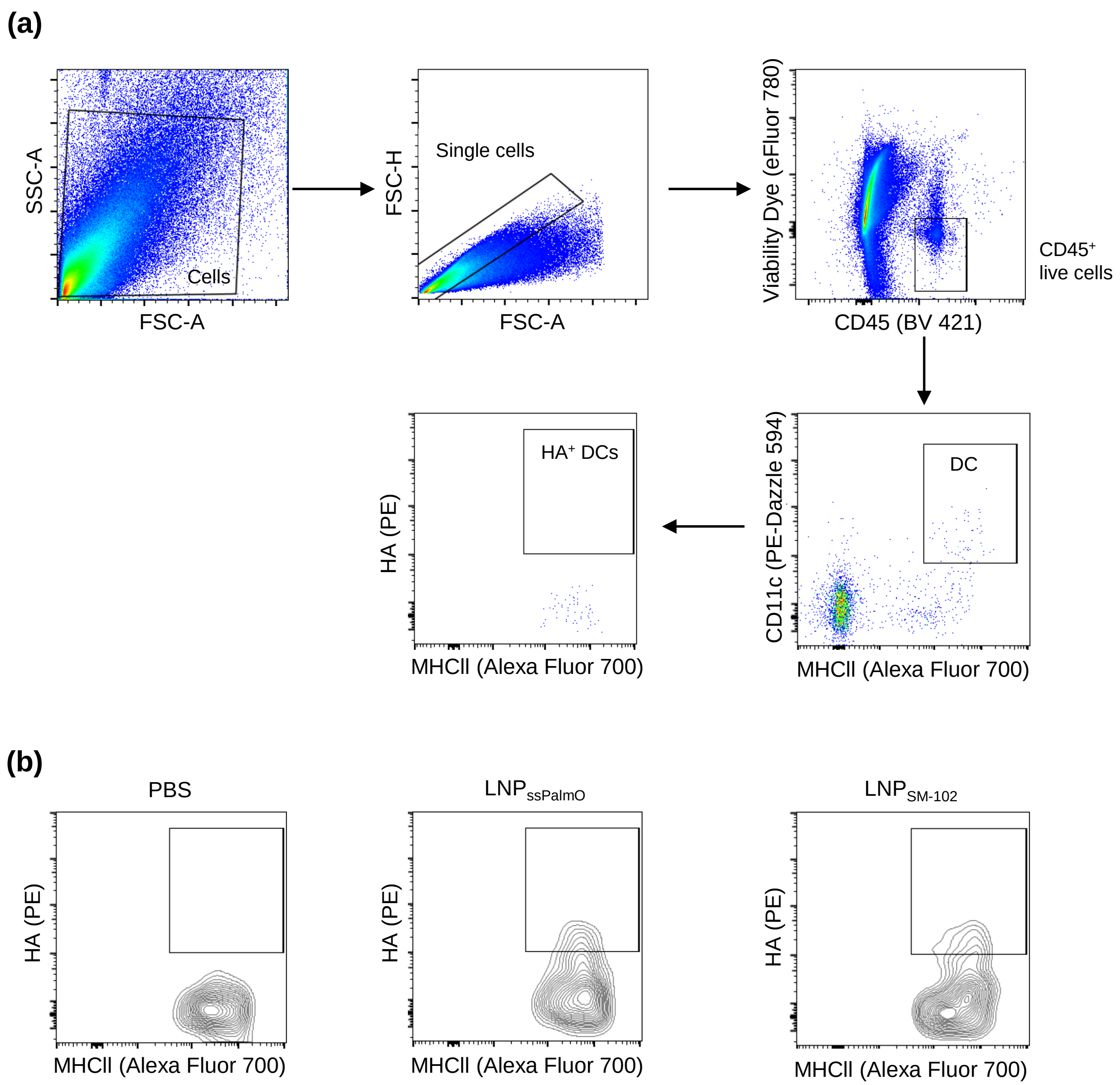

Figure S11. Flow cytometry analysis of HA expression in DCs.
(a) Gating strategy and (b) representative plot of HA^+ DCs. The data are related to those in Figure 5h.

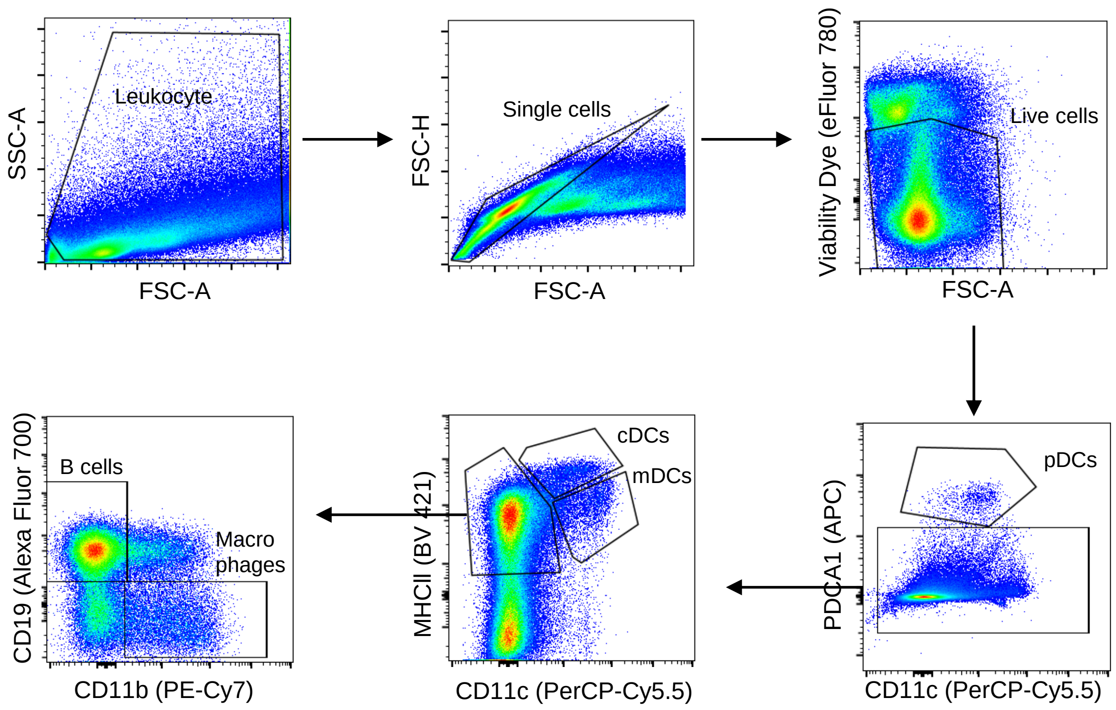

Figure S12. Gating strategy for the flow cytometry analysis of antigen-presenting cell activation. The data are related to those in Figure 6.

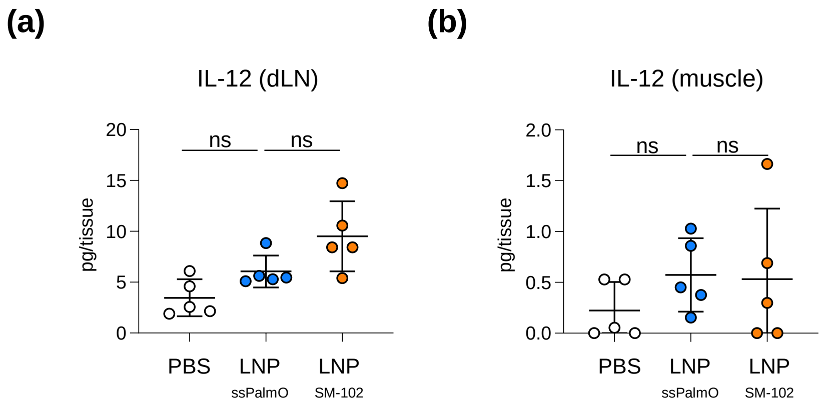

Figure S13. IL-12 levels following immunization with mRNA-LNP.
Mice were (a) subcutaneously or (b) intramuscularly immunized with HA-LNP on day 0. At 6 h after immunization, the level of IL-12 in (a) dLN or (b) muscle were measured. (a, b) n=5 per group. Data are presented as mean ± SD. ns, not statistically significant.

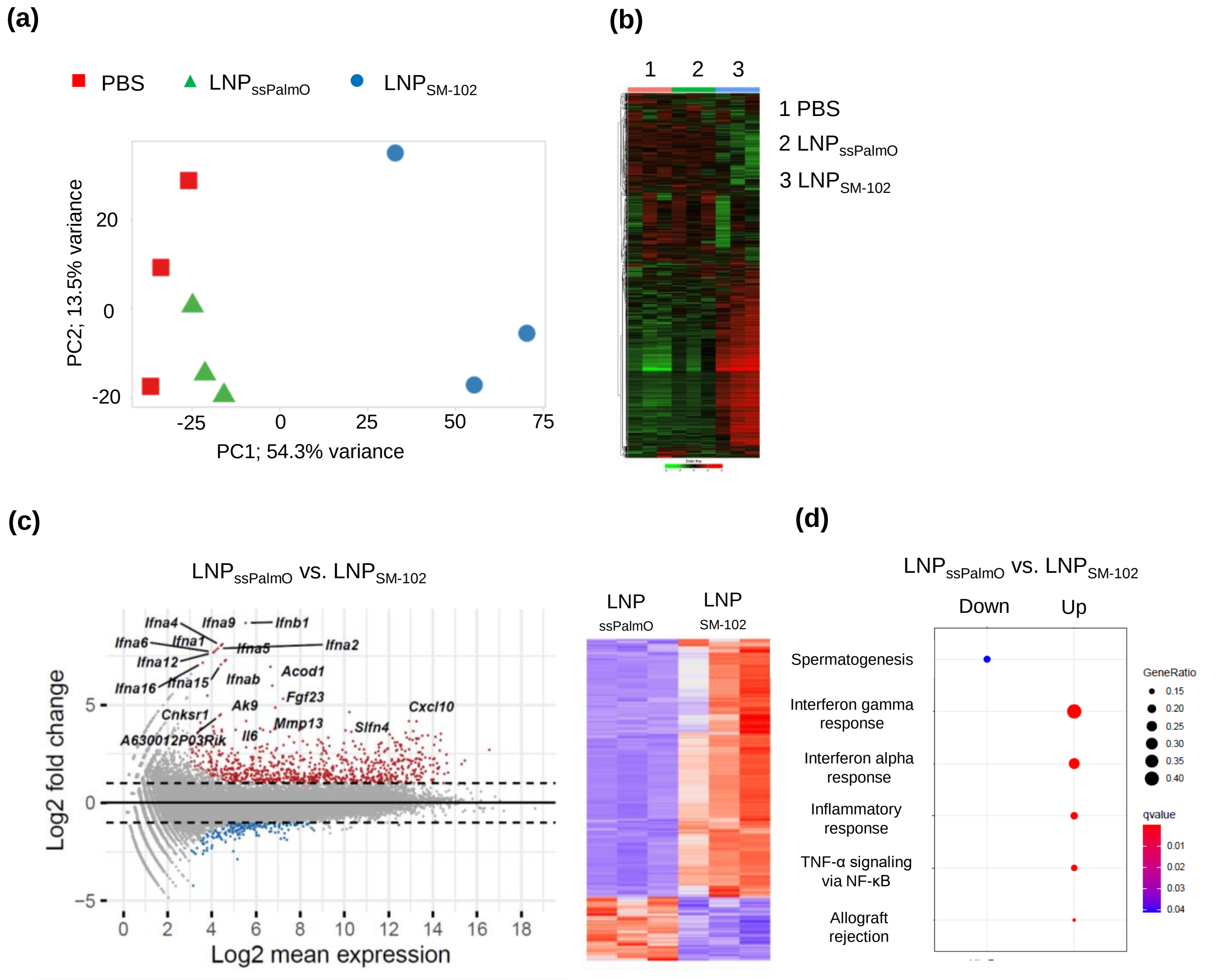

Figure S14. Transcriptional signatures in draining lymph nodes following subcutaneous immunization with mRNA-LNP.

Mice were subcutaneously immunized with HA-LNP and draining lymph nodes were analyzed using RNA sequencing after 6 h. (a) Principal component analysis of differentially expressed genes (DEGs). (b) Heatmap analysis of the expression of the top 2000 DEGs. (c) Mean-average (MA) plot and (d) enrichment analysis of DEGs at 6 h. (a-d) n = 3 per group.

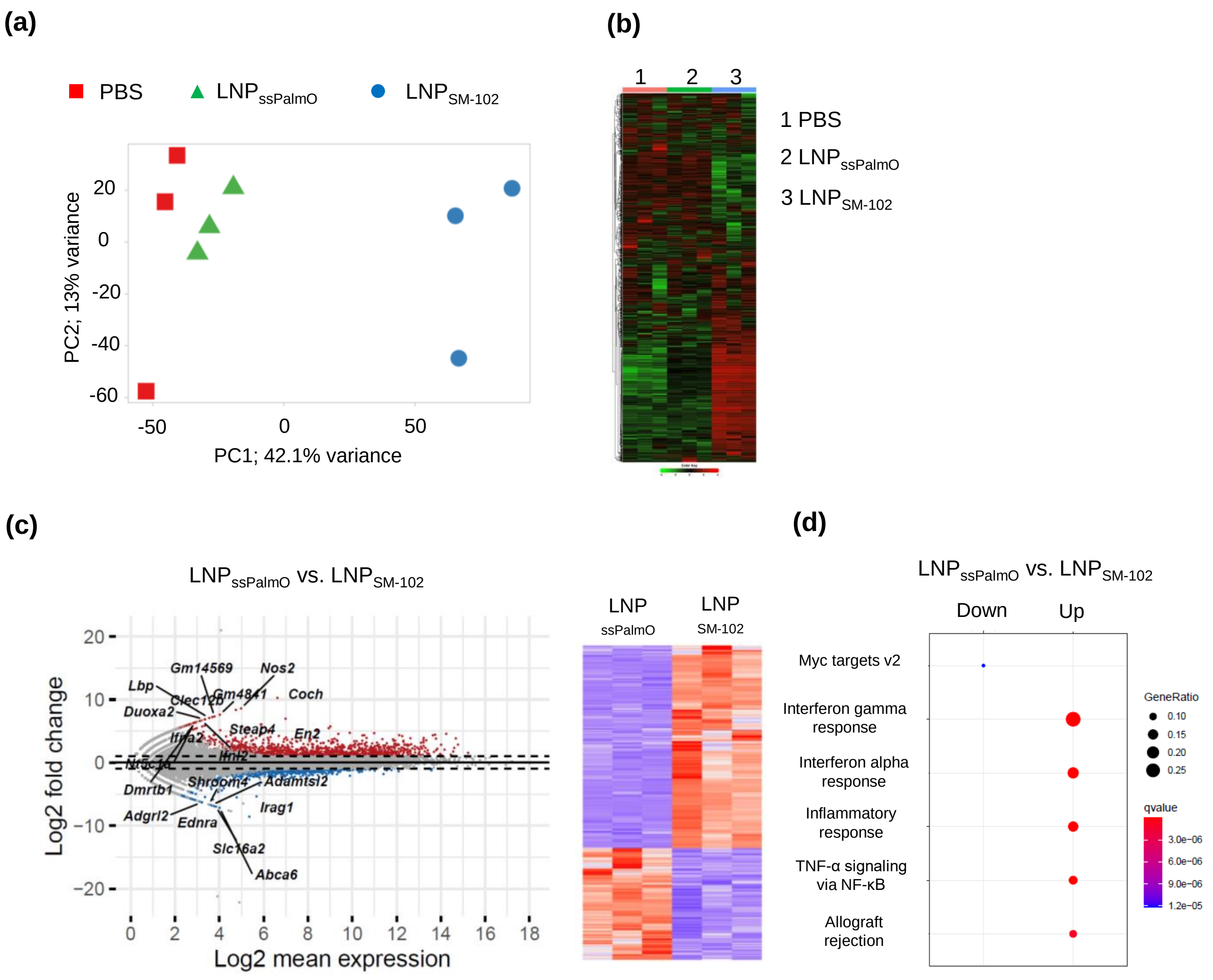

Figure S15. Transcriptional signatures in DCs from draining lymph nodes following subcutaneous immunization with mRNA-LNP.
Mice were subcutaneously immunized with HA-LNP. After 6 h, DCs from draining lymph nodes were analyzed using RNA sequencing. (a) Principal component analysis of DEGs. (b) Heatmap analysis of expression of the top 2000 DEGs. (c) MA plot and (d) enrichment analysis of DEGs at 6 h. (a-d) n = 3 per group.

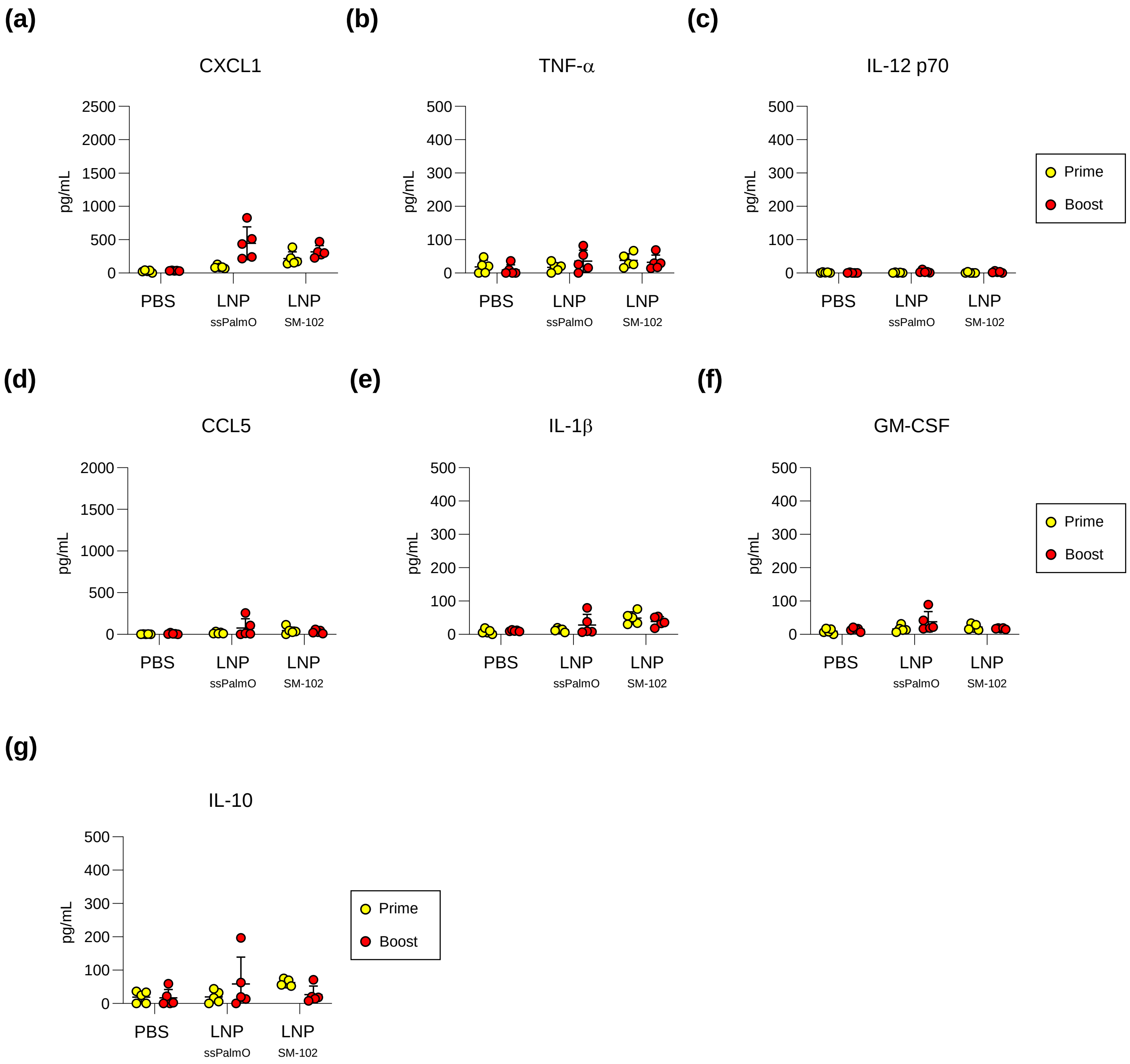

Figure S16. Inflammatory cytokine levels following subcutaneous immunization with mRNA-LNP. Mice were subcutaneously immunized with HA-LNP on days 0 (prime) and 21 (boost). (a-g) At 6 h after primary and booster immunization, the level of (a) CXCL1, (b) TNF-α, (c) IL-12 p70, (d) CCL5, (e) IL-1β, (f) GM-CSF, and (g) IL-10 in plasma were measured. (a-g) n=5 per group. Data are means ± SD.

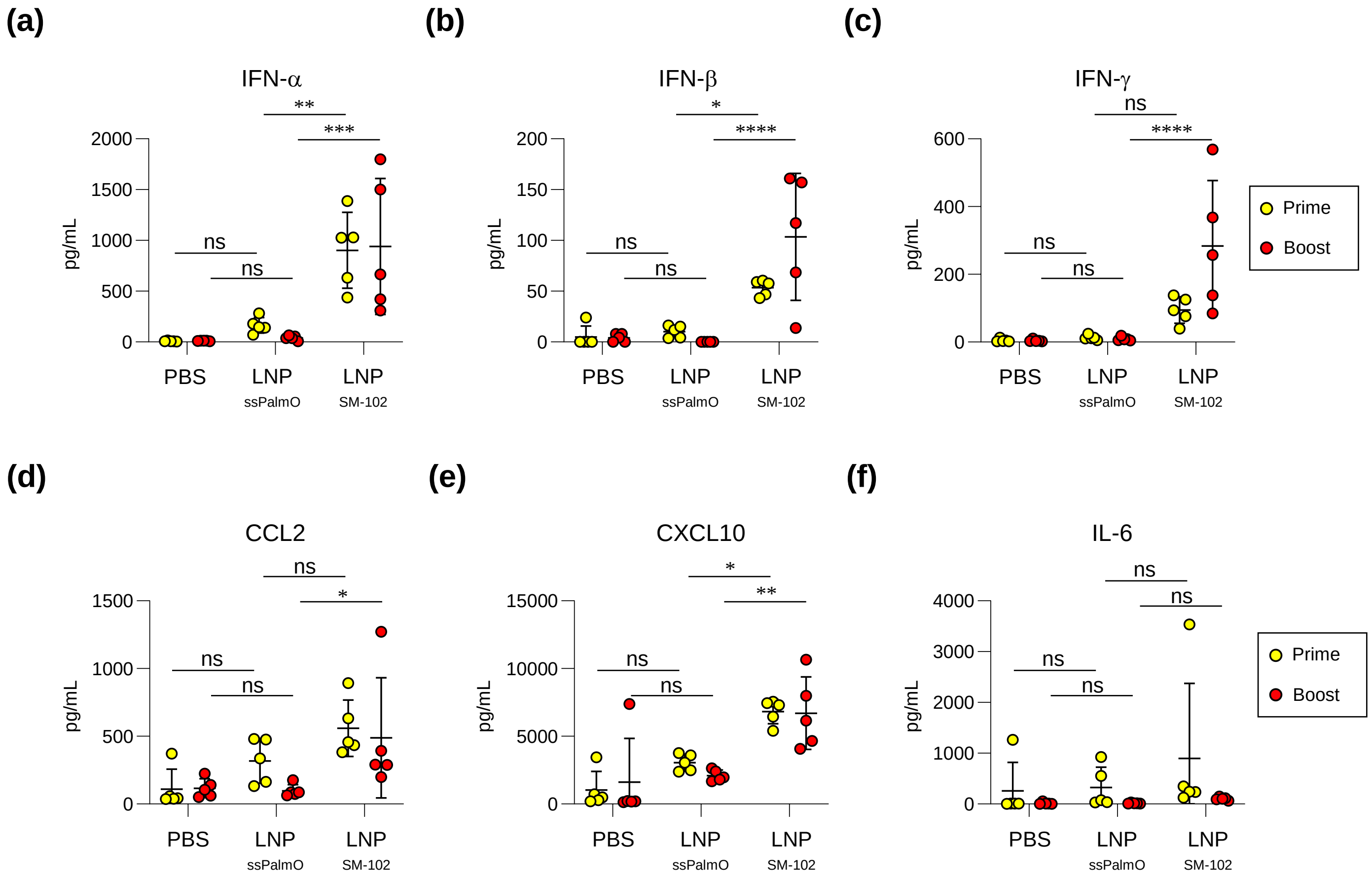

Figure S17. Inflammatory cytokines in the blood following intramuscular immunization with mRNA-LNP.

Mice were immunized intramuscularly with HA-LNP on days 0 (prime) and 21 (boost). At 6 h after primary and booster immunizations, the levels of (a) IFN-α, (b) IFN-β, (c) IFN-γ, (d) CCL2, (e) CXCL10, and (f) IL-6 were measured. (a-f) n=5 per group. Data are means ± SD. $^{*}P < 0.05$; $^{**}P < 0.01$; $^{***}P < 0.001$; $^{****}P < 0.0001$, Tukey's multiple-comparisons test. ns, not statistically significant.

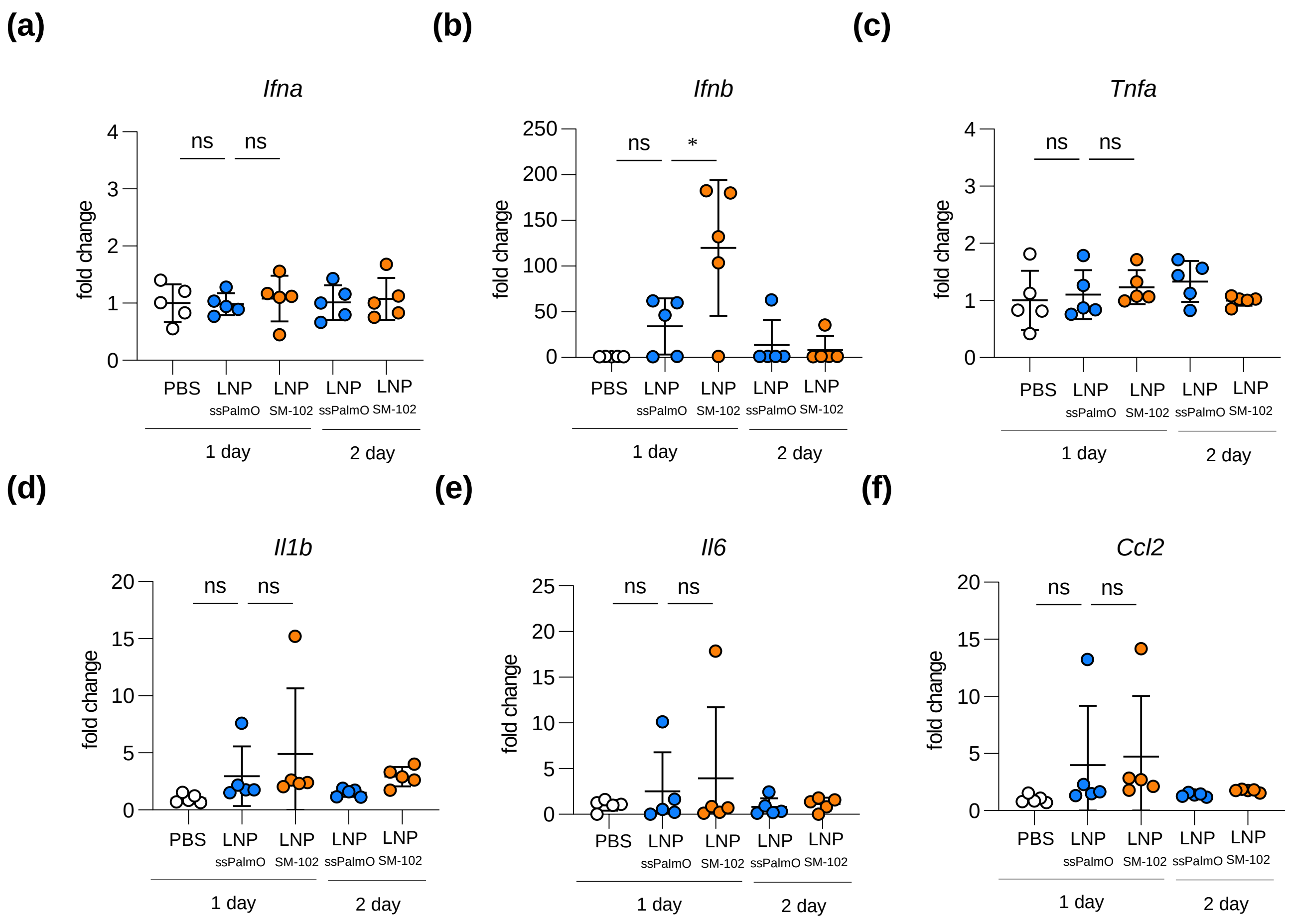

Figure S18. Inflammatory cytokines in heart tissues following intramuscular immunization with mRNA-LNP.

Mice were immunized intramuscularly with spike-LNP on day 0 (prime). At days 1 and 2 after primary immunizations, the mRNA levels of (a) *Ifna*, (b) *Ifnb*, (c) *Tnfa*, (d) *Il1b*, (e) *Il6*, and (f) *Ccl2* were measured. (a-f) n=5 per group. Data are presented as mean ± SD. $^{*}P < 0.05$; Tukey's multiple-comparisons test. ns, not statistically significant.

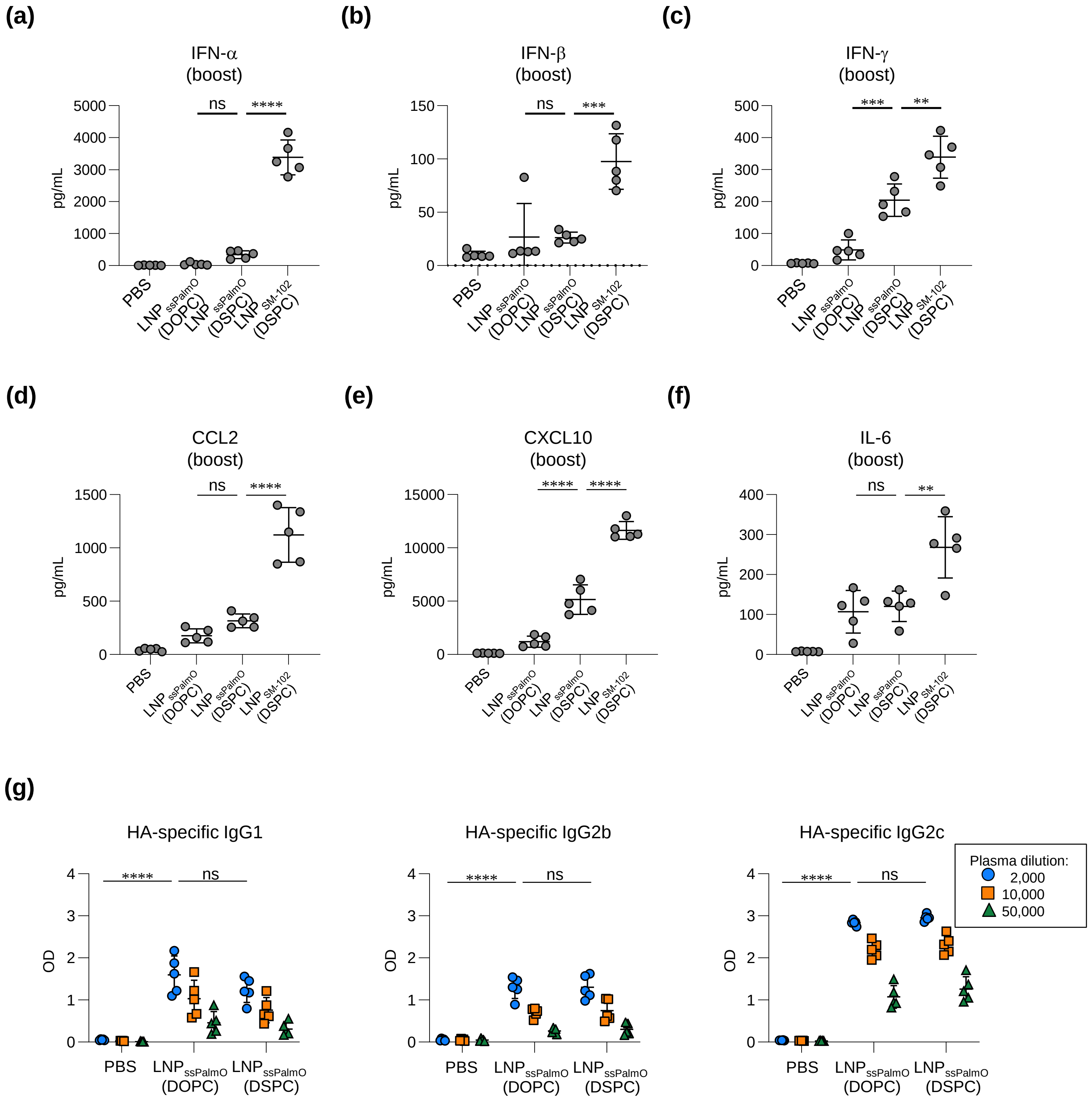

Figure S19. Comparison of inflammatory cytokines and antibody responses between mRNA-LNP$_{ssPalmO}$ with DSPC and mRNA-LNP$_{ssPalmO}$ with DOPC.

Mice were subcutaneously immunized with HA-LNP$_{ssPalmO}$ with DOPC, LNP$_{ssPalmO}$ with DSPC, or LNP$_{SM\text{-}102}$ with DSPC on days 0 (prime) and 21 (boost). (a-f) Blood was collected 6 h after boosting. Levels of (a) IFN-α, (b) IFN-β, (c) IFN-γ, (d) CCL2, (e) CXCL10, and (f) IL-6 were measured. (g) Plasma levels of HA-specific IgG1, IgG2b, and IgG2c on day 35 were evaluated using ELISA. (a-g) n=5 per group. Data are means ± SD. $^{**}P < 0.01$; $^{***}P < 0.001$; $^{****}P < 0.0001$, Tukey's multiple-comparisons test. ns, not statistically significant. (g) Tukey's multiple comparison test was performed at a dilution of 2,000.

ATGGAAAAGATCGTGCTGCTGTTCGCCATCGTGTCCCTGGTCAAGAGCGACCAGAT
CTGCATCGGCTACCACGCCAACAACAGCACCGAACAGGTGGACACCATCATGGAAA
AGAATGTGACCGTGACACACGCCCAGGACATCCTCGAGAAGAAGCACAACGGCAA
GCTGTGCGACCTGGACGGCGTGAAGCCTCTGATCCTGAGAGACTGTTCTGTGGCC
GGATGGCTGCTGGGCAACCCTATGTGTGACGAGTTCATCAACGTGCCCGAGTGGTC
CTACATCGTGGAAAAGGCTAACCCCGTGAACGACCTGTGCTACCCCGGCGACTTCA
ACGACTACGAGGAACTGAAGCACCTCCTGAGCAGAATCAACCACTTCGAGAAGATC
CAGATCATCCCCAAGAGCAGCTGGTCCTCTCACGAGGCTTCTCTGGGAGTGTCTAG
CGCATGTCCATACCAGGGCAAGTCCAGCTTCTTCAGAAACGTCGTGTGGCTGATCA
AGAAGAACAGCACATACCCCACCATCAAGCGGAGCTACAACAACACCAATCAAGAG
GACCTGCTGGTGCTGTGGGGCATCCACCATCCTAACGATGCCGCCGAGCAGACCA
AGCTGTACCAGAATCCTACCACCTACATCAGCGTGGGCACCAGCACACTGAACCAG
AGACTGGTGCCTAGAATCGCCACCAGATCCAAAGTGAACGGCCAGAGCGGCAGAAT
GGAATTCTTCTGGACCATCCTGAAGCCTAACGACGCCATCAACTTCGAGAGCAACG
GCAACTTTATCGCCCCTGAGTACGCCTACAAGATCGTGAAGAAGGGCGACAGCACA
ATCATGAAGTCCGAGCTGGAATACGGCAACTGCAACACCAAGTGTCAGACCCCTATG
GGCGCTATCAACTCCAGCATGCCCTTCCACAACATCCATCCTCTGACCATCGGCGA
GTGCCCCAAATACGTGAAGTCCAACAGACTGGTCCTGGCCACCGGCCTGAGAAACT
CTCCACAGCGCGAGCGCAGAAGAAAGAAGAGAGGCCTGTTCGGCGCTATCGCCGG
CTTTATTGAAGGCGGCTGGCAAGGCATGGTGGACGGATGGTACGGATACCACCACA
GCAACGAGCAAGGCTCTGGCTACGCCGCCGACAAAGAGAGCACACAGAAAGCCAT
CGACGGCGTCACCAACAAAGTGAATAGCATCATCGACAAGATGAACACCCAGTTCG
AGGCCGTGGGCAGAGAGTTCAACAACCTGGAAAGAAGGATCGAGAACCTGAACAA
GAAAATGGAAGATGGCTTCCTGGACGTGTGGACCTACAACGCTGAGCTGCTCGTGC
TGATGGAAAACGAGAGAACCCTGGACTTCCACGACTCCAACGTGAAGAACCTGTAC
GACAAAGTGCGGCTGCAGCTGAGAGACAACGCCAAAGAACTCGGCAACGGCTGCT
TCGAGTTCTACCACAAGTGCGACAACGAGTGCATGGAAAGCGTGCGGAACGGCAC
CTACGACTACCCTCAGTACAGCGAGGAAGCCAGACTGAAGAGGGAAGAGATCAGC
GGAGTGAAGCTGGAATCCATCGGCATCTACCAGATCCTGAGCATCTACAGCACCGT
GGCCTCTTCTCTGGCCCTGGCTATTATGGTGGCTGGCCTGAGCCTGTGGATGTGCT
CTAACGGCAGCCTGCAGTGCAGAATCTGCATCTGA

Figure S20. Sequence for H5N1 HA.

ATGAACCCCAACCAGAAGATCATCACCATCGGCAGCATCTGCATGGTCACCGGCATC
GTGTCTCTGATGCTGCAGATCGGCAACATGATCAGCATCTGGGTGTCCCACAGCATC
CACACCGGAAACCAGCACCAGAGCGAGCCTATCAGCAACACCAACTTTCTGACCGA
GAAGGCCGTGGCCTCTGTGAAGCTGGCTGGCAACTCTAGCCTGTGTCCTATCAACG
GCTGGGCCGTGTACAGCAAGGACAACAGCATCAGGATCGGCTCCAAGGGCGACGT
GTTCGTGATCAGAGAGCCCTTCATCAGCTGCAGCCACCTGGAATGCAGAACATTCTT
CCTGACACAAGGCGCCCTGCTGAACGACAAGCACAGCAACGGCACCGTGAAGGAC
AGAAGCCCTCACAGAACCCTGATGAGCTGCCCTGTGGGCGAAGCCCCATCTCCTTA
CAACAGCAGATTCGAGTCCGTGGCTTGGAGCGCCTCTGCTTGTCACGATGGCACCA
GCTGGCTGACAATCGGCATCAGCGGACCTGATAACGGCGCTGTGGCTGTGCTGAA
GTACAACGGCATCATCACCGACACCATCAAGAGCTGGCGGAACAACATCCTGAGAA
CCCAAGAGTCCGAGTGCGCCTGTGTGAACGGCAGCTGTTTCACCGTGATGACAGA
CGGCCCTTCTAACGGCCAGGCCAGCCACAAGATCTTCAAGATGGAAAAGGGCAAAG
TGGTCAAGAGCGTGGAACTGGACGCCCCTAACTACCACTACGAGGAATGCAGCTGC
TACCCCAACGCCGGCGAGATCACCTGTGTGTGCAGAGATAACTGGCACGGCAGCAA
CAGACCTTGGGTGTCCTTCAACCAGAACCTGGAATACCAGATCGGCTATATCTGCAG
CGGCGTGTTCGGCGACAACCCCAGACCTAATGATGGCACAGGCAGCTGCGGACCC
GTGTCTAGCAATGGTGCTTACGGCGTGAAGGGCTTCAGCTTTAAGTACGGCAACGG
CGTGTGGATCGGCAGGACCAAGAGCACCAACTCCAGATCCGGCTTCGAGATGATCT
GGGACCCTAACGGCTGGACCGAGACAGACAGCAGCTTCAGCGTGAAGCAGGACAT
CGTGGCCATCACCGATTGGAGCGGCTACAGCGGCTCTTTCGTGCAGCACCCTGAAC
TGACAGGCCTGGACTGCATCAGACCCTGTTTCTGGGTCGAGCTGATCAGAGGCAGA
CCCAAAGAGAGCACCATCTGGACCAGCGGCAGCAGCATCTCTTTCTGCGGCGTGAA
CAGCGACACCGTCGGATGGTCTTGGCCTGATGGTGCTGAGCTGCCTTTCACCATCG
ACAAGTGA

Figure S21. Sequence for H5N1 NA.

Table S1. Antibodies used for ELISA and flow cytometry analysis.

ELISA

	Clone	Catalog number	Dilution	Source
HRP anti-mouse IgG1		1070-05	1:8000	SouthernBiotech
HRP anti-mouse IgG2b		1090-05	1:5000	SouthernBiotech
HRP anti-mouse IgG2c		1078-05	1:8000	SouthernBiotech

Germinal center B cell analysis

	Clone	Catalog number	Dilution	Source
Anti-mouse CD16/CD32	93	101302	1:200	BioLegend
Fixable Viability Dye eFluor 780		65-0865-18	1:1000	Thermo Fisher Scientific
Alexa Fluor 647 anti-mouse/human GL7	GL7	144606	1:200	BioLegend
Alexa Fluor 700 anti-mouse CD19	6D5	115528	1:200	BioLegend
PE/Cy7 anti-mouse CD95	SA367H8	152618	1:200	BioLegend

Intracellular cytokine in T cells

	Clone	Catalog number	Dilution	Source
Anti-mouse CD16/CD32	93	101302	1:200	BioLegend
Fixable Viability Dye eFluor 780		65-0865-18	1:1000	Thermo Fisher Scientific
Alexa Fluor 647 anti-mouse CD45	30-F11	103124	1:200	BioLegend
PE anti-mouse CD3	145-2C11	100308	1:200	BioLegend
FITC anti-mouse CD4	GK1.5	100406	1:200	BioLegend
BV 605 anti-mouse CD8a	53-6.7	100744	1:200	BioLegend
BV 510 anti-mouse CD44	IM7	103044	1:200	BioLegend
BV 421 anti-IFN-γ	XMG1.2	505830	1:200	BioLegend
PE/Cy7 anti-IL-13	eBio13A	25-7133-82	1:200	Thermo Fisher Scientific

HA expression

	Clone	Catalog number	Dilution	Source
Anti-mouse CD16/CD32 antibody	93	101302	1:200	BioLegend
Fixable Viability Dye eFluor 780		65-0865-18	1:1000	Thermo Fisher Scientific
BV421 anti-mouse CD45	30-F11	103134	1:200	BioLegend
Alexa Fluor 700 anti-mouse I-A/I-E	M5/114.15.2	107622	1:200	BioLegend
PE-Dazzle 594 anti-mouse CD11c	N418	117348	1:200	BioLegend
anti-HA		11062-T62	1:1000	Sino Biological
PE anti-rabbit IgG	Poly4064	406421	1:1000	BioLegend

Activation of antigen presenting cells

	Clone	Catalog number	Dilution	Source
Anti-mouse CD16/CD32 antibody	93	101302	1:200	BioLegend
Fixable Viability Dye eFluor 780		65-0865-18	1:1000	Thermo Fisher Scientific
PerCP/Cy5.5 anti-mouse CD11c	N418	117328	1:200	BioLegend
APC anti-mouse PDCA1	927	127016	1:200	BioLegend
Alexa Fluor 700 anti-mouse CD19	6D5	115528	1:200	BioLegend
BV421 anti-mouse I-A/I-E	M5/114.15.2	107632	1:200	BioLegend
PE anti-mouse CD86	GL-1	105008	1:200	BioLegend
PE/Cy7 anti-mouse CD11b	M1/70	101216	1:200	BioLegend

Isolation of DCs

	Clone	Catalog number	Dilution	Source
Fixable Viability Dye eFluor 780		65-0865-18	1:1000	Thermo Fisher Scientific
Alexa Fluor 488 anti-CD90.2	30-H12	105316	1:500	BioLegend
Alexa Fluor 700 anti-mouse I-A/I-E	M5/114.15.2	107622	1:200	BioLegend
PE-Dazzle 594 anti-mouse CD11c	N418	117348	1:200	BioLegend
PE-Cy7 anti-mouse CD19	6D5	115520	1:200	BioLegend
BV421 anti-mouse CD45	30-F11	103134	1:200	BioLegend

Table S2. Primers for RT-PCR.

	Forward	Reverse
Ifna	5′-GGACTTTGGATTCCCGCAGGAGAAG-3′	5′-GCTGCATCAGACAGCCTTGCAGGTC-3′
Ifnb	5′-AACCTCACCTACAGGGCGGACTTCA-3′	5′-TCCCACGTCAATCTTTCCTCTTGCTTT-3′
Tnfa	5′-CCTCTCATGCACCACCATCAA-3′	5′-TTCTGAGACAGAGGCAACCTG-3′
Il1b	5′-TCTTTGAAGTTGACGGACCC -3′	5′-TGAGTGATACTGCCTGCCTG-3′
Il6	5′-CTGTAGCTCATTCTGCTCTGGA-3′	5′-CAACTGGATGGAAGTCTCTTGC-3′
Ccl2	5′-CACTCACCTGCTGCTACTCA-3′	5′-TCTTGAGCTTGGTGACAAAAACTAC-3′
Gapdh	5′-CAGGTTGTCTCCTGCGACTT-3′	5′-AGCCGTATTCATTGTCATACCAGG-3′
