# Supplementary material for: Low-inflammatory lipid nanoparticle-based mRNA vaccine elicits protective immunity against H5N1 influenza virus with reduced adverse reactions
Source: Mol Ther. 2024 Dec 17;33(2):529–47. doi: 10.1016/j.ymthe.2024.12.032 (PMC11852987; doi:10.1016/j.ymthe.2024.12.032)
Supplement: Document S2. Article plus supplemental information [file mmc2.pdf]

Molecular Therapy
Original Article

Low-inflammatory lipid nanoparticle-based mRNA vaccine elicits protective immunity against H5N1 influenza virus with reduced adverse reactions

Atsushi Kawai,[1,2,14] Taro Shimizu,[2,3,4,14] Hiroki Tanaka,[4,5] Shintaro Shichinohe,[6] Jessica Anindita,[5] Mika Hirose,[7] Eigo Kawahara,[1,2] Kota Senpuku,[1,2] Makoto Shimooka,[6] Le Thi Quynh Mai,[8] Ryo Suzuki,[9] Takuto Nogimori,[10] Takuya Yamamoto,[10] Toshiro Hirai,[1,2,3,4] Takayuki Kato,[4,7] Tokiko Watanabe,[4,6,11] Hidetaka Akita,[4,5] and Yasuo Yoshioka[1,2,3,4,11,12,13]

[1]Laboratory of Nano-design for Innovative Drug Development, Graduate School of Pharmaceutical Sciences, Osaka University, 1-6 Yamadaoka, Suita, Osaka 565-0871, Japan; [2]Vaccine Creation Group, BIKEN Innovative Vaccine Research Alliance Laboratories, Research Institute for Microbial Diseases, Osaka University, 3-1 Yamadaoka, Suita, Osaka 565-0871, Japan; [3]Vaccine Creation Group, BIKEN Innovative Vaccine Research Alliance Laboratories, Institute for Open and Transdisciplinary Research Initiatives, Osaka University, 3-1 Yamadaoka, Suita, Osaka 565-0871, Japan; [4]Center for Advanced Modalities and DDS, Osaka University, 3-1 Yamadaoka, Suita, Osaka 565-0871, Japan; [5]Laboratory of DDS Design and Drug Disposition, Graduate School of Pharmaceutical Sciences, Tohoku University, 6-3 Aoba, Aramaki, Aoba-ku, Sendai City, Miyagi 980-8578, Japan; [6]Department of Molecular Virology, Research Institute for Microbial Diseases, Osaka University, 3-1 Yamadaoka, Suita, Osaka 565-0871, Japan; [7]Laboratory for Cryo-EM Structural Biology, Institute for Protein Research, Osaka University, 3-2 Yamadaoka, Suita, Osaka 565-0871, Japan; [8]Department of Virology, National Institute of Hygiene and Epidemiology, No. 1 Yersin Street, Hanoi 100000, Vietnam; [9]Laboratory of Drug and Gene Delivery Research, Faculty of Pharmaceutical Sciences, Teikyo University, 2-11-1 Kaga, Itabashi, Tokyo 173-8605, Japan; [10]Laboratory of Precision Immunology, Center for Intractable Diseases and ImmunoGenomics, National Institutes of Biomedical Innovation, Health and Nutrition, 7-6-8 Saito-Asagi, Ibaraki, Osaka 567-0085, Japan; [11]Center for Infectious Disease Education and Research, Osaka University, 3-1 Yamadaoka, Suita, Osaka 565-0871, Japan; [12]Global Center for Medical Engineering and Informatics, Osaka University, 3-1 Yamadaoka, Suita, Osaka 565-0871, Japan; [13]Vaccine Creation Group, BIKEN Innovative Vaccine Research Alliance Laboratories, The Research Foundation for Microbial Diseases of Osaka University, 3-1 Yamadaoka, Suita, Osaka 565-0871, Japan

Messenger RNA vaccines based on lipid nanoparticles (mRNA-LNPs) are promising vaccine modalities. However, mRNA-LNP vaccines frequently cause adverse reactions such as swelling and fever in humans, partly due to the inflammatory nature of LNP. Modification of the ionizable lipids used in LNPs is one approach to avoid these adverse reactions. Here, we report the development of mRNA-LNP vaccines with better protective immunity and reduced adverse reactions using LNPs, which contain a disulfide (SS)-cleavable bond and pH-activated lipid-like materials with oleic acid (ssPalmO) as an ionizable lipid (LNP$_{ssPalmO}$). We used mRNA expressing H5N1 subtype high-pathogenicity avian influenza virus-derived hemagglutinin or neuraminidase to generate mRNA-LNP vaccines against H5N1 influenza. Compared with conventional LNPs, mRNA-LNP$_{ssPalmO}$ induced comparable antigen-specific antibodies and better interferon-γ (IFN-γ)-producing T helper type 1 responses in mice. Both mRNA-LNP$_{ssPalmO}$ and conventional mRNA-LNPs conferred strong protection against homologous H5N1 virus challenge. In addition, mRNA-LNP$_{ssPalmO}$ showed better cross-protection against heterologous H5N1 virus challenge compared with conventional mRNA-LNPs. Furthermore, we observed that mRNA-LNP$_{ssPalmO}$ induced less-inflammatory responses (e.g., inflammatory cytokine production, vascular hyperpermeability) and fewer adverse reactions (e.g., weight loss, fever) compared with conventional mRNA-LNPs. These results suggest that mRNA-LNP$_{ssPalmO}$ would be a safe alternative to conventional vaccines to overcome mRNA-LNP vaccine hesitancy.

INTRODUCTION

Messenger RNA (mRNA) vaccines are promising vaccine modalities. For these vaccines to induce antigen-specific immune responses, the mRNA, which encodes the antigen, must reach target cells and produce sufficient antigen protein following immunization. Notably, lipid nanoparticles (LNPs) are key technological tools that deliver antigen-encoding mRNA. Antigen-specific immune responses, including antibody production and T cell activation, are strongly induced after immunization with mRNA-encapsulation LNPs

Received 31 May 2024; accepted 11 December 2024;
https://doi.org/10.1016/j.ymthe.2024.12.032.

[14]These authors contributed equally

Correspondence: Yasuo Yoshioka, Vaccine Creation Group, BIKEN Innovative Vaccine Research Alliance Laboratories, Research Institute for Microbial Diseases, Osaka University, 3-1 Yamadaoka, Suita, Osaka 565-0871, Japan
E-mail: y-yoshioka@biken.osaka-u.ac.jp

© 2024 The Author(s). Published by Elsevier Inc. on behalf of The American Society of Gene and Cell Therapy.
This is an open access article under the CC BY license (http://creativecommons.org/licenses/by/4.0/).

(mRNA-LNPs). In addition, mRNA is rapidly synthesized using *in vitro* transcription, and mRNA-LNPs can be rapidly manufactured on a large scale. Therefore, mRNA-encoded antigen information design provides vaccines against emerging threats.[1,2] These characteristics are advantageous in the event of a pandemic. Indeed, mRNA-LNP vaccines against coronavirus disease 2019 (COVID-19) have been developed at an unprecedented rate.[3,4] One study showed 61.3% vaccine effectiveness of mRNA-1273 vaccine against COVID-19 infection and 89.0% and 96.0% effectiveness against COVID-19 hospitalization and hospital death, respectively, under real-world conditions.[5] These findings suggest that the mRNA vaccine is effective for various COVID-19-related outcomes. Furthermore, mRNA-LNP-based vaccines against Zika, cytomegalovirus, influenza, and human respiratory syncytial viruses are undergoing clinical trials.[6]

However, adverse reactions, such as local and systemic reactions, caused by the mRNA-LNP vaccines against COVID-19 were frequently reported.[3,7–10] Goda et al. performed a study with 671 patients and reported that local reactions included injection site pain (frequency: 79%) and swelling (15%), and systemic responses included fever (4%), fatigue (13%), headache (10%), and muscle pain (34%) after the first dose of BNT162b2.[11] These reactions were more frequent after the second dose than the first, with fatigue increasing from 13% to 61%, headache from 10% to 45%, and fever from 4% to 41%. These adverse reactions result in hesitancy to receive COVID-19 vaccines, owing to fear of adverse reactions.[12,13] This aversion to vaccines could prevent vaccination with mRNA-LNPs against future human threats. Therefore, mRNA-LNP vaccine development with fewer adverse reactions would be required. Although the mechanism behind mRNA-LNP vaccine-mediated adverse reactions is not fully understood, inflammatory responses are one of the factors associated with adverse reactions.[14,15] Indeed, mRNA-LNP vaccines cause an increase in the levels of serum inflammatory cytokines in mice and humans[16–18] and systemic adverse reactions correlated with serum tumor necrosis factor α (TNF-α) levels.[19] Given these problems, an mRNA-LNP vaccine that maintains the necessary antigen-specific adaptive immune responses without inducing inflammatory responses would be ideal.

LNPs typically comprise a mixture of ionizable lipids, cholesterol, polyethylene glycol (PEG)-modified (PEGylated) lipids, and helper lipids.[20,21] Several reports have shown that LNPs have potential adjuvant activity to enhance adaptive immune responses, including antigen-specific antibody and T cell responses.[22–25] However, a recent report has indicated that LNPs lead to inflammatory reactions characterized by leukocytic infiltration and secretion of inflammatory cytokines and chemokines.[25–27] In particular, ionizable lipids mainly contribute to the inflammatory nature of LNPs.[26,28] Therefore, one approach to avoid adverse reactions is to modify ionizable lipids. We focused on disulfide (SS)-cleavable and pH-activated lipid-like materials (ssPalms) as ionizable lipids for LNPs.[28–30] Among a series of ssPalms, that with an oleic acid structure (ssPalmO) is a candidate for safe ionizable lipids due to its self-biodegradability and reduced toxicity.[31,32] Similar to conventional ionizable lipids, ssPalmO also has tertiary amines that are positively charged at low pH in the endosome and enable the exit of LNPs from the endosome to the cytosol.[30] Unlike conventional ionizable lipids, ssPalmO displays a disulfide bond and a phenyl ester. It is cleaved in an intracellular reducing environment to produce a thiol group, which subsequently attacks the phenyl ester linker group, leading to self-degradation. This spontaneous degradation occurs in the reducing environment of the cytoplasm.[31] These features of ssPalmO promote cytoplasmic delivery of loaded mRNA in LNPs. In addition, ssPalmO-based LNPs showed less hepatotoxicity than conventional LNPs after intravenous injection in rats, probably due to its biodegradability.[31] However, it remains unclear whether LNPs using ssPalmO ($LNP_{ssPalmO}$) can be used as an effective mRNA vaccine with reduced adverse reactions.

In recent years, wild bird and poultry infection with H5N1 high-pathogenicity avian influenza virus has spread worldwide, reaching not only Europe, Africa, and Asia but also North America and, for the first time, South America.[33,34] The infection of mammals feeding on these infected birds is also emerging.[35] Although, in general, avian influenza viruses likely do not infect humans, several human infection cases have been confirmed with the global spread of this virus in various animals. Moreover, as of April 1, 2024, 463 human deaths have been confirmed among 889 infected individuals since 2003, according to the World Health Organization (WHO).[36] Although efficient human-to-human H5N1 virus transmission has not been documented yet, this virus is considered a human pandemic threat, and the WHO is seeking an effective vaccine to meet the global need.

In this study, we demonstrate the potential of an mRNA-$LNP_{ssPalmO}$ vaccine to provide comparable protective immunity against H5N1 influenza viruses and fewer adverse reactions associated with reduced inflammatory responses compared with conventional mRNA-LNP vaccines. Our findings suggest that mRNA-$LNP_{ssPalmO}$ is a safe alternative to conventional vaccines for overcoming mRNA-LNP vaccine hesitancy.

RESULTS

$LNP_{ssPalmO}$ enhances antigen-specific antibody responses comparable to conventional LNPs

To evaluate the potential of $LNP_{ssPalmO}$ in mRNA vaccines, we compared the vaccine functions and adverse reactions (Figure 1A) of $LNP_{ssPalmO}$ and conventional LNPs using SM-102 ($LNP_{SM\text{-}102}$), a constituent ionizable lipid in the COVID-19 vaccine Spikevax.[37] The chemical structures of ssPalmO and SM-102 are shown in Figure 1B, and the lipid components in $LNP_{ssPalmO}$ and $LNP_{SM\text{-}102}$ are summarized in Figure 1C. Based on the dynamic light scattering and cryoelectron microscopy (cryo-EM) analyses, both $LNP_{ssPalmO}$ and $LNP_{SM\text{-}102}$ had a size of approximately 90–100 nm in size, a slightly negative charge (Figure 1C), and a spherical shape (Figure 1D). To minimize innate immune stimulation, we used mRNA modified with N1-methylpseudouridine, 5′ capped, and free of double-stranded RNA. This mRNA encoded the full-length antigens hemagglutinin (HA), and neuraminidase (NA) from the H5N1

A

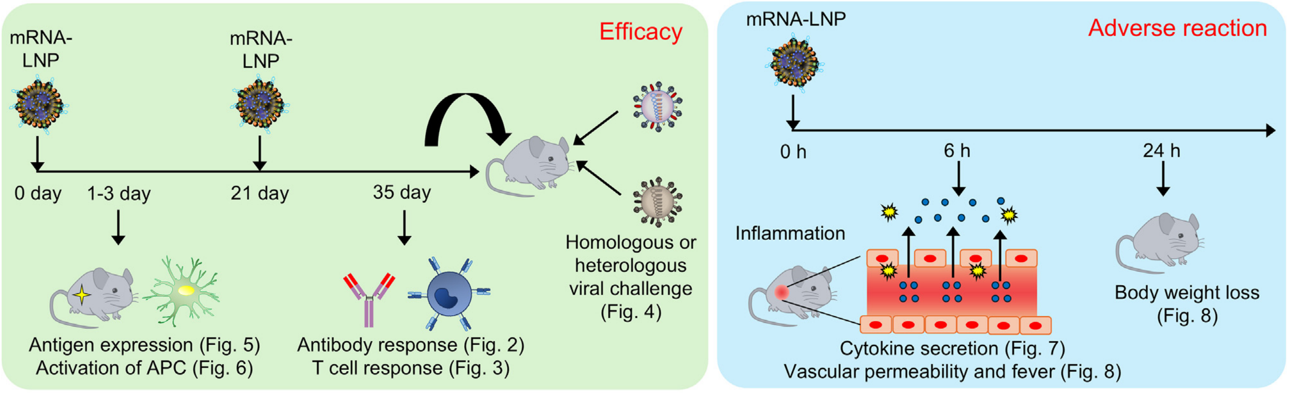

B

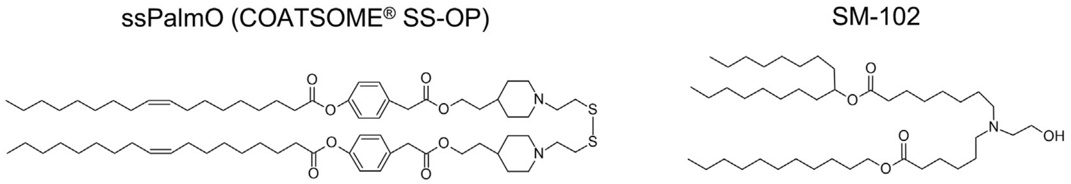

C

LNP Name	Lipid component	Zeta potential (mV)	Size (nm)
$LNP_{ssPalmO}$	ssPalmO, DOPC, Cholesterol, DMG-PEG2000	-5.3 ± 0.4	99.3 ± 5.5
$LNP_{SM\text{-}102}$	SM-102, DSPC, Cholesterol, DMG-PEG2000	-1.9 ± 1.3	92.5 ± 10.1

D

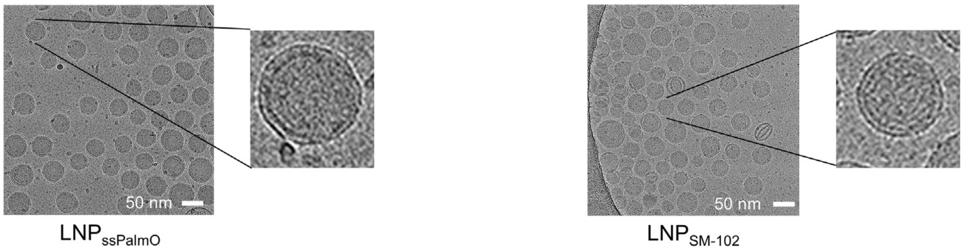

Figure 1. Overview of mRNA vaccine based on low-inflammatory lipid nanoparticle using ssPalmO

(A) Experimental schedule of animal experiments. (B) Chemical structure of ssPalmO and SM-102. (C) Lipid composition and characteristics of mRNA-$LNP_{ssPalmO}$ or $LNP_{SM\text{-}102}$. (D) Cryo-EM image of mRNA-LNP.

influenza A virus (strain: A/Viet Nam/1203/2004) encapsulated in LNPs (HA-LNP or NA-LNP, respectively). Notably, HA and NA are crucial for virus entry and release of virions and are the main targets of the influenza vaccine. The mice were subcutaneously immunized twice (prime and boost) with either HA-$LNP_{ssPalmO}$ or HA-$LNP_{SM\text{-}102}$. The mice received two subcutaneous immunizations with phosphate-buffered saline (PBS) as a control. Anti-HA immunoglobulin G1 (IgG1), IgG2b, and IgG2c, which are subclasses of IgG, were measured using an ELISA plate coated with recombinant HA (rHA). After booster immunization, the plasma levels of HA-specific IgG1, IgG2b, and IgG2c were found to be significantly higher in mice immunized with both HA-$LNP_{ssPalmO}$ and HA-$LNP_{SM\text{-}102}$ than in those immunized with PBS (Figure 2A). We observed certain differences in optical density (OD) in the HA-specific IgG1, IgG2b, and IgG2c plasma levels between mice immunized with HA-$LNP_{ssPalmO}$ and HA-$LNP_{SM\text{-}102}$ (Figure 2A). Additionally, we measured the endpoint titers of HA-specific IgG, which is the reciprocal log2 of the last dilution, with an absorbance >0.2. However, the

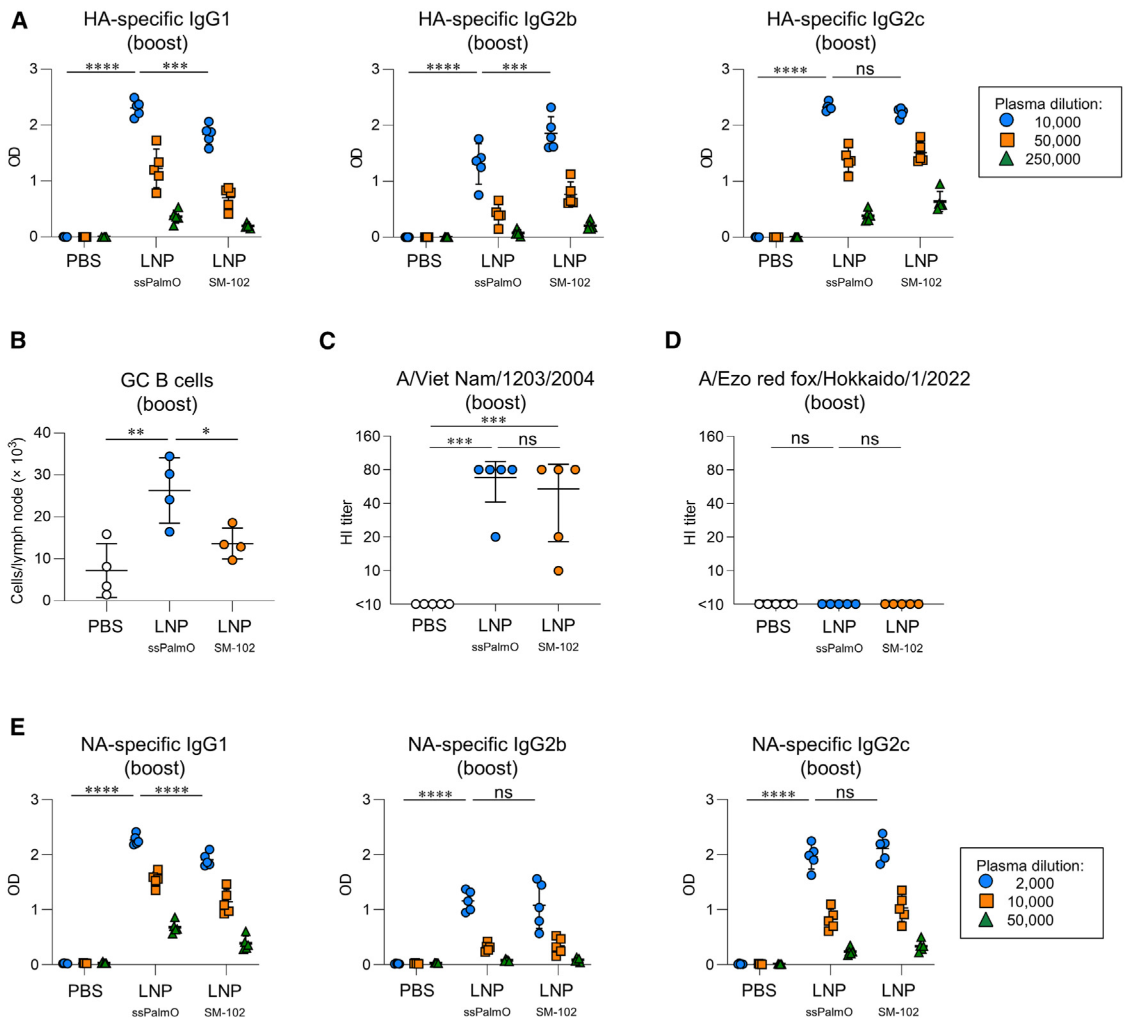

Figure 2. Antibody responses against HA and NA following subcutaneous immunization with mRNA-LNP

(A and B) Mice were subcutaneously immunized with HA-LNPs on day 0 (prime) and day 21 (boost). (A) The levels of HA-specific IgG1, IgG2b, and IgG2c in plasma on day 35 were evaluated with ELISA. These data are related to Figure S1. (B) The number of germinal center B cells in dLNs on day 35 was evaluated with flow cytometry. These data are related to Figure S2. (C and D) Hemagglutination inhibition titer of plasma on day 35 was assessed against H5N1 influenza A virus strain (C) A/Viet Nam/1203/2004 and (D) A/Ezo red fox/Hokkaido/1/2022. (E) Mice were subcutaneously immunized with NA-LNPs on day 0 (prime) and day 21 (boost). The levels of NA-specific IgG1, IgG2b, and IgG2c in plasma on day 35 were evaluated with ELISA. These data are related to Figures S5. (A–E) $n = 5$ per group. Data are means ± SDs. *$p < 0.05$; **$p < 0.01$; ***$p < 0.001$; ****$p < 0.0001$; Tukey's multiple-comparisons test; ns, not statistically significant. (A and E) Tukey's multiple-comparisons test was performed at a dilution of (A) 10,000 and (E) 2,000.

titer of HA-specific IgG1, IgG2b, and IgG2c were comparable between the HA-LNP$_{ssPalmO}$ and HA-LNP$_{SM-102}$ groups (Figure S1A). Similar to the results of the booster immunization, mice immunized with HA-LNP$_{ssPalmO}$ showed antigen-specific IgG levels comparable to those of HA-LNP$_{SM-102}$ after primary immunization (Figure S1B). In addition, we measured the germinal center (GC) B cell responses after booster immunization. These cells are the source of the high-affinity and class-switched antibodies required for protective immunity. HA-LNP$_{ssPalmO}$ induced a significantly higher number of GC B cells in draining lymph nodes (dLNs) than HA-LNP$_{SM-102}$ (Figures 2B and S2). Furthermore, we assessed the neutralization potential of induced IgG using hemagglutination inhibition (HI) assays, in which HI titers generally correlate with the neutralizing activity of the antibody. HI titers were assessed against the homologous H5N1 A/Viet Nam/1203/2004 strain, which is the same as the vaccine strain, or the heterologous H5N1 A/Ezo red fox/Hokkaido/1/2022 strain,[38] which is antigenically and originally different from the vaccine strain. The HI titer against the homologous strain was significantly higher in mice immunized with HA-LNP$_{ssPalmO}$ and HA-LNP$_{SM-102}$ than in those immunized with PBS (Figure 2C), whereas the HI titer was similar between HA-LNP$_{ssPalmO}$ and HA-LNP$_{SM-102}$. In contrast, HI titer against heterologous viruses was undetectable in all the

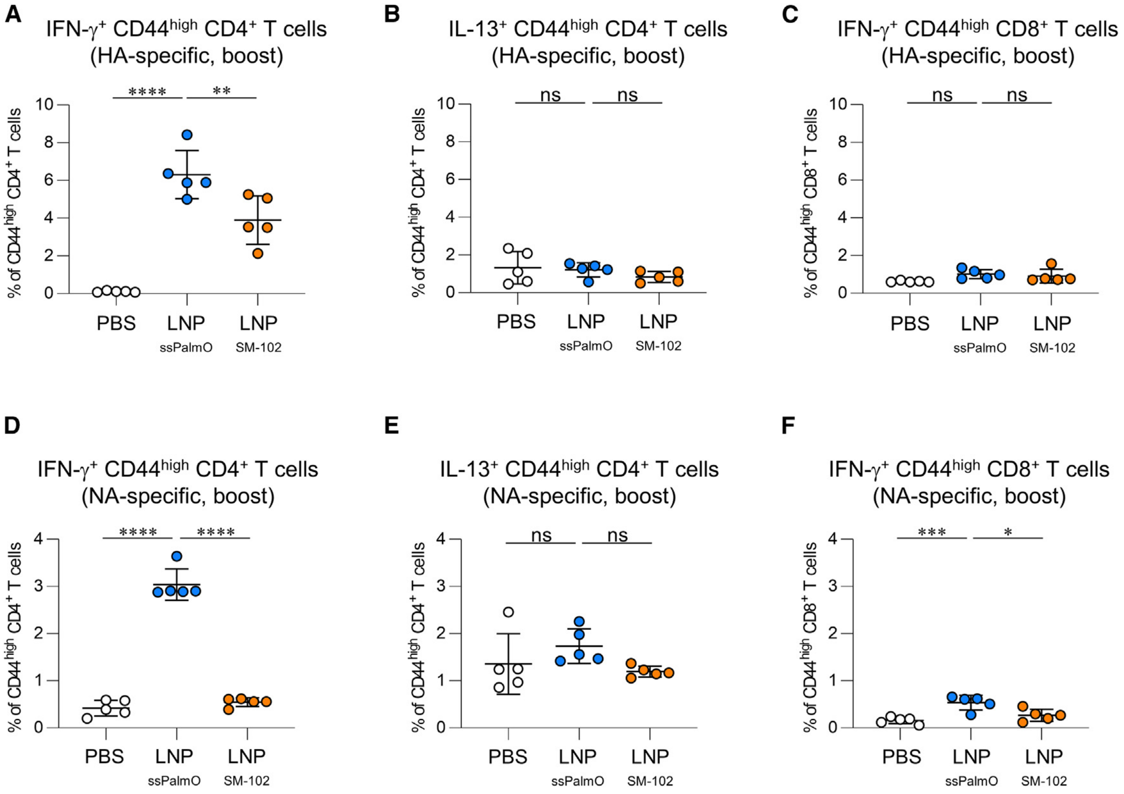

Figure 3. T cell responses against HA and NA following subcutaneous immunization with mRNA-LNP

Mice were subcutaneously immunized with (A–C) HA-LNP or (D–F) NA-LNP on days 0 (prime) and 21 (boost). On day 35, splenocytes from immunized mice were restimulated with (A–C) HA or (D–F) NA. Intracellular cytokine levels in (A and D) IFN-γ^+ CD44^{high} CD4$^+$ T cells, (B and E) IL-13$^+$ CD44^{high} CD4$^+$ T cells, and (C and F) IFN-γ^+ CD44^{high} CD8$^+$ T cells were evaluated. These data are related to Figures S6. (A–F) $n = 5$ per group. Data are means ± SDs. *$p < 0.05$; **$p < 0.01$; ***$p < 0.001$; ****$p < 0.0001$; Tukey's multiple-comparisons test; ns, not statistically significant.

groups (Figure 2D). In addition to subcutaneous immunization, intramuscular immunization, another primary route of vaccination, with HA-LNP$_{ssPalmO}$ and HA-LNP$_{SM\text{-}102}$ induced comparable levels of anti-HA IgG after booster immunization (Figure S3A). Similar to subcutaneous immunization, both HA-LNP$_{ssPalmO}$ and HA-LNP$_{SM\text{-}102}$ induced comparable HI titers against homologous viruses after intramuscular immunization, but not against heterologous viruses (Figures S3B and S3C). We compared the levels of antigen-specific IgG between mice immunized with HA-LNP$_{ssPalmO}$ and those immunized with rHA and alum, the most widely used adjuvants in humans, to confirm the potency of HA-LNP$_{ssPalmO}$. The HA-specific IgG2b and IgG2c levels were significantly higher in HA-LNP$_{ssPalmO}$-than in rHA plus alum-immunized mice in the 2,000-fold diluted sample, and the OD levels of HA-specific IgG2b and IgG2c in the 50,000-fold diluted sample in HA-LNP$_{ssPalmO}$ were equal to or higher than those of 2,000-fold diluted samples in rHA plus alum (Figure S4). The level of HA-specific IgG1 was slightly but significantly lower in mice immunized with HA-LNP$_{ssPalmO}$ than in those immunized with rHA plus alum (Figure S4). To evaluate the anti-NA responses, mice were subcutaneously immunized with NA-LNP$_{ssPalmO}$ and NA-LNP$_{SM\text{-}102}$. The level of NA-specific IgG1 in plasma was significantly higher in mice immunized with NA-LNP$_{ssPalmO}$ than in those immunized with NA-LNP$_{SM\text{-}102}$ (Figure 2E). Both NA-LNP$_{ssPalmO}$ and NA-LNP$_{SM\text{-}102}$ induced similar levels of NA-specific IgG2b and IgG2c (Figure 2E). Similar to the booster immunization results, mice immunized with NA-LNP$_{ssPalmO}$ showed antigen-specific IgG levels comparable to those of NA-LNP$_{SM\text{-}102}$ after primary immunization (Figure S5). Collectively, these results indicate that mRNA-LNP$_{ssPalmO}$ induced antigen-specific IgG levels comparable to conventional mRNA-LNPs, irrespective of the type of antigen and the route of administration.

LNP$_{ssPalmO}$ enhances IFN-γ-producing CD4$^+$ T cell response compared to conventional LNP

We measured antigen-specific T cell response in the spleen after subcutaneous booster immunization. Splenocytes from immunized mice were stimulated with HA, and the cytokine-producing HA-specific T cells were assessed using flow cytometry. HA-LNP$_{ssPalmO}$ induced a significantly higher interferon-γ (IFN-γ)-producing CD4$^+$ T cell (T helper type 1 [Th1] cells) percentage than PBS and HA-LNP$_{SM\text{-}102}$ (Figures 3A and S6). Furthermore, neither HA-LNP$_{ssPalmO}$ nor HA-LNP$_{SM\text{-}102}$ induced a detectable increase in

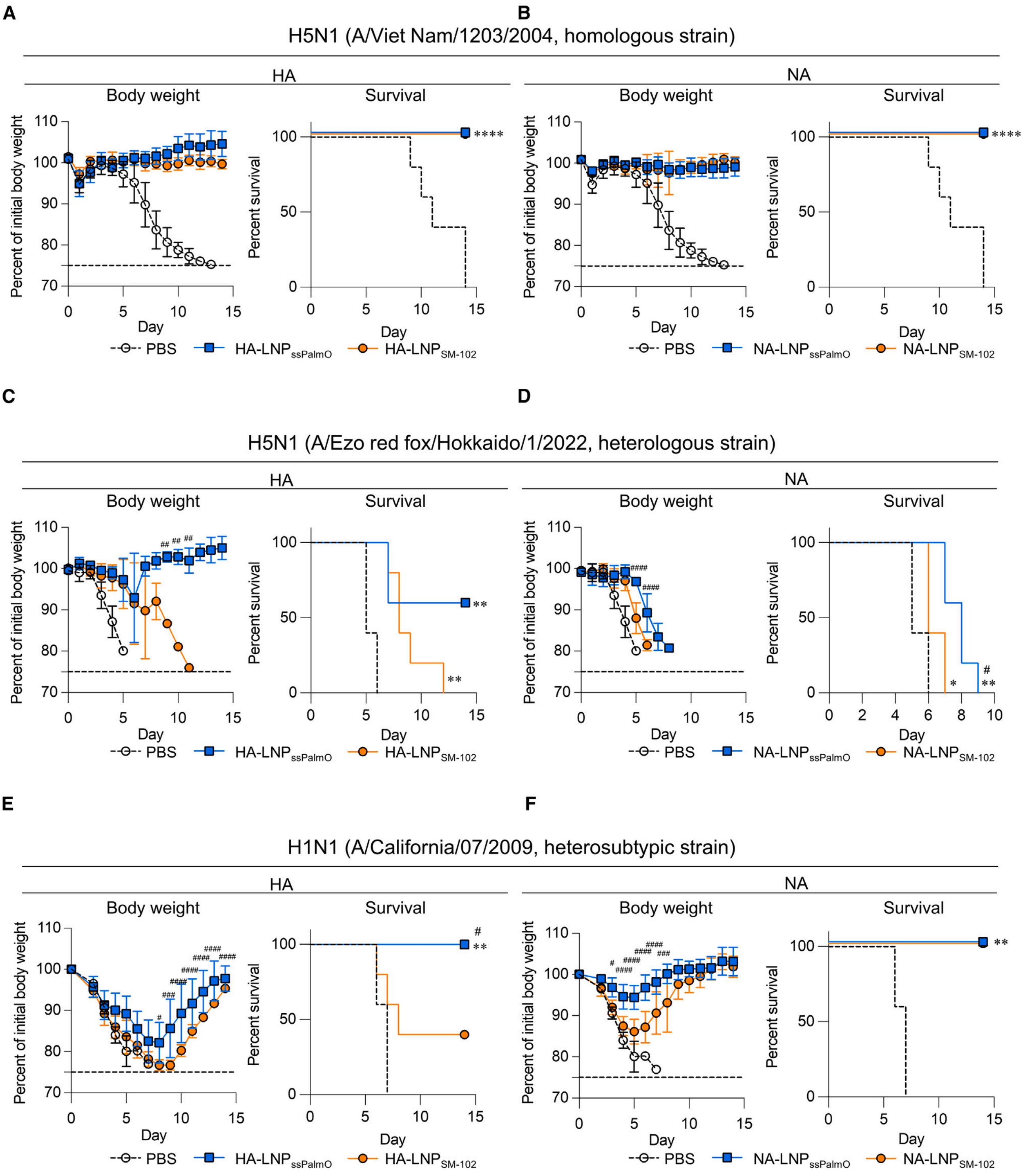

(legend on next page)

interleukin-13 (IL-13)-producing CD4$^+$ T cells (Th2 cells) and IFN-γ-producing CD8$^+$ T cells (killer T cells for the infected cells), compared with PBS (Figures 3B and 3C). We also confirmed that HA-LNP$_{ssPalmO}$ induced significantly more IFN-γ-producing CD4$^+$ T cells than rHA plus alum (Figures S7A–S7C). NA-LNP$_{ssPalmO}$ induced a significantly higher IFN-γ-producing CD4$^+$ and CD8$^+$ T cell percentage compared to PBS and NA-LNP$_{SM\text{-}102}$ (Figures 3D and 3F), although NA-LNP$_{ssPalmO}$ and NA-LNP$_{SM\text{-}102}$ did not induce IL-13-producing CD4$^+$ T cells like PBS (Figure 3E). These results suggest that LNP$_{ssPalmO}$ induces more IFN-γ-producing T cells compared to conventional mRNA-LNP vaccine.

LNP$_{ssPalmO}$ confers stronger protection against heterologous influenza virus challenges than conventional LNP

We evaluated whether immunization with mRNA-LNP$_{ssPalmO}$ exerted a protective effect against the influenza virus challenge. The mice were intranasally challenged with the homologous H5N1 A/Viet Nam/1203/2004 strain, which is a homologous strain of the immunized HA, after booster immunization through the subcutaneous route. Subsequently, changes in body weight and survival were observed. The body weights of PBS-treated mice decreased after the challenge, and all those mice died within 15 days (Figure 4A). HA-LNP$_{ssPalmO}$, HA-LNP$_{SM\text{-}102}$, NA-LNP$_{ssPalmO}$, and NA-LNP$_{SM\text{-}102}$ completely protected against body weight loss and death after challenge (Figures 4A and 4B). Furthermore, to evaluate cross-protection against viruses that differ from immunized antigens, the immunized mice were challenged with the heterologous H5N1 A/Ezo red fox/Hokkaido/1/2022 strain. The body weight of PBS-treated mice decreased after challenge, and all mice died within 6 days (Figures 4C and S8A). Mice immunized with HA-LNP$_{SM\text{-}102}$ also exhibited severe weight loss, and all the mice died within 12 days, although the survival period of the mice was prolonged compared to that of PBS-treated mice (Figures 4C and S8A). The mice immunized with HA-LNP$_{ssPalmO}$ showed a milder weight loss than mice immunized with HA-LNP$_{SM\text{-}102}$, with a survival rate of 60% (Figures 4C and S8A). In contrast, the body weights of mice immunized with NA-LNP$_{ssPalmO}$ and NA-LNP$_{SM\text{-}102}$ decreased after the heterologous viral challenge (Figures 4D and S8B). Moreover, all mice immunized with NA-LNP$_{ssPalmO}$ and NA-LNP$_{SM\text{-}102}$ died within 10 days, although immunization with NA-LNP$_{ssPalmO}$ significantly prolonged survival compared to mice immunized with PBS and suppressed body weight loss compared to NA-LNP$_{SM\text{-}102}$ mice (Figures 4D and S8B). Furthermore, we challenged the immunized mice with heterosubtypic seasonal H1N1 A/California/07/2009 strain, which differs from the H5N1 strain. The body weights of PBS-treated mice and mice immunized with HA-LNP$_{ssPalmO}$ and HA-LNP$_{SM\text{-}102}$ decreased after the challenge; however, the recovery of body weight was faster in the mice immunized with HA-LNP$_{ssPalmO}$ than in those immunized with HA-LNP$_{SM\text{-}102}$ (Figures 4E and S8C). Furthermore, the survival rates of mice immunized with HA-LNP$_{ssPalmO}$ and HA-LNP$_{SM\text{-}102}$ were 100% and 40%, respectively, with a significant difference between the groups, whereas all mice treated with PBS died within 15 days (Figures 4E and S8C). Additionally, NA-LNP$_{ssPalmO}$ significantly suppressed weight loss compared to NA-LNP$_{SM\text{-}102}$, and both groups showed no mortality (Figures 4F and S8D). Collectively, these results suggest that mRNA-LNP$_{ssPalmO}$ confers broader cross-protection than does mRNA-LNP$_{SM\text{-}102}$.

We further evaluated the contribution of CD4$^+$ T cells to a cross-protective effect of HA-LNP$_{ssPalmO}$ against heterologous viral challenge. Mice were immunized with HA-LNP$_{ssPalmO}$. After the depletion of CD4$^+$ T cells by anti-CD4 antibody, the immunized mice were challenged with the heterologous H5N1 A/Ezo red fox/Hokkaido/1/2022 strain. Mice treated with an isotype control antibody showed milder weight loss than those immunized with PBS, achieving a survival rate of 60% (Figure S9). In mice treated with an anti-CD4 antibody, body weight loss was exacerbated and all mice died (Figure S9). These results suggest that CD4$^+$ T cells are associated with cross-protection against heterogeneous virus challenges in mice immunized with HA-LNP$_{ssPalmO}$.

LNP$_{ssPalmO}$- and conventional LNP-mediated luciferase expressions were similar both at the injection site and in dLNs

We measured the kinetics of antigen expression using luciferase (Luc)-encoding mRNA to compare the immunostimulatory properties of LNP$_{ssPalmO}$ and LNP$_{SM\text{-}102}$. Following intramuscular injection of Luc-LNPs, luminescence was measured using an *in vivo* imaging system. Strong luminescence was observed at the injection site 6 and 24 h after the injection of Luc-LNP$_{ssPalmO}$ and Luc-LNP$_{SM\text{-}102}$, whereas weak luminescence was detected in the abdomen, probably derived from the liver (Figure 5A). Luminescence from the injection site gradually decreased in 48 and 72 h and that from the abdomen disappeared in 48 h (Figure 5A). There was no significant difference in luminescence from the injection site between Luc-LNP$_{ssPalmO}$ and Luc-LNP$_{SM\text{-}102}$ (Figure 5B). Furthermore, luminescence was measured after repeated injections of mRNA-LNPs at 3-week intervals, and the luminescence profile at the second dose was similar to that at the first dose (Figures S10A and S10B). Luc activity was measured in the muscle, dLN, liver, and spleen homogenates. Luc activity increased in the muscle, dLN, and liver following the injection of both LNPs compared to PBS (Figures 5C–5F). In particular, luminescence activity was the highest in the muscle, and the level was similar between Luc-LNP$_{ssPalmO}$ and Luc-LNP$_{SM\text{-}102}$ after the first dose

Figure 4. Protective effect of mRNA-LNP vaccines against H5N1 influenza A virus challenge

Mice were subcutaneously immunized with (A, C, and E) HA-LNPs or (B, D, and F) NA-LNPs on days 0 (prime) and 21 (boost). On day 35, the mice were intranasally challenged with (A and B) H5N1 A/Viet Nam/1203/2004 strain, (C and D) H5N1 A/Ezo red fox/Hokkaido/1/2022 strain, or (E and F) H1N1 A/California/07/2009 strain. Body weight changes and survival rates were monitored for 14 days after virus challenge. Data for the PBS-treated groups are the same. These data are related to Figures S8. (A–F) $n = 5$ per group. Data are means ± SDs. Body weight: $^{\#}p < 0.05$; $^{\#\#}p < 0.01$; $^{\#\#\#}p < 0.001$; $^{\#\#\#\#}p < 0.0001$; Tukey's multiple comparison test compared to HA-LNP$_{SM\text{-}102}$ or NA-LNP$_{SM\text{-}102}$. Survival: $^{*}p < 0.05$; $^{**}p < 0.01$; $^{****}p < 0.0001$; log rank test compared with PBS; $^{\#}p < 0.05$, log rank test compared with HA-LNP$_{SM\text{-}102}$ or NA-LNP$_{SM\text{-}102}$.

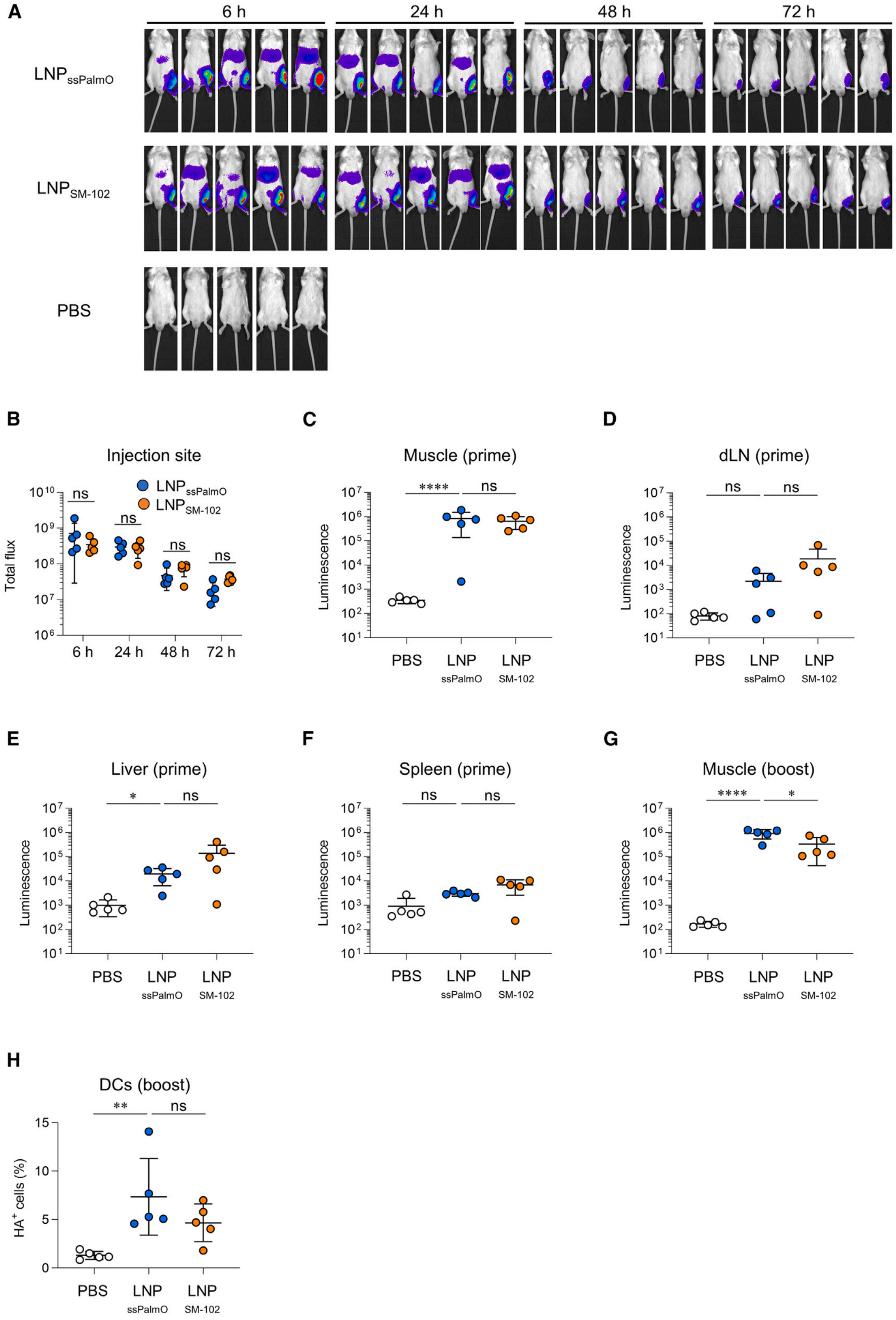

(legend on next page)

(Figures 5C–5F). Following the second injection of mRNA-LNP, the luminescence activity of the muscle in Luc-$LNP_{ssPalmO}$-treated mice was significantly higher than that in Luc-$LNP_{SM\text{-}102}$-treated mice (Figure 5G). The expression of HA in dendritic cells (DCs) in the muscle was also evaluated using flow cytometry following booster immunization with HA-LNP (Figure S11). The percentage of HA^+ DCs in HA-$LNP_{ssPalmO}$ was similar to that in mice immunized with HA-$LNP_{SM\text{-}102}$ (Figure 5H). These results suggest that $LNP_{ssPalmO}$ can express antigens to a similar extent as $LNP_{SM\text{-}102}$.

$LNP_{ssPalmO}$ induces milder antigen-presenting cell activation than conventional LNP

To assess the adjuvancity of LNPs, we examined the expression of the activation marker CD86, which is a co-stimulatory molecule for T cell activation, on antigen-presenting cells (APCs), such as plasmacytoid DCs (pDCs), conventional DCs (cDCs), migratory DCs (mDCs), B cells, and macrophages in the dLNs 24 h after primary immunization via the subcutaneous route. HA-$LNP_{ssPalmO}$ induced a significantly enhanced expression of CD86 in pDCs, mDCs, and macrophages compared to PBS, whereas HA-$LNP_{SM\text{-}102}$ induced significant expression in all APCs (Figures 6 and S12). The levels of CD86 in cDCs, mDCs, B cells, and macrophages were lower for HA-$LNP_{ssPalmO}$ than for HA-$LNP_{SM\text{-}102}$ (Figure 6). These results suggest that $LNP_{ssPalmO}$ can activate APCs to a similar or lesser extent than $LNP_{SM\text{-}102}$.

Inflammatory responses in dLNs are not related to higher efficacy of $LNP_{ssPalmO}$

Further studies were performed to investigate the immunostimulatory properties of $LNP_{ssPalmO}$ and $LNP_{SM\text{-}102}$. It is known that DCs produce IL-12, which regulates IFN-γ-producing $CD4^+$ T cell responses. Thus, we measured IL-12 in dLNs after subcutaneous immunization with HA-$LNP_{SM\text{-}102}$ or HA-$LNP_{ssPalmO}$. The difference in IL-12 levels in the dLNs was not observed between mice immunized with HA-$LNP_{SM\text{-}102}$ and HA-$LNP_{ssPalmO}$ (Figure S13A). In addition, we measured the level of IL-12 at the injection site after intramuscular immunization. Similar to the level of IL-12 in dLNs, we did not find any difference in IL-12 levels between these LNPs (Figure S13B). These results suggest that IL-12 in dLNs and at the injection site might not contribute to increasing IFN-γ-producing $CD4^+$ T cells in mice immunized with $LNP_{ssPalmO}$.

Furthermore, we performed RNA sequencing (RNA-seq) analysis of the dLNs after vaccination with LNPs to compare the immunostimulatory properties of $LNP_{ssPalmO}$ and $LNP_{SM\text{-}102}$. Notably, principal-component analysis and heatmap analysis revealed distinctly different gene expression patterns in mice immunized with HA-$LNP_{SM\text{-}102}$ compared with HA-$LNP_{ssPalmO}$ and PBS (Figures S14A and S14B). The expression shown in the mice immunized with HA-$LNP_{ssPalmO}$ was closer to that shown in the mice immunized with PBS than the mice immunized with HA-$LNP_{SM\text{-}102}$ (Figure S14A). The mice immunized with HA-$LNP_{SM\text{-}102}$ showed upregulation of 831 genes compared with the mice immunized with HA-$LNP_{ssPalmO}$ (Figure S14C). Specifically, HA-$LNP_{SM\text{-}102}$ significantly increased the expression of *Ifna*, *Ifnb*, *Il6*, and *Cxcl10* genes and plasma cytokine levels compared with the HA-$LNP_{ssPalmO}$. In addition, the differentially expressed genes (DEGs) in mice immunized with HA-$LNP_{SM\text{-}102}$ were significantly enriched with IFN and inflammatory responses and TNF-α signaling via nuclear factor-κB compared with those in mice immunized with HA-$LNP_{ssPalmO}$ (Figure S14D). Therefore, the inflammatory pathway of dLNs may differ in $LNP_{ssPalmO}$ compared with $LNP_{SM\text{-}102}$. Furthermore, we performed RNA-seq analysis of DCs isolated from dLNs after vaccination with LNPs to confirm the contribution of DCs to inflammatory and innate immune responses induced by LNPs. The DCs from the mice immunized with HA-$LNP_{SM\text{-}102}$ showed distinctly different gene expression patterns compared with those immunized with HA-$LNP_{ssPalmO}$ and PBS, similar to the results of the dLNs (Figure S15). The DEGs in mice immunized with HA-$LNP_{SM\text{-}102}$ were enriched with IFN responses and inflammatory responses compared with those in mice immunized with HA-$LNP_{ssPalmO}$. Collectively, $LNP_{ssPalmO}$ induced reduced inflammatory responses; however, it did induce strong immune responses against H5N1.

$LNP_{ssPalmO}$ induces reduced inflammatory responses and fewer adverse reactions compared to conventional LNPs

The plasma levels of inflammatory cytokines were determined 6 h after the primary and booster immunizations to compare the inflammatory responses to LNPs. This was performed to indicate common adverse reactions. We found that there were no significant differences in the levels of IFN-α, IFN-β, IFN-γ, C-C motif ligand 2 (CCL2), C-X-C motif chemokine ligand 10 (CXCL10), and IL-6 in the plasma between mice immunized with PBS and HA-$LNP_{ssPalmO}$, although the levels of these cytokines in HA-$LNP_{SM\text{-}102}$-immunized mice were significantly higher than those in PBS-treated mice (Figures 7A–7F). The plasma levels of these cytokines were significantly lower in mice immunized with HA-$LNP_{ssPalmO}$ than in those immunized with HA-$LNP_{SM\text{-}102}$ after both primary and booster immunization (Figures 7A–7F). CXCL1, TNF-α, IL-12p70, CCL5, IL-1β, granulocyte-macrophage colony-stimulating factor (GM-CSF), and IL-10 levels did not increase in mice immunized with HA-$LNP_{ssPalmO}$ or HA-$LNP_{SM\text{-}102}$ compared with PBS-treated mice

Figure 5. Antigen expression following intramuscular immunization with mRNA-LNP

(A–F) Mice were intramuscularly injected with luciferase (Luc)-LNP. (A and B) At indicated time points, luminescence was measured using *in vivo* imaging system. (A) Whole image at 6, 24, 48, and 72 h. (B) Total flux at the injection site of whole images was quantitated. (C–F) At 6 h, (C) muscle, (D) dLN, (E) liver, and (F) spleen were collected from the mice. Luc activity in each tissue homogenate was measured. (G) Mice were intramuscularly injected with Luc-LNPs repeatedly at 3-week intervals. At 6 h after the second dose, Luc activity in the muscle homogenate was measured. (H) Mice were intramuscularly immunized with HA-LNPs on day 0 (prime) and day 21 (boost). At 24 h post-booster immunization, muscles were collected, and the expression of HA in DCs was measured using flow cytometry. These data are related to Figures S11. (A–H) $n = 5$ per group. Data are means ± SDs. $^*p < 0.05$; $^{**}p < 0.01$; $^{****}p < 0.0001$; Tukey's multiple-comparisons test; ns, not statistically significant.

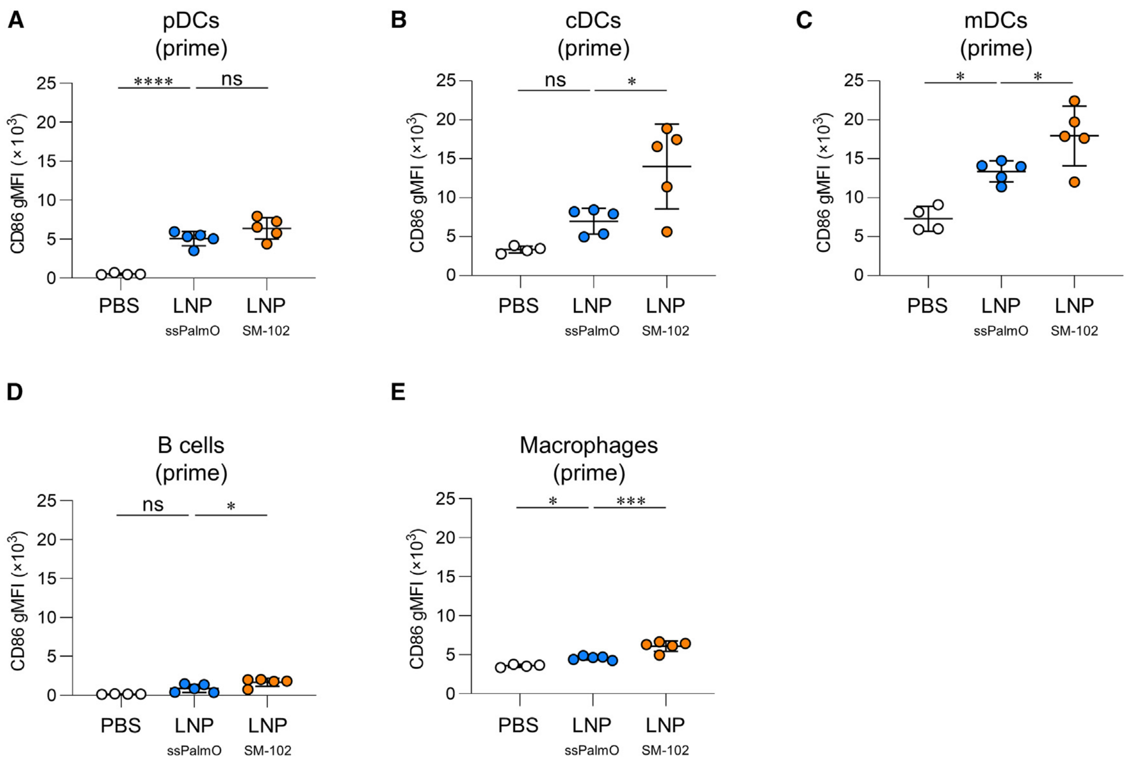

Figure 6. Activation of the antigen-presenting cells following subcutaneous immunization with mRNA-LNP

Mice were subcutaneously immunized with HA-LNP. After 24 h, the levels of CD86 in (A) pDCs, (B) cDCs, (C) mDCs, (D) B cells, and (E) macrophages in the dLNs were evaluated by flow cytometry. These data are related to Figures S12. (A–E) $n = 5$ per group. Data are means ± SDs. *$p < 0.05$; ***$p < 0.001$; ****$p < 0.0001$; Tukey's multiple-comparisons test; ns, not statistically significant.

(Figure S16). Similar to the results of subcutaneous immunization, intramuscular immunization with HA-LNP$_{\mathrm{ssPalmO}}$ induced lower levels of IFN-α, IFN-β, IFN-γ, CCL2, and CXCL10 in the plasma than did HA-LNP$_{\mathrm{SM\text{-}102}}$ (Figure S17). These results suggest that LNP$_{\mathrm{ssPalmO}}$ reduced the production of inflammatory cytokines compared with LNP$_{\mathrm{SM\text{-}102}}$.

The inflammatory cytokines in heart tissues were determined on days 1 and 2 after immunizations to compare myocarditis reactions and rare adverse reactions induced by mRNA-LNP. We found that the level of most cytokines (*Ifna*, *Tnfa*, *Il1b*, *Il6*, and *Ccl2*) did not significantly differ among the mice immunized with PBS, LNP$_{\mathrm{ssPalmO}}$, and LNP$_{\mathrm{SM\text{-}102}}$ (Figure S18). In addition, we observed elevated levels of *Infb* in mice immunized with LNP$_{\mathrm{SM\text{-}102}}$ at day 1 after immunization, and the level of *Infb* was lower in mice immunized with LNP$_{\mathrm{ssPalmO}}$ compared with mice immunized with LNP$_{\mathrm{SM\text{-}102}}$. These results indicate that LNP$_{\mathrm{ssPalmO}}$ might reduce the possibility of myocarditis compared with LNP$_{\mathrm{SM\text{-}102}}$.

We examined vascular permeability at the injection site as an indicator of local inflammation after intramuscular immunization of HA-LNP$_{\mathrm{ssPalmO}}$ or HA-LNP$_{\mathrm{SM\text{-}102}}$. Evans blue dye, which extravasates from vessels and accumulates at the inflammatory site by binding to albumin, was injected after immunization with HA-LNPs. The leakage of Evans blue dye at the injection site was significantly higher in mice treated with HA-LNP$_{\mathrm{SM\text{-}102}}$ than the PBS-treated mice; however, it was not observed in mice treated with HA-LNP$_{\mathrm{ssPalmO}}$ (Figure 8A). We measured the changes in body weight as indicators of adverse reactions following intramuscular immunization with high doses (20 μg/mouse) of HA-LNPs. Body weight loss was significantly milder in mice immunized with HA-LNP$_{\mathrm{ssPalmO}}$ than in those immunized with HA-LNP$_{\mathrm{SM\text{-}102}}$ 1 day after prime immunization (Figure 8B). Furthermore, the rectal temperatures of mice were determined following prime and booster immunization with HA-LNPs (5 μg/mouse). We observed rectal temperatures of mice immunized with HA-LNP$_{\mathrm{ssPalmO}}$ similar to those of the PBS-treated mice, whereas HA-LNP$_{\mathrm{SM\text{-}102}}$ caused a significant increase in rectal temperature after the primary (Figure 8C) and booster (Figure 8D) immunizations. These results suggest that LNP$_{\mathrm{ssPalmO}}$ attenuates severe inflammatory responses and adverse reactions compared with LNP$_{\mathrm{SM\text{-}102}}$.

ssPalmO rather than helper lipid contributes to the reduced inflammatory properties of LNP$_{\mathrm{ssPalmO}}$

We used 1,2-dioleoyl-*sn*-glycero-3-phosphocholine (DOPC) as a helper lipid in LNP$_{\mathrm{ssPalmO}}$ and 1,2-distearoyl-*sn*-glycero-3-phosphocholine (DSPC) as a helper lipid in LNP$_{\mathrm{SM\text{-}102}}$ (Figure 1C). To

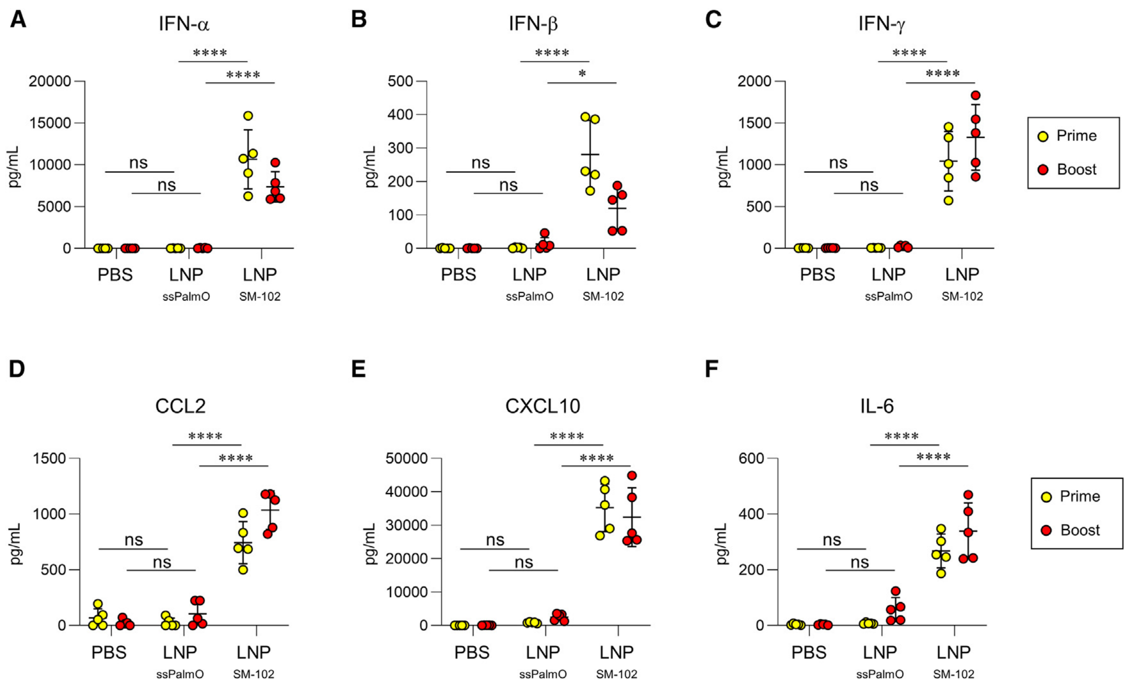

Figure 7. Attenuation of inflammatory cytokine production following subcutaneous immunization with mRNA-LNP$_{ssPalmO}$

Mice were subcutaneously immunized with HA-LNPs on days 0 (prime) and 21 (boost). At 6 h after primary and booster immunizations, the levels of (A) IFN-α, (B) IFN-β, (C) IFN-γ, (D) CCL2, (E) CXCL10, and (F) IL-6 in the plasma were measured. (A–F) n = 5 per group. Data are means ± SDs. *p < 0.05; ****p < 0.0001; Tukey's multiple-comparisons test; ns, not statistically significant.

evaluate the effect of helper lipid differences, DOPC in LNP$_{ssPalmO}$ was replaced with the DSPC used in LNP$_{SM-102}$. We found that the levels of IFN-α, IFN-β, IFN-γ, CCL2, CXCL10, and IL-6 in HA-LNP$_{ssPalmO}$ with DSPC were significantly lower than those in HA-LNP$_{SM-102}$ (Figures S19A–S19F), whereas the levels of IFN-γ and CXCL10 in HA-LNP$_{ssPalmO}$ with DSPC were significantly higher than those in the original formulation of HA-LNP$_{ssPalmO}$ with DOPC (Figures S19C and S19E). In addition, the levels of HA-specific IgG1, IgG2b, and IgG2c in the plasma were comparable between mice immunized with the original formulation of LNP$_{ssPalmO}$ with DOPC and LNP$_{ssPalmO}$ with DSPC (Figure S19G). These results suggest that ssPalmO, rather than DOPC, in LNP$_{ssPalmO}$ contributes to a lower inflammatory response.

DISCUSSION

Adverse reactions should be minimized to further accelerate the clinical translation of LNP-based therapeutics, including mRNA-LNP vaccines. We demonstrated that mRNA-LNP$_{ssPalmO}$ induced reduced inflammatory responses and adverse reactions compared to conventional mRNA-LNP$_{SM-102}$. Our results suggest the promising potential of mRNA-LNP$_{ssPalmO}$ in overcoming the mRNA-LNP vaccine-associated hesitancy.

As several ionizable or cationic lipids possess cytotoxic effects, they frequently cause cell death, danger signal release, and inflammatory reactions.[39] Therefore, the biodegradability of ionizable lipids is important to avoid longer exposure to cells and subsequent inflammatory reactions. SM-102 exhibits an ester linker between the head and tail groups in its structure, degraded by esterase[40] and, presumably, further β-oxidation,[41] after the injection into the body. However, despite the biodegradability of SM-102, LNP$_{SM-102}$ causes inflammatory reactions. Indeed, LNPs formulated with SM-102 are potent activators of the inflammasome pathway, indicated by the robust release of IL-1β and IL-6.[25] In contrast, LNP$_{ssPalmO}$ induced fewer inflammatory reactions, probably because ssPalmO has self-degrading properties in addition to esterase-dependent degradation. ssPalmO has a disulfide bond that is cleaved rapidly in a reducing environment such as cytoplasm.[31] The resultant thiol group of ssPalmO can attack phenyl ester linkers in self-structure and be degraded into oleic acid and hydrophilic amine. This self-degradable property of ssPalmO might contribute to its rapid biodegradation, mild toxicity, and inflammation. Furthermore, some reports have indicated that oleic acid functions as an immunosuppressant, and oleic acid treatment decreases inflammatory cytokines.[42,43] For example, it has been reported that oleic acid-loaded nanoparticles suppress elastase, superoxide anion, and cytokines released from neutrophils.[44] Therefore, oleic acid derived from ssPalmO might contribute to the lower inflammatory properties of LNP$_{ssPalmO}$.

Helper lipids play a crucial role in the stabilization of LNP and mRNA delivery efficiency in collaboration with ionizable lipids.[45] Therefore, the replacement of helper lipids in LNPs affects the

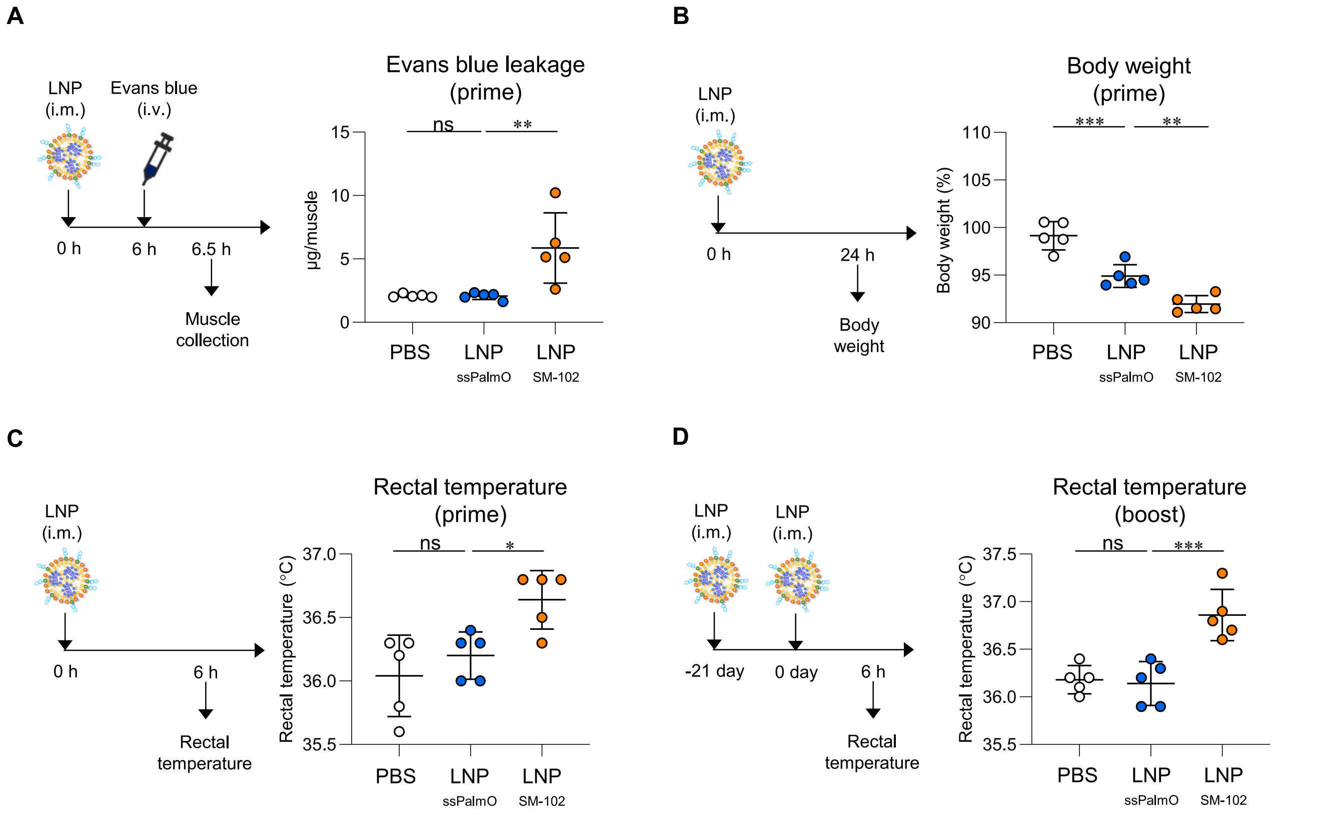

Figure 8. Attenuation of adverse reaction following intramuscular immunization with mRNA-LNP$_{ssPalmO}$

(A) The leakage of Evans blue at injection site was measured 6 h after intramuscular immunization with HA-LNP. (B) Body weight was measured before and 24 h after intramuscular immunization of HA-LNP. (C and D) Rectal temperature was measured 6 h after (C) primary and (D) booster immunization of HA-LNP. (A–D) n = 5 per group. Data are means ± SDs. **$p < 0.01$; ***$p < 0.001$; Tukey's multiple-comparisons test; ns, not statistically significant.

efficacy of mRNA delivery, protein expression, and subsequent immune responses. DSPC-containing LNPs tightly assemble and hinder the release of nucleic acids from the endosome into the cytoplasm, thus suppressing protein expression, while DOPC-containing LNPs have moderate fluidity, which enhances the release of nucleic acids, resulting in increased protein expression.[46,47] We used DSPC for LNP$_{SM\text{-}102}$ because DSPC is used as a vaccine against COVID-19, whereas DOPC was used for LNP$_{ssPalmO}$ in accordance with the above reports. Our results showed that both HA-LNP$_{ssPalmO}$ with DOPC and HA-LNP$_{ssPalmO}$ with DSPC induce comparable antibody responses. In contrast, the levels of some inflammatory cytokines in HA-LNP$_{ssPalmO}$ with DSPC were partially increased compared with those in HA-LNP$_{ssPalmO}$ with DOPC, indicating that DOPC is less inflammatory than DSPC. However, cytokine production in HA-LNP$_{ssPalmO}$ with DSPC was significantly lower than that in HA-LNP$_{SM\text{-}102}$. Therefore, replacing SM-102 with ssPalmO had a dominant effect on the reduced inflammatory properties of LNP$_{ssPalmO}$.

Infection- or inflammation-mediated fever is initiated by the recognition of danger signals by the immune cells.[48] Inflammatory cytokines produced by immune cells, such as IL-6, IL-1, and TNF-α, cause fever through cyclooxygenase-2-mediated prostaglandin E2 production.[48] Moreover, these cytokines reportedly increase vascular permeability and body weight loss.[49,50] Therefore, the reduced adverse reactions (e.g., enhanced vascular permeability, body weight loss, fever) of LNP$_{ssPalmO}$ might result from reduced inflammatory cytokine production (e.g., that of IL-6) in LNP$_{ssPalmO}$.

mRNA vaccines sometimes cause rare adverse events such as cardiac reactions, anaphylaxis, arthralgias, and myalgias, potentially due to the pro-inflammatory nature of the mRNA vaccine. Hence, prevention of these adverse events is required to develop safe mRNA vaccines. Some researchers evaluated cardiac reactions following immunization with mRNA-LNPs by measuring cardiac cytokines in mice.[51,52] For example, Li et al. highlighted that inflammatory cytokines such as IFN-α, IFN-β, and IL-6 were transiently upregulated in heart tissues after immunizing mice with BNT162b2 in mice.[52] We demonstrated that the level of IFN-β in heart homogenates was lower in mice immunized with spike-LNP$_{ssPalmO}$ than with spike-LNP$_{SM\text{-}102}$, whereas other cytokines were not increased in any of the groups assessed. In contrast to previous reports, we did not observe an increase in cytokines other than IFN-β in our results, which we attribute to differences in experimental conditions,

including the LNPs used. Furthermore, $LNP_{ssPalmO}$ could reduce inflammatory cytokines, thereby reducing the burden of cardiac reactions.

Moreover, the pro-inflammatory nature of the mRNA vaccine is reduced by modifying the structure of mRNA and LNP. Replacing uridine with pseudouridine makes mRNA non-immunogenic by reducing its inflammatory nature through inhibiting Toll-like receptor signaling.[53] In addition, removing double-stranded RNA and inserting 5′ cap analogs reduce recognition by the innate immune system.[54] These mRNA modification technologies contribute to the development of the COVID-19 vaccine. Furthermore, ionizable lipids in LNPs have been reported to have a pro-inflammatory role in the mRNA vaccine.[26] Therefore, modifying ionizable lipids is one approach to avoid adverse reactions. Our study findings revealed that replacing $LNP_{SM\text{-}102}$ with $LNP_{ssPalmO}$ produced comparable immune responses with reduced adverse reactions, suggesting that inflammatory responses are not always accompanied by efficacy and adverse reactions. In contrast, Takano et al. reported that some inflammatory cytokines induced by vaccines are correlated with neutralizing antibody titers and systemic adverse reactions.[55] Thus, it is important to consider the cytokine balance in the induction of neutralizing antibodies and reactogenicity. In addition, how $LNP_{ssPalmO}$ elicited strong immune responses with reduced adverse reactions remains unclear; however, further research on $LNP_{ssPalmO}$ could clarify the relationship between inflammation and each response.

Consistent with our findings, recent reports have shown that optimization of the LNP formulation is a useful approach for reducing the inflammatory properties of LNP. Xu et al. developed LNPs with lower inflammatory potential than traditional ionizable lipids such as MC3, using 1,2-diesters-derived ionizable lipids, which were identified using a novel library of 248 ionizable lipids.[56] In addition, LNPs with lipid-modified poly(guanidine thioctic acid) polymer has reduced inflammatory responses with the ability to scavenge reactive oxygen species.[57] Therefore, the selection of ionizable lipids could be a promising approach for attenuating adverse reactions.

Broad cross-protection against heterologous influenza viruses is highly desirable for vaccines because viruses evolve mutations and evade the immune system. However, HA-$LNP_{ssPalmO}$ and HA-$LNP_{SM\text{-}102}$ did not display HI titers against the heterologous strains. This was attributed to the low homogeneity of HA between the strains. In fact, the amino acid identity of HA between A/Viet Nam/1203/2004 and A/Ezo red fox/Hokkaido/1/2022 is approximately 92%, and most mutations appear in the HA1 region, which contains antigenic sites.[58] In contrast, mRNA-$LNP_{ssPalmO}$ demonstrated better cross-protection against heterologous H5N1 virus challenge compared with mRNA-$LNP_{SM\text{-}102}$, while both mRNA-$LNP_{ssPalmO}$ and mRNA-$LNP_{SM\text{-}102}$ conferred strong protection against the homologous H5N1 virus challenge. This suggests that factors other than neutralizing antibodies, such as non-neutralizing antibodies, are crucial for heterologous protection. Generally, non-neutralizing cross-reactive antibodies can confer cross-protection through Fc-mediated mechanisms such as antibody-dependent cellular cytotoxicity (ADCC) and antibody-dependent cellular phagocytosis.[59] Our previous study showed that both non-neutralizing cross-reactive antibodies and IFN-γ-producing Th1 cells cooperatively contribute to cross-protection against influenza; however, non-neutralizing IgG alone did not provide cross-protection against a heterologous virus.[60] Here, we observed that HA-$LNP_{ssPalmO}$ induces IFN-γ-producing $CD4^+$ T cells more efficiently than HA-$LNP_{SM\text{-}102}$. In addition, HA-$LNP_{ssPalmO}$ and HA-$LNP_{SM\text{-}102}$ failed to elicit detectable HI titer against heterologous viruses, indicating a non-neutralizing anti-HA IgG. Furthermore, we showed that $CD4^+$ T cells contribute to a cross-protective effect of HA-$LNP_{ssPalmO}$. Collectively, the combining of IFN-γ-producing $CD4^+$ T cells and non-neutralizing antibodies in mice immunized with $LNP_{ssPalmO}$ may provide higher cross-protection against heterologous strains.

Moreover, it was unclear how $LNP_{ssPalmO}$ achieved higher IFN-γ-producing $CD4^+$ T cells compared with $LNP_{SM\text{-}102}$. IL-12 is a crucial cytokine that polarizes Th1-mediated cellular immune responses such as IFN-γ-producing $CD4^+$ T cell responses. However, under our experimental conditions, the levels of IL-12 at the injection site and dLNs did not increase in mice immunized with $LNP_{ssPalmO}$ compared to those that received $LNP_{SM\text{-}102}$. In addition, RNA-seq analysis revealed that $LNP_{SM\text{-}102}$ increased inflammatory cytokine responses such as IFN response and chemokines; however, $LNP_{ssPalmO}$ did not. These results suggest that IL-12 and inflammatory cytokine reactions may not be associated with higher IFN-γ-producing $CD4^+$ T cell responses in mice immunized with $LNP_{ssPalmO}$. However, further studies are needed to clarify the identical factors for higher induction of IFN-γ-producing $CD4^+$ T cells.

As the antigenic drift rate of NA is slower than that of HA, anti-NA antibodies provide better cross-protection against heterologous influenza viruses than anti-HA antibodies.[61] However, in the present study, vaccination with NA of A/Viet Nam/1203/2004 was less effective than that with HA for cross-protection against A/Ezo red fox/Hokkaido/1/2022, although the identity of NA and HA between A/Viet Nam/1203/2004 and H1N1 A/California/07/2009 is similar (89% and 92%). The expression of NA is lower than that of HA upon infection.[62] Thus, efficient recognition of NA molecules on infected cells by anti-NA antibodies may be difficult. In addition, He et al. reported that anti-NA antibody is a weaker inducer of ADCC than anti-HA antibody.[63] Therefore, anti-NA antibodies may produce weak viral protection because of their low ability for ADCC, although NA-$LNP_{ssPalmO}$ elicited IFN-γ-producing $CD4^+$ T cells. In contrast, the identity of NA between A/Viet Nam/1203/2004 and H1N1 A/California/07/2009 is around 85%, which is higher than that of HA (64%). Consequently, vaccination with NA of A/Viet Nam/1203/2004 was more effective for cross-protection against H1N1 A/California/07/2009 than that with HA. To improve cross-protective responses against a broad range of influenza viruses, a combination of antigens, such as HA and NA, is desired. Indeed, the combination of HA and NA in vaccines has been reported to enable greater cross-protection than HA or NA alone.[64] In

future studies, we will confirm the efficacy of the combination of HA-$LNP_{ssPalmO}$ and NA-$LNP_{ssPalmO}$ against various types of influenza viruses.

The present study has a limitation. Fever occurs more frequently in booster immunization (29.5%) than in primary immunization (8.6%) in humans.[65] In contrast, in our study, fever occurred in all mice irrespective of primary or booster immunization, indicating the species difference in the ease of inducing adverse reactions between humans and mice. Future studies would be required to investigate whether $LNP_{ssPalmO}$ could be safe for human use as well. In addition, the SS-cleavable site of $LNP_{ssPalmO}$ is cleaved in the reducing environment and spontaneously degraded in the cytoplasm. However, in the present study, whether SS linkages of $LNP_{ssPalmO}$ are involved in high antibody induction and low adverse reactions remains unclear. Despite the limitation, the present study provides important findings regarding the development of LNPs with reduced adverse reactions and the maintenance of or increase in vaccination efficacy.

MATERIALS AND METHODS

Influenza viruses

The H5N1 influenza A virus strains A/Viet Nam/1203/2004,[66] kindly provided by Dr. Le Thi Quynh Mai (National Institute of Hygiene and Epidemiology, Hanoi, Vietnam) and A/Ezo red fox/Hokkaido/1/2022,[38] kindly provided by Drs. Yoshihiro Sakoda, Norikazu Isoda, and Takahiro Hiono (Graduate School of Veterinary Medicine, Hokkaido University, Hokkaido, Japan), were propagated in Madin-Darby canine kidney cells. A virus clone possessing a 627K substitution in the PB2 gene (m29cl5) isolated from the lungs of mice infected with A/Ezo red fox/Hokkaido/1/2022 was used for the challenge study. H1N1 influenza A virus strain A/California/7/2009 was a gift from Dr. Hideki Asanuma (National Institute of Infectious Diseases, Tokyo, Japan). Each experiment using the H5N1 influenza A virus was conducted at the Biosafety Level 3 facility at the Research Institute for Microbial Diseases, Osaka University, in compliance with the guidelines. All the viral experiments were approved by the institutional review board of the Research Institute for Microbial Diseases, Osaka University (protocol nos.: BIKEN-00006-009, BIKEN-00225-013, and BIKEN-00311-005).

Mice

Male C57BL/6J mice (6–7 weeks old) were purchased from Oriental Bio Service (Kyoto, Japan). Male BALB/c (6–7 weeks old) were purchased from Japan SLC (Shizuoka, Japan). Mice were acclimatized and housed under a light-dark cycle condition (12:12-h) with free access to animal feed and water. All animal experiments were performed as per the guidelines of the Animal Care and Use Committee of the Research Institute for Microbial Diseases, Osaka University, Japan (protocol numbers: BIKEN-AP-R01-15-2 and BIKEN-AP-R04-04-0).

Preparation of mRNA-LNP vaccine

HA and NA sequences were derived from the H5N1 influenza A virus (A/Viet Nam/1203/2004, GenBank accession nos. AAW80717.1 for HA and AAT73329.1 for NA). The linearized plasmid DNA was prepared using restriction enzymes. Then, linearized plasmid DNA was purified by phenol-chloroform extraction and ethanol precipitation and subjected to transcription to mRNA by the MEGAscript T7 transcription kit (Thermo Fisher Scientific, Hampton, NH). In the present study, N1-methylpseudouridine was used instead of uridine for mRNA synthesis. The remaining double-stranded RNA was removed as described previously.[67] The 5' cap and 3' poly(A) tail were attached using the ScriptCap Cap 1 Capping System (CellScript, Madison, WI) and the protocol of the poly(A) Tailing Kit (Thermo Fisher Scientific). The final sequences of HA and NA mRNA is shown in Figures S20 and S21. As lipid components of $LNP_{SM\text{-}102,}$ we used SM-102 (Cayman Chemical, Ann Arbor, MI), cholesterol (Sigma-Aldrich, St. Louis, MO), DSPC (NOF Corporation, Tokyo, Japan), and DMG-PEG2000 (NOF Corporation). All lipids were dissolved in ethanol and mixed to achieve an optimal ratio (SM-102:DSPC:cholesterol:DMG-PEG2000 = 50:10:38.5:1.5). We used a lipid mixture in $LNP_{ssPalmO}$ containing ssPalmO (COATSOME SS-OP, NOF Corporation), DOPC (NOF Corporation), cholesterol, and DMG-PEG2000 in an ethanol solution (ssPalmO:DOPC:cholesterol:DMG-PEG2000 = 51.7:7.4:39.4:1.5). To evaluate the effect of the helper lipid difference, we prepared $LNP_{ssPalmO}$ with DSPC composed of ssPalmO:DSPC:cholesterol:DMG-PEG2000 = 51.7:7.4:39.4:1.5. To prepare mRNA-LNPs, mRNA was dissolved in sodium acetate buffer (pH 5.0) and mixed with the lipid mixture using the NanoAssemblr instrument (Precision Nanosystems, Vancouver, Canada) at a flow rate ratio of 3:1. The N:P ratio was set to 5.5. The mRNA-LNP solution was diluted with Dulbecco's PBS and ultrafiltered to remove the external solvents using Amicon Ultra-4-100K spin columns. The physicochemical properties of the mRNA-LNPs were evaluated by measuring the particle size, zeta potential, and encapsulation efficiency using a Zetasizer Nano ZS (Malvern Instruments, Malvern, UK) and Ribogreen reagent (Invitrogen, Carlsbad, CA).

Cryo-EM imaging

Cryo-EM images were obtained using a Talos Arctica electron microscope (FEI, Eindhoven, the Netherlands) equipped with a Falcon III direct electron detector (FEI) and thermal field-emission electron gun operated at 200 kV. Vitrobot IV (FEI) was used to prepare the cryogrids for cryo-EM imaging. For hydrophilizing the surface, the grids (Quantifoil Cu R1.2/1.3 300 mesh, SPT Labtech, Melbourn, UK) were glow discharged for 30 s. mRNA-LNP was diluted with nuclease-free water at a final concentration of 20 mM total lipids and applied at 2.5 μL on a grid at 4°C and 100% humidity. The redundant mRNA-LNP solution was removed by blotting with filter paper. For the cryo-EM image acquisition, the prepared grid was quickly vitrified using liquid ethane.

Recombinant proteins

pcDNA3.1 expression plasmid (Thermo Fisher Scientific) was used to clone the cDNA of the ectodomain of HA (amino acids 1–522, in which 341–345 (RRRKK) was replaced with T) with a hexahistidine tag (His tag) at the C terminus, or of NA (amino acids 51–449) with a His tag at the N terminus. To generate trimeric rHA,

the C terminus of HA was fused with a foldon sequence (GYIPEAPRDGQAYVRKDGEWVLLSTFL, derived from bacteriophage T4 fibritin). To generate a tetrameric rNA, the N terminus of NA was fused with the sequence of the tetrabranchion tetramerization domain (GSIINETADDIVYRLTVIIDDRYESLKNLITLRADRL EMIINDNVSTILASG, derived from the bacterium *Staphylothermus marinus*). The Expi293 expression system (Thermo Fisher Scientific) was used to produce recombinant proteins as previously described.[68] Briefly, 2.5 × 10^6 Expi293F cells/mL were transfected with ExpiFectamine 293 Reagent. The cells were cultured at 37°C under 8% CO_2 on an orbital shaker (120 rpm) for 18 h. ExpiFectamine 293 transfection enhancers 1 and 2 were then added, and the cells were further cultured for 4 days. Recombinant proteins were purified using a Ni-Sepharose HisTrap FF column (GE Healthcare, Chicago, IL) and, subsequently, a Superose 6 Increase 10/300 GL column (GE Healthcare) for size-exclusion chromatography using an AKTA explorer chromatography system.

Immunization

C57BL/6J mice were subcutaneously immunized at the tail base or intramuscularly on days 0 and 21 with mRNA-LNPs (1 μg mRNA/mouse) of expressing HA or NA of the influenza virus. For immunization with the protein, rHA (1 μg) and alum (50 μg) were injected subcutaneously into the tail base. The mice received two subcutaneous immunizations using PBS. Fourteen days after the primary and booster immunization, blood was collected, and plasma was stored at −30°C before use.

Detection of anti-HA and anti-NA antibodies

ELISA was used to determine the plasma levels of anti-HA and anti-NA antibodies as described previously.[60] Briefly, rHA or rNA (1 μg/mL) in 0.1 M sodium carbonate buffer (pH 9.6) was incubated overnight at 4°C in 96-well plates. The coated wells were blocked with Block Ace (DS Pharma Biomedical, Osaka, Japan) and reacted with plasma samples. After washing with 0.05% Tween 20 in PBS, horseradish peroxidase-conjugated goat anti-mouse IgG1, IgG2b, or IgG2c was added and incubated (Table S1). After washing, colorization was initiated by the addition of tetramethyl benzidine (Nacalai Tesque, Kyoto, Japan) and terminated by the addition of 2 N sulfuric acid. Absorbance at $OD_{450-570\ nm}$ was measured using a Power Wave HT microplate reader (BioTek, Winooski, VT). Endpoint titers of HA-specific IgG are shown as the reciprocal log2 of the last dilution that showed above 0.2 absorbance.

HI test

The test sera were treated with the RDE (II) receptor-destroying enzyme (DENKA Company, Tokyo, Japan) according to the manufacturer's protocol before HI testing. The resulting 10-fold dilution of sera was serially diluted 2-fold with PBS in 96-well microplates. Next, 25 μL serum dilutions were mixed with the same volume of 8 HA units of viral antigen and incubated at room temperature for 30 min. Subsequently, 50 μL of 0.5% suspension of chicken erythrocytes (Japan Bioserum Company, Hiroshima, Japan) were mixed with the antigen-serum mixtures and incubated for 30 min at room temperature. HI titers were expressed as the reciprocal of the highest serum dilution showing complete inhibition of hemagglutination.

Evaluating GC B cells using flow cytometry

Fourteen days after the booster immunization, dLNs were subjected to enzymatic processing for 1 h at 37°C with 200 μg/mL Liberase TL (Roche Diagnosis GmbH, Mannheim, Germany) and 10 U DNaseI (Roche Diagnosis GmbH). The cells were blocked with anti-mouse CD16/CD32 antibody and stained with Fixable Viability Dye eFluor 780, Alexa Fluor 700 anti-mouse CD19 antibody, phycoerythrin/cyanine 7 (PE/Cy7) anti-mouse CD95, and Alexa Fluor 647 anti-mouse/human GL7 antibody (Table S1) in PBS containing 2% fetal bovine serum (FBS), 1 mM EDTA (DOJINDO, Kumamoto, Japan), and 0.05% sodium azide (FUJIFILM Wako Pure Chemicals, Osaka, Japan) for 30 min at 4°C in the dark. The cells were analyzed using flow cytometry by adding AccuCheck Counting Beads (Thermo Fisher Scientific) to the samples. Flow cytometric analysis was conducted using an Attune NxT Flow Cytometer (Thermo Fisher Scientific), and data analysis was performed using FlowJo software version 10.9 (TreeStar, Ashland, OR).

Cytokine production from splenocytes

To evaluate T cell cytokine production, on day 14 after booster immunization, splenocytes (1 × 10^6 cells) were collected from mice immunized with mRNA-LNP. Cells were stimulated with rHA or rNA (50 μg/mL) in RPMI1640 with 10% FBS, 1% penicillin-streptomycin, and 50 mM 2-mercaptoethanol at 37°C for 21 h in 96-well plates. The cells were then incubated with a protein transport inhibitor cocktail diluted 1:500 (Thermo Fisher Scientific) for 5 h. The cells were blocked with anti-mouse CD16/CD32 antibody and were stained for 30 min at 4°C with Fixable Viability Dye eFluor 780, Alexa 647 anti-mouse CD45 antibody, PE anti-mouse CD3 antibody, fluorescein isothiocyanate anti-mouse CD4 antibody, BV605 anti-mouse CD8a antibody, and BV510 anti-mouse CD44 antibody (Table S1) in PBS containing 2% FBS, 1 mM EDTA, and 0.05% sodium azide. This was followed by intracellular staining with BV421 anti-IFN-γ antibody and PE/Cy7 anti-IL-13 antibody (Table S1) using a BD Cytofix/Cytoperm Fixation/Permeabilization Solution Kit (BD Biosciences, Sparks, MO) according to the supplier's protocol. Flow cytometry was performed as described previously.

Influenza virus challenge

Fourteen days after the booster immunization, C57BL/6J mice were challenged intranasally with A/Viet Nam/1203/2004 (3.2 × 10^2 PFU), A/Ezo red fox/Hokkaido/1/2022 (3.2 × 10^2 PFU), or A/California/07/2009 (3.0 × 10^4 $TCID_{50}$) suspended in PBS (30 μL) under anesthesia. To deplete $CD4^+$ T cells, anti-CD4 antibody (clone GK1.5) or isotype control antibody (clone LTF-2) (Selleck Chemicals, Houston, TX) was intraperitoneally injected into mice (200 μg/mouse), 1 day before and 3 days after challenge with A/Ezo red fox/Hokkaido/1/2022. After the challenge, changes in body weight and survival rates were observed for 14 days. A body weight reduction of over 25% compared with the body weight before the viral challenge was set as the ethical endpoint. When

the body weight reached less than 75% of the initial body weight, the mice were considered dead.

Evaluation of Luc expression

For evaluating whole imaging of Luc expression, BALB/c mice were intramuscularly injected with Luc-LNP$_{ssPalmO}$ or LNP$_{SM\text{-}102}$ (1 μg mRNA/mouse). Luciferin (FUJIFILM Wako Pure Chemicals) was injected intraperitoneally (30 mg/kg) at the indicated time points (6, 24, 48, and 72 h). Ten minutes after luciferin injection, luminescence images were obtained with an exposure time of 15 s using the *in vivo* imaging system Lumina Series III (PerkinElmer, Waltham, MA) under isoflurane anesthesia. Bioluminescence in the region of interest was analyzed and presented as a photon count (photons/s). For evaluating Luc expression in tissues, mice were intramuscularly injected with Luc-LNP$_{ssPalmO}$ or LNP$_{SM\text{-}102}$ (1 μg mRNA/mouse). At 6 h post-injection, the muscle, dLN, liver, and spleen were collected from euthanized mice. Each tissue sample was weighed and homogenized in PBS using stainless-steel beads and beads crusher μT-12 (Taitec, Saitama, Japan). The supernatant was reacted with the ONE-Glo Luciferase Assay System (Promega, Madison, WI) for 5 min. The luminescence was measured using a GloMax Discover Microplate Reader (Promega).

Evaluation of HA expression

For evaluating HA expression, C57BL/6J mice were intramuscularly immunized on days 0 and 21 with 1 μg mRNA of HA-LNP$_{ssPalmO}$ or LNP$_{SM\text{-}102}$. At 24 h after the booster immunization, the minced muscle was incubated with collagenase type 2 (0.2%, Worthington, Lakewood, NJ) and DNaseI (200 U/mL) for 1 h at 37°C. After homogenization using a syringe, the suspension was additionally incubated for 30 min at 37°C. Finally, the suspension was homogenized with a GentleMACS dissociator (Miltenyi Biotec, Bergisch Gladbach, Germany) using the m-Muscle program. The muscle cells were stained for 30 min at 4°C in the dark with Fixable Viability Dye eFluor 780, BV421 anti-mouse CD45, PE-Dazzle594 anti-mouse CD11c, and Alexa Fluor 700 anti-mouse I-A/I-E antibody (Table S1) in PBS containing 2% FBS, 1 mM EDTA, and 0.05% sodium azide. The cell surface HA was stained with anti-HA rabbit IgG and PE-anti-rabbit IgG antibodies (Table S1). The cells were analyzed using flow cytometry, as described above.

Activation of APCs

To evaluate APC activation, dLNs were collected 24 h after immunization. Single-cell suspensions were prepared by incubation with 200 μg/mL Liberase TL and 10 U DNaseI for 1 h at 37°C. Following this, the cells were blocked with anti-mouse CD16/CD32 antibody and were stained for 30 min at 4°C in the dark with Fixable Viability Dye eFluor 780, PerCP/Cy5.5 anti-mouse CD11c antibody, APC anti-mouse PDCA1 antibody, BV421 anti-mouse I-A/I-E antibody, Alexa Fluor 700 anti-mouse CD19 antibody, PE/Cy7 anti-mouse CD11b, and PE anti-mouse CD86 antibody (Table S1) in PBS containing 2% FBS, 1 mM EDTA, and 0.05% sodium azide. The cells were analyzed by flow cytometry, as described above.

RNA-seq

For RNA-seq analysis of dLNs, dLNs were collected 6 h after immunization, whereas single-cell suspensions of dLNs were prepared by incubating 200 μg/mL Liberase TL and 10 U DNaseI for 1 h at 37°C for RNA-seq analysis of dLNs or DCs in dLNs. The cells were stained for 30 min at 4°C in the dark with Fixable Viability Dye eFluor 780, Alexa Fluor 488 anti-mouse CD90.2, Alexa Fluor 700 anti-mouse I-A/I-E antibody, BV421 anti-mouse CD45, PE-Dazzle594 anti-mouse CD11c, and PE/Cy7 anti-mouse CD19 antibody (Table S1) in PBS containing 2% FBS, 1 mM EDTA, and 0.05% sodium azide. The DCs were sorted by the FACSAria III cell sorter. Total RNA was extracted from the dLNs using RNA Cultured Cell Kit and TissueLyser II (Qiagen, Hilden, Germany) following the manufacturer's instructions. RNA libraries were prepared using a TruSeq Stranded mRNA Library Prep Kit (Illumina, San Diego, CA) based on the manufacturer's instructions. Additionally, sequencing was performed on the NovaSeq 6000 platform in a 101-base single-end mode, and RTA version 3.4.4 software (Illumina) was used for base calling. Generated reads were mapped to the mouse (GRCm38) reference genome using HISAT2 version 2.1.0. In addition, fragments per kilobase of exon per million mapped fragments were calculated using Cuffdiff version 2.2.1 with parameter-max-bundle-frags 50,000,000. Principal-component analysis, heatmap clustering, volcano plot analysis, and enrichment analysis were performed using iDEP 2.01 (http://bioinformatics.sdstate.edu/idep/) and RNAseqChef (https://imeg-ku.shinyapps.io/RNAseqChef/). Notably, the RNA-seq data concerning this study have been deposited in the GEO under accession numbers GSE279743 and GSE279744.

Cytokine and chemokine production in blood

For evaluating cytokine and chemokine production, C57BL/6J mice were immunized with 1 μg mRNA of HA-LNP twice at 21-day intervals. Plasma samples were collected from mice 6 h after the primary and booster immunizations. The plasma levels of IFN-α, IFN-β, IFN-γ, CCL2 (monocyte chemoattractant protein-1), CXCL10 (IFN-γ inducible protein 10), IL-6, CXCL1 (KC), TNF-α, IL-12 p70, CCL5 (RANTES), IL-1β, GM-CSF, and IL-10 were determined using the LEGENDplex Mouse Anti-Virus Response Panel (13-plex) (BioLegend, San Diego, CA) according to the manufacturer's instructions. Briefly, the standard and experimental samples were diluted 2-fold using assay buffer and incubated with capture beads. The plate was reacted with detection beads and further reacted with streptavidin-PE after washing. Fluorescent signals were analyzed using flow cytometry using LEGENDplex data analysis software (BioLegend).

mRNA expression of inflammatory cytokines in hearts

We immunized C57BL/6J mice intramuscularly with 10 μg mRNA of spike-LNP. The hearts were collected from the mice on days 1 and 2 after immunizations. The hearts collected were in 0.5 mL TRIzol Reagent (Thermo Fisher Scientific) and were homogenized with stainless-steel beads and beads crusher μT-12. Furthermore, RNA was purified using TRIzol reagent following the manufacturer's instructions. We performed reverse transcription using ReverTra Ace qPCR RT

Master Mix with gDNA Remover (Toyobo, Osaka, Japan) to synthesize cDNA. Real-time reverse transcription PCR was performed by amplifying the target mRNA and *Gapdh* mRNA as a control gene using a Light Cycler 480-II (Roche Diagnostics, Tokyo, Japan) and LightCycler 480 SYBR Green I Master (Roche Diagnostics). The primers for each mRNA were used as described in Table S2. We calculated the relative expression level of mRNA by dividing the target mRNA expression levels by *Gapdh* mRNA expression levels, with the mean value of the control group expressed as 1.

Evaluation of vascular permeability, body weight change, and fever

To evaluate the vascular permeability caused by mRNA-LNP, C57BL/6J mice were intramuscularly immunized in the left tibialis with 1 μg mRNA of HA-$LNP_{ssPalmO}$ or LNP_{SM-102}. After 6 h, Evans blue (2%) was injected intravenously into the mice. Thirty minutes later, the left tibialis was collected and incubated in formamide at 55°C for 48 h for the extraction of Evans blue. The absorbance of the supernatant was measured at 620 nm using a microplate reader. For evaluating body weight change, C57BL/6J mice were intramuscularly immunized with 20 μg mRNA of HA-$LNP_{ssPalmO}$ or LNP_{SM-102} following the measurement of body weight. At 24 h, the body weight was measured again. To evaluate fever, C57BL/6J mice were intramuscularly immunized with 5 μg mRNA of HA-$LNP_{ssPalmO}$ or LNP_{SM-102}. At 6 h, the rectal temperature was measured using a rectal probe (Natsume Seisakusho, Tokyo, Japan).

Statistics

All experiments were performed in duplicate. Statistical analyses were conducted using GraphPad Prism version 9.0.0 (GraphPad Software, San Diego, CA). All data are expressed as means ± standard deviations (SDs). Significant differences in survival rates were obtained by comparing Kaplan-Meier curves using the log rank test. One-way analysis of variance (ANOVA) followed by Tukey's test were performed to compare more than two sets of data. Statistical significance was set at $p < 0.05$.

DATA AND CODE AVAILABILITY

All data are included in the paper or the supplemental information. Additional data are available from the corresponding authors on reasonable request.

ACKNOWLEDGMENTS

We thank Drs. Yoshihiro Sakoda, Norikazu Isoda, and Takahiro Hiono for providing H5N1 influenza A virus strain A/Ezo red fox/Hokkaido/1/2022. This study was supported by grants from the Japan Agency for Medical Research and Development (AMED), Japan (AMED grant nos. 21am0401030h0001, 22am0401030h0002, 23am0401030h0003, and 233fa827018h0001 to Y.Y., and JP223fa627002 to T.W. and Y.Y.), the AMED Advanced Research and Development Programs for Medical Innovation (AMED-CREST) (JP22gm1610010 to T.W.), the All-Osaka U Research in "The Nippon Foundation–Osaka University Project for Infectious Disease Project" (to Y.Y.), and The Research Foundation for Microbial Diseases of Osaka University (BIKEN), Japan.

AUTHOR CONTRIBUTIONS

The manuscript was written with contributions from all authors. All the authors approved the final version of the manuscript.

DECLARATION OF INTERESTS

T.S., H.T., T.W., H.A., and Y.Y. filed a patent application related to the content of the manuscript (US63/515,413 and PCT/JP2024/026712). H.T. and H.A. are the named inventors on a patent (WO2019/188867). Y.Y. is an employee of The Research Foundation for Microbial Diseases of Osaka University, Osaka, Japan.

SUPPLEMENTAL INFORMATION

Supplemental information can be found online at https://doi.org/10.1016/j.ymthe.2024.12.032.

REFERENCES

1. Pardi, N., Hogan, M.J., Porter, F.W., and Weissman, D. (2018). mRNA vaccines - a new era in vaccinology. Nat. Rev. Drug Discov. *17*, 261–279.
2. Kowalzik, F., Schreiner, D., Jensen, C., Teschner, D., Gehring, S., and Zepp, F. (2021). mRNA-Based Vaccines. Vaccines (Basel) *9*, 390.
3. Jackson, L.A., Anderson, E.J., Rouphael, N.G., Roberts, P.C., Makhene, M., Coler, R.N., McCullough, M.P., Chappell, J.D., Denison, M.R., Stevens, L.J., et al. (2020). An mRNA Vaccine against SARS-CoV-2 - Preliminary Report. N. Engl. J. Med. *383*, 1920–1931.
4. Polack, F.P., Thomas, S.J., Kitchin, N., Absalon, J., Gurtman, A., Lockhart, S., Perez, J.L., Pérez Marc, G., Moreira, E.D., Zerbini, C., et al. (2020). Safety and Efficacy of the BNT162b2 mRNA Covid-19 Vaccine. N. Engl. J. Med. *383*, 2603–2615.
5. Florea, A., Sy, L.S., Qian, L., Ackerson, B.K., Luo, Y., Tubert, J.E., Lee, G.S., Ku, J.H., Bruxvoort, K.J., Talarico, C.A., et al. (2023). Effectiveness of Messenger RNA-1273 Vaccine Booster Against Coronavirus Disease 2019 in Immunocompetent Adults. Clin. Infect. Dis. *76*, 252–262.
6. Barbier, A.J., Jiang, A.Y., Zhang, P., Wooster, R., and Anderson, D.G. (2022). The clinical progress of mRNA vaccines and immunotherapies. Nat. Biotechnol. *40*, 840–854.
7. Kim, M.S., Jung, S.Y., Ahn, J.G., Park, S.J., Shoenfeld, Y., Kronbichler, A., Koyanagi, A., Dragioti, E., Tizaoui, K., Hong, S.H., et al. (2022). Comparative safety of mRNA COVID-19 vaccines to influenza vaccines: A pharmacovigilance analysis using WHO international database. J. Med. Virol. *94*, 1085–1095.
8. Sahin, U., Muik, A., Derhovanessian, E., Vogler, I., Kranz, L.M., Vormehr, M., Baum, A., Pascal, K., Quandt, J., Maurus, D., et al. (2020). COVID-19 vaccine BNT162b1 elicits human antibody and T_H1 T cell responses. Nature *586*, 594–599.
9. Walsh, E.E., Frenck, R.W., Jr., Falsey, A.R., Kitchin, N., Absalon, J., Gurtman, A., Lockhart, S., Neuzil, K., Mulligan, M.J., Bailey, R., et al. (2020). Safety and Immunogenicity of Two RNA-Based Covid-19 Vaccine Candidates. N. Engl. J. Med. *383*, 2439–2450.
10. Kadali, R.A.K., Janagama, R., Peruru, S., and Malayala, S.V. (2021). Side effects of BNT162b2 mRNA COVID-19 vaccine: A randomized, cross-sectional study with detailed self-reported symptoms from healthcare workers. Int. J. Infect. Dis. *106*, 376–381.
11. Goda, K., Kenzaka, T., Yahata, S., Okayama, M., and Nishisaki, H. (2022). Association between Adverse Reactions to the First and Second Doses of COVID-19 Vaccine. Vaccines (Basel) *10*, 1232.
12. Najjar, M., Albuaini, S., Fadel, M., Mohsen, F., Data Collection Group, Najjar, G., Assaf, D., Aljbawi, A., and AlAwad, Y. (2023). Covid-19 vaccination reported side effects and hesitancy among the Syrian population: a cross-sectional study. Ann. Med. *55*, 2241351.
13. Mohsin, M., Mahmud, S., Uddin Mian, A., Hasan, P., Muyeed, A., Taif Ali, M., Faysal Ahmed, F., Islam, A., Maliha Rahman, M., Islam, M., et al. (2022). Side effects of COVID-19 vaccines and perceptions about COVID-19 and its vaccines in Bangladesh: A Cross-sectional study. Vaccine X *12*, 100207.
14. Dinarello, C.A. (2018). Overview of the IL-1 family in innate inflammation and acquired immunity. Immunol. Rev. *281*, 8–27.
15. Tanaka, T., Narazaki, M., and Kishimoto, T. (2014). IL-6 in inflammation, immunity, and disease. Cold Spring Harb. Perspect. Biol. *6*, a016295.
16. Arunachalam, P.S., Scott, M.K.D., Hagan, T., Li, C., Feng, Y., Wimmers, F., Grigoryan, L., Trisal, M., Edara, V.V., Lai, L., et al. (2021). Systems vaccinology of the BNT162b2 mRNA vaccine in humans. Nature *596*, 410–416.

17. Bergamaschi, C., Terpos, E., Rosati, M., Angel, M., Bear, J., Stellas, D., Karaliota, S., Apostolakou, F., Bagratuni, T., Patseas, D., et al. (2021). Systemic IL-15, IFN-gamma, and IP-10/CXCL10 signature associated with effective immune response to SARS-CoV-2 in BNT162b2 mRNA vaccine recipients. Cell Rep. *36*, 109504.

18. Li, C., Lee, A., Grigoryan, L., Arunachalam, P.S., Scott, M.K.D., Trisal, M., Wimmers, F., Sanyal, M., Weidenbacher, P.A., Feng, Y., et al. (2022). Mechanisms of innate and adaptive immunity to the Pfizer-BioNTech BNT162b2 vaccine. Nat. Immunol. *23*, 543–555.

19. Miyashita, Y., Yoshida, T., Takagi, Y., Tsukamoto, H., Takashima, K., Kouwaki, T., Makino, K., Fukushima, S., Nakamura, K., and Oshiumi, H. (2022). Circulating extracellular vesicle microRNAs associated with adverse reactions, proinflammatory cytokine, and antibody production after COVID-19 vaccination. NPJ Vaccin. *7*, 16.

20. Cullis, P.R., and Hope, M.J. (2017). Lipid Nanoparticle Systems for Enabling Gene Therapies. Mol. Ther. *25*, 1467–1475.

21. Kulkarni, J.A., Cullis, P.R., and van der Meel, R. (2018). Lipid Nanoparticles Enabling Gene Therapies: From Concepts to Clinical Utility. Nucleic Acid Ther. *28*, 146–157.

22. Alameh, M.G., Tombacz, I., Bettini, E., Lederer, K., Sittplangkoon, C., Wilmore, J.R., Gaudette, B.T., Soliman, O.Y., Pine, M., Hicks, P., et al. (2021). Lipid nanoparticles enhance the efficacy of mRNA and protein subunit vaccines by inducing robust T follicular helper cell and humoral responses. Immunity *54*, 2877–2892.

23. Lonez, C., Bessodes, M., Scherman, D., Vandenbranden, M., Escriou, V., and Ruysschaert, J.M. (2014). Cationic lipid nanocarriers activate Toll-like receptor 2 and NLRP3 inflammasome pathways. Nanomedicine *10*, 775–782.

24. Lonez, C., Irvine, K.L., Pizzuto, M., Schmidt, B.I., Gay, N.J., Ruysschaert, J.M., Gangloff, M., and Bryant, C.E. (2015). Critical residues involved in Toll-like receptor 4 activation by cationic lipid nanocarriers are not located at the lipopolysaccharide-binding interface. Cell. Mol. Life Sci. *72*, 3971–3982.

25. Tahtinen, S., Tong, A.J., Himmels, P., Oh, J., Paler-Martinez, A., Kim, L., Wichner, S., Oei, Y., McCarron, M.J., Freund, E.C., et al. (2022). IL-1 and IL-1ra are key regulators of the inflammatory response to RNA vaccines. Nat. Immunol. *23*, 532–542.

26. Ndeupen, S., Qin, Z., Jacobsen, S., Bouteau, A., Estanbouli, H., and Igyártó, B.Z. (2021). The mRNA-LNP platform's lipid nanoparticle component used in preclinical vaccine studies is highly inflammatory. iScience *24*, 103479.

27. Parhiz, H., Brenner, J.S., Patel, P.N., Papp, T.E., Shahnawaz, H., Li, Q., Shi, R., Zamora, M.E., Yadegari, A., Marcos-Contreras, O.A., et al. (2022). Added to pre-existing inflammation, mRNA-lipid nanoparticles induce inflammation exacerbation (IE). J. Control Release *344*, 50–61.

28. Oyama, R., Ishigame, H., Tanaka, H., Tateshita, N., Itazawa, M., Imai, R., Nishiumi, N., Kishikawa, J.I., Kato, T., Anindita, J., et al. (2023). An Ionizable Lipid Material with a Vitamin E Scaffold as an mRNA Vaccine Platform for Efficient Cytotoxic T Cell Responses. ACS Nano *17*, 18758–18774.

29. Akita, H., Ishiba, R., Hatakeyama, H., Tanaka, H., Sato, Y., Tange, K., Arai, M., Kubo, K., and Harashima, H. (2013). A neutral envelope-type nanoparticle containing pH-responsive and SS-cleavable lipid-like material as a carrier for plasmid DNA. Adv. Healthc. Mater. *2*, 1120–1125.

30. Akita, H. (2020). Development of an SS-Cleavable pH-Activated Lipid-Like Material (ssPalm) as a Nucleic Acid Delivery Device. Biol. Pharm. Bull. *43*, 1617–1625.

31. Tanaka, H., Takahashi, T., Konishi, M., Takata, N., Gomi, M., Shirane, D., Miyama, R., Hagiwara, S., Yamasaki, Y., Sakurai, Y., et al. (2020). Self-Degradable Lipid-Like Materials Based on "Hydrolysis accelerated by the intra-Particle Enrichment of Reactant (HyPER)" for Messenger RNA Delivery. Adv. Funct. Mater. *30*, 1910575.

32. Tanaka, H., Takata, N., Sakurai, Y., Yoshida, T., Inoue, T., Tamagawa, S., Nakai, Y., Tange, K., Yoshioka, H., Maeki, M., et al. (2021). Delivery of Oligonucleotides Using a Self-Degradable Lipid-Like Material. Pharmaceutics *13*, 544.

33. Caliendo, V., Lewis, N.S., Pohlmann, A., Baillie, S.R., Banyard, A.C., Beer, M., Brown, I.H., Fouchier, R.A.M., Hansen, R.D.E., Lameris, T.K., et al. (2022). Transatlantic spread of highly pathogenic avian influenza H5N1 by wild birds from Europe to North America in 2021. Sci. Rep. *12*, 11729.

34. Shi, J., Zeng, X., Cui, P., Yan, C., and Chen, H. (2023). Alarming situation of emerging H5 and H7 avian influenza and effective control strategies. Emerg. Microbes Infect. *12*, 2155072.

35. Soilemetzidou, E.S., De Bruin, E., Franz, M., Aschenborn, O.H.K., Rimmelzwaan, G.F., van Beek, R., Koopmans, M., Greenwood, A.D., and Czirják, G.Á. (2020). Diet May Drive Influenza A Virus Exposure in African Mammals. J. Infect. Dis. *221*, 175–182.

36. World Health Organization (2024). Disease Outbreak News; Avian Influenza A(H5N1) - United States of America. https://www.who.int/emergencies/disease-outbreak-news/item/2024-DON512.

37. Baden, L.R., El Sahly, H.M., Essink, B., Kotloff, K., Frey, S., Novak, R., Diemert, D., Spector, S.A., Rouphael, N., Creech, C.B., et al. (2021). Efficacy and Safety of the mRNA-1273 SARS-CoV-2 Vaccine. N. Engl. J. Med. *384*, 403–416.

38. Hiono, T., Kobayashi, D., Kobayashi, A., Suzuki, T., Satake, Y., Harada, R., Matsuno, K., Sashika, M., Ban, H., Kobayashi, M., et al. (2023). Virological, pathological, and glycovirological investigations of an Ezo red fox and a tanuki naturally infected with H5N1 high pathogenicity avian influenza viruses in Hokkaido, Japan. Virology *578*, 35–44.

39. Bitounis, D., Jacquinet, E., Rogers, M.A., and Amiji, M.M. (2024). Strategies to reduce the risks of mRNA drug and vaccine toxicity. Nat. Rev. Drug Discov. *23*, 281–300.

40. Jorgensen, A.M., Wibel, R., and Bernkop-Schnurch, A. (2023). Biodegradable Cationic and Ionizable Cationic Lipids: A Roadmap for Safer Pharmaceutical Excipients. Small *19*, e2206968.

41. Burdette, D., Ci, L., Shilliday, B., Slauter, R., Auerbach, A., Kenney, M., Almarsson, Ö., Cheung, E., and Hendrick, T. (2023). Systemic Exposure, Metabolism, and Elimination of [^{14}C]-Labeled Amino Lipid, Lipid 5, after a Single Administration of mRNA Encapsulating Lipid Nanoparticles to Sprague-Dawley Rats. Drug Metab. Dispos. *51*, 804–812.

42. Verlengia, R., Gorjão, R., Kanunfre, C.C., Bordin, S., de Lima, T.M., and Curi, R. (2003). Effect of arachidonic acid on proliferation, cytokines production and pleiotropic genes expression in Jurkat cells–a comparison with oleic acid. Life Sci. *73*, 2939–2951.

43. Pegoraro, N.S., Camponogara, C., Cruz, L., and Oliveira, S.M. (2021). Oleic acid exhibits an expressive anti-inflammatory effect in croton oil-induced irritant contact dermatitis without the occurrence of toxicological effects in mice. J. Ethnopharmacol. *267*, 113486.

44. Yu, H.P., Liu, F.C., Umoro, A., Lin, Z.C., Elzoghby, A.O., Hwang, T.L., and Fang, J.Y. (2020). Oleic acid-based nanosystems for mitigating acute respiratory distress syndrome in mice through neutrophil suppression: how the particulate size affects therapeutic efficiency. J. Nanobiotechnology *18*, 25.

45. Cheng, X., and Lee, R.J. (2016). The role of helper lipids in lipid nanoparticles (LNPs) designed for oligonucleotide delivery. Adv. Drug Deliv. Rev. *99*, 129–137.

46. Qiu, M., Glass, Z., Chen, J., Haas, M., Jin, X., Zhao, X., Rui, X., Ye, Z., Li, Y., Zhang, F., and Xu, Q. (2021). Lipid nanoparticle-mediated codelivery of Cas9 mRNA and single-guide RNA achieves liver-specific *in vivo* genome editing of *Angptl3*. Proc. Natl. Acad. Sci. USA *118*, e2020401118.

47. Kulkarni, J.A., Myhre, J.L., Chen, S., Tam, Y.Y.C., Danescu, A., Richman, J.M., and Cullis, P.R. (2017). Design of lipid nanoparticles for *in vitro* and *in vivo* delivery of plasmid DNA. Nanomedicine. *13*, 1377–1387.

48. Evans, S.S., Repasky, E.A., and Fisher, D.T. (2015). Fever and the thermal regulation of immunity: the immune system feels the heat. Nat. Rev. Immunol. *15*, 335–349.

49. Fahey, E., and Doyle, S.L. (2019). IL-1 Family Cytokine Regulation of Vascular Permeability and Angiogenesis. Front. Immunol. *10*, 1426.

50. Phillips, C.L., and Grayson, B.E. (2020). The immune remodel: Weight loss-mediated inflammatory changes to obesity. Exp. Biol. Med. *245*, 109–121.

51. Lee, S., Lee, J., Cho, S.H., Roh, G., Park, H.J., Lee, Y.J., Jeon, H.E., Lee, Y.S., Bae, S.H., Youn, S.B., et al. (2024). Assessing the impact of mRNA vaccination in chronic inflammatory murine model. NPJ Vaccin. *9*, 34.

52. Li, C., Chen, Y., Zhao, Y., Lung, D.C., Ye, Z., Song, W., Liu, F.F., Cai, J.P., Wong, W.M., Yip, C.C.Y., et al. (2022). Intravenous Injection of Coronavirus Disease 2019 (COVID-19) mRNA Vaccine Can Induce Acute Myopericarditis in Mouse Model. Clin. Infect. Dis. *74*, 1933–1950.

53. Nance, K.D., and Meier, J.L. (2021). Modifications in an Emergency: The Role of N1-Methylpseudouridine in COVID-19 Vaccines. ACS Cent. Sci. *7*, 748–756.

54. Schlee, M., and Hartmann, G. (2016). Discriminating self from non-self in nucleic acid sensing. Nat. Rev. Immunol. *16*, 566–580.

55. Takano, T., Morikawa, M., Adachi, Y., Kabasawa, K., Sax, N., Moriyama, S., Sun, L., Isogawa, M., Nishiyama, A., Onodera, T., et al. (2022). Distinct immune cell dynamics correlate with the immunogenicity and reactogenicity of SARS-CoV-2 mRNA vaccine. Cell Rep. Med. *3*, 100631.

56. Xu, Y., Hu, Y., Xia, H., Zhang, S., Lei, H., Yan, B., Xiao, Z.X., Chen, J., Pang, J., and Zha, G.F. (2024). Delivery of mRNA Vaccine with 1, 2-Diesters-Derived Lipids Elicits Fast Liver Clearance for Safe and Effective Cancer Immunotherapy. Adv. Healthc. Mater. *13*, e2302691.

57. Yang, K., Bai, B., Lei, J., Yu, X., Qi, S., Wang, Y., Huang, F., Tong, Z., and Yu, G. (2024). Biodegradable Lipid-Modified Poly(Guanidine Thioctic Acid)s: A Fortifier of Lipid Nanoparticles to Promote the Efficacy and Safety of mRNA Cancer Vaccines. J. Am. Chem. Soc. *146*, 11679–11693.

58. Chiu, F.F., Venkatesan, N., Wu, C.R., Chou, A.H., Chen, H.W., Lian, S.P., Liu, S.J., Huang, C.C., Lian, W.C., Chong, P., and Leng, C.H. (2009). Immunological study of HA1 domain of hemagglutinin of influenza H5N1 virus. Biochem. Biophys. Res. Commun. *383*, 27–31.

59. Chandler, T.L., Yang, A., Otero, C.E., Permar, S.R., and Caddy, S.L. (2023). Protective mechanisms of nonneutralizing antiviral antibodies. PLoS Pathog. *19*, e1011670.

60. Shibuya, M., Tamiya, S., Kawai, A., Hirai, T., Cragg, M.S., and Yoshioka, Y. (2021). Synergistic effect of non-neutralizing antibodies and interferon-gamma for cross-protection against influenza. iScience *24*, 103131.

61. Sandbulte, M.R., Westgeest, K.B., Gao, J., Xu, X., Klimov, A.I., Russell, C.A., Burke, D.F., Smith, D.J., Fouchier, R.A.M., and Eichelberger, M.C. (2011). Discordant antigenic drift of neuraminidase and hemagglutinin in H1N1 and H3N2 influenza viruses. Proc. Natl. Acad. Sci. USA *108*, 20748–20753.

62. Hutchinson, E.C., Charles, P.D., Hester, S.S., Thomas, B., Trudgian, D., Martínez-Alonso, M., and Fodor, E. (2014). Conserved and host-specific features of influenza virion architecture. Nat. Commun. *5*, 4816.

63. He, W., Tan, G.S., Mullarkey, C.E., Lee, A.J., Lam, M.M.W., Krammer, F., Henry, C., Wilson, P.C., Ashkar, A.A., Palese, P., and Miller, M.S. (2016). Epitope specificity plays a critical role in regulating antibody-dependent cell-mediated cytotoxicity against influenza A virus. Proc. Natl. Acad. Sci. USA *113*, 11931–11936.

64. Cheung, M., Chang, C., Rathnasinghe, R., Rossignol, E., Zhang, Y., Ferrari, A., Patel, H., Huang, Y., Sanchez Guillen, M., Scalzo, T., et al. (2023). Self-amplifying mRNA seasonal influenza vaccines elicit mouse neutralizing antibody and cell-mediated immunity and protect ferrets. NPJ Vaccin. *8*, 150.

65. Chapin-Bardales, J., Gee, J., and Myers, T. (2021). Reactogenicity Following Receipt of mRNA-Based COVID-19 Vaccines. JAMA *325*, 2201–2202.

66. Le, Q.M., Ito, M., Muramoto, Y., Hoang, P.V.M., Vuong, C.D., Sakai-Tagawa, Y., Kiso, M., Ozawa, M., Takano, R., and Kawaoka, Y. (2010). Pathogenicity of highly pathogenic avian H5N1 influenza A viruses isolated from humans between 2003 and 2008 in northern Vietnam. J. Gen. Virol. *91*, 2485–2490.

67. Baiersdorfer, M., Boros, G., Muramatsu, H., Mahiny, A., Vlatkovic, I., Sahin, U., and Kariko, K. (2019). A Facile Method for the Removal of dsRNA Contaminant from In Vitro-Transcribed mRNA. Mol. Ther. Nucleic Acids *15*, 26–35.

68. Kawai, A., Yamamoto, Y., Nogimori, T., Takeshita, K., Yamamoto, T., and Yoshioka, Y. (2021). The Potential of Neuraminidase as an Antigen for Nasal Vaccines To Increase Cross-Protection against Influenza Viruses. J. Virol. *95*, e0118021.

YMTHE, Volume *33*

Supplemental Information

Low-inflammatory lipid nanoparticle-based mRNA vaccine elicits protective immunity against H5N1 influenza virus with reduced adverse reactions

Atsushi Kawai, Taro Shimizu, Hiroki Tanaka, Shintaro Shichinohe, Jessica Anindita, Mika Hirose, Eigo Kawahara, Kota Senpuku, Makoto Shimooka, Le Thi Quynh Mai, Ryo Suzuki, Takuto Nogimori, Takuya Yamamoto, Toshiro Hirai, Takayuki Kato, Tokiko Watanabe, Hidetaka Akita, and Yasuo Yoshioka

(a)

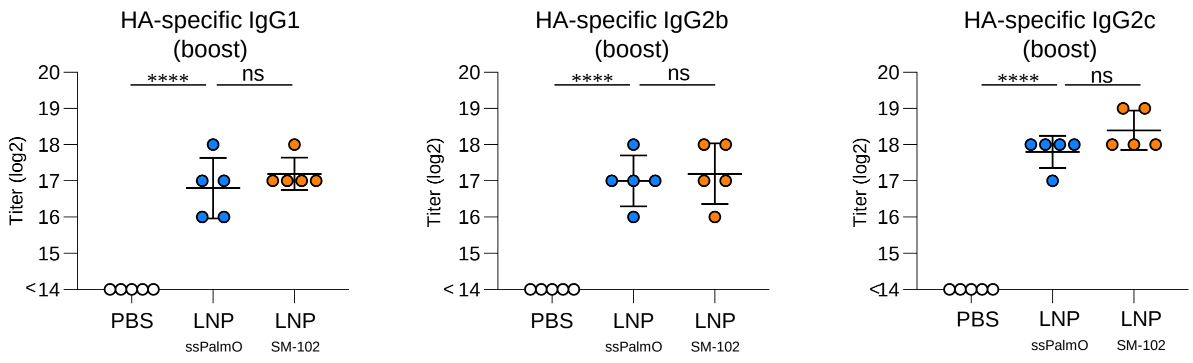

(b)

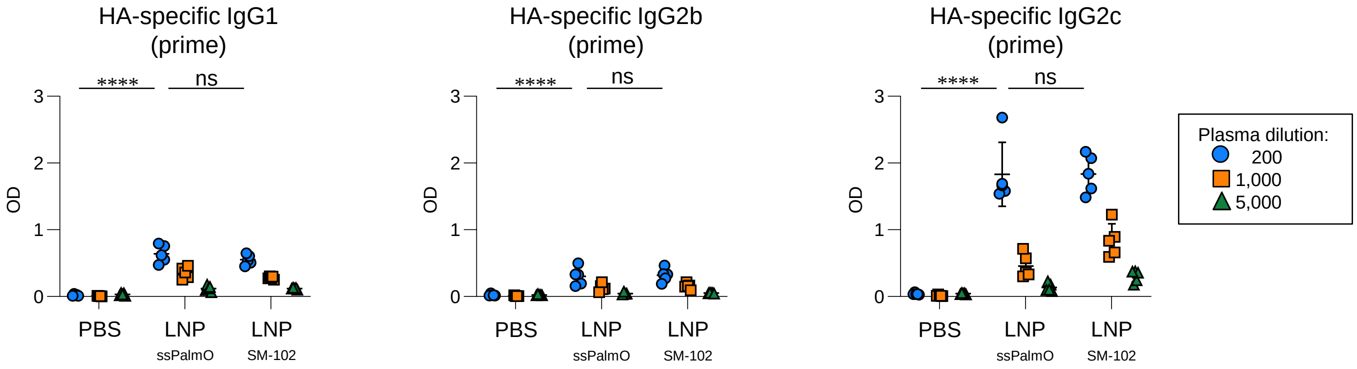

Figure S1. Antibody responses against HA following subcutaneous immunization with HA-LNP. Mice were subcutaneously immunized with HA-LNP on days 0 (prime) and 21 (boost). (a) The titers of HA-specific IgG1, IgG2b, and IgG2c in the plasma on day 35 were evaluated using ELISA. These data are related to Figure 2a. (b) Plasma levels of HA-specific IgG1, IgG2b, and IgG2c on day 14 were evaluated using ELISA. (a, b) n=5 per group. Data are means ± SD. $^{****}P < 0.0001$, Tukey's multiple-comparisons test. ns, not statistically significant. (b) Tukey's multiple comparison test performed at a dilution of 200.

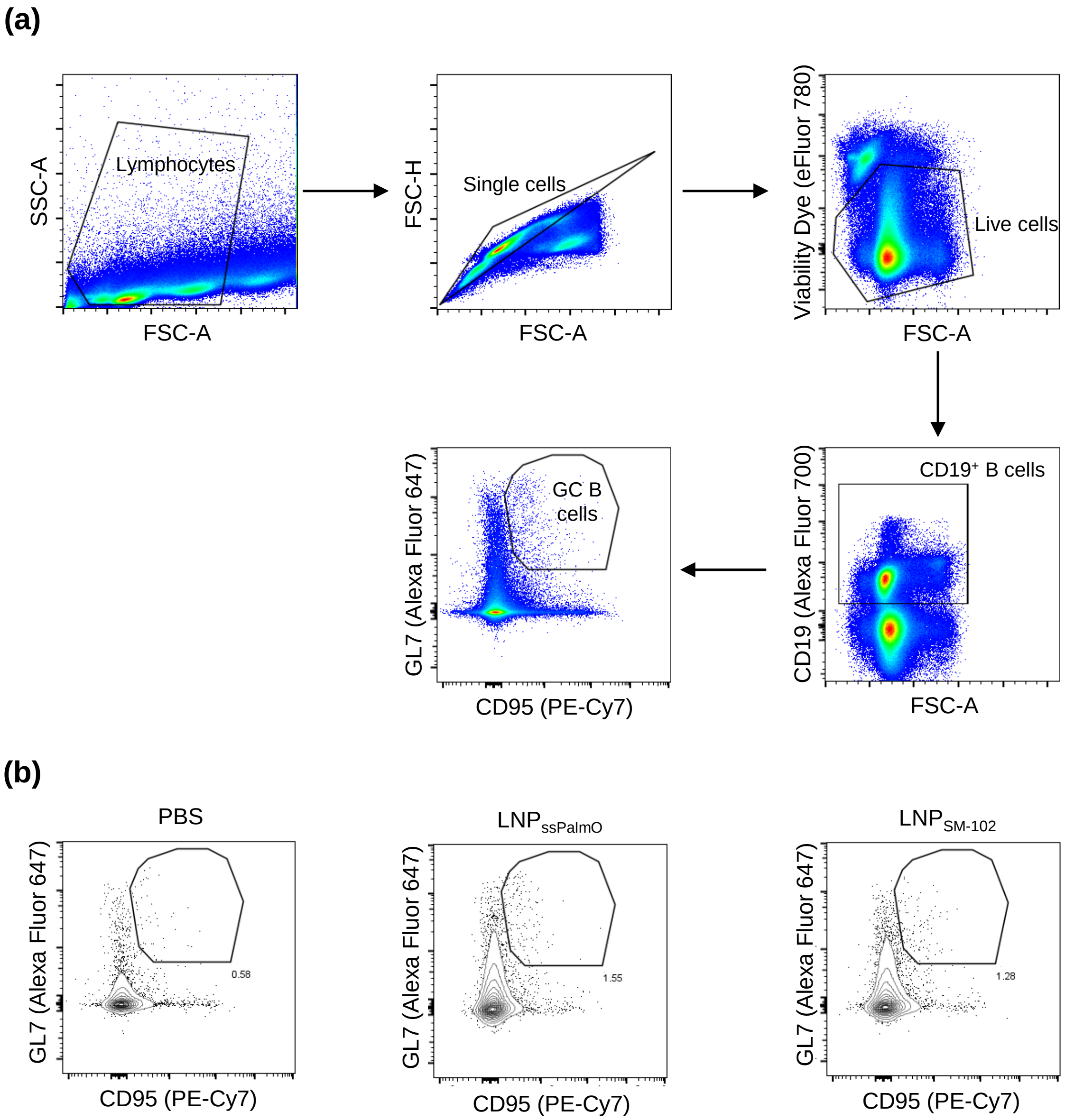

Figure S2. Flow cytometry analysis of germinal center B cells.
(a) Gating strategy and (b) representative plot of germinal center B cells. The data are related to those in Figure 2b.

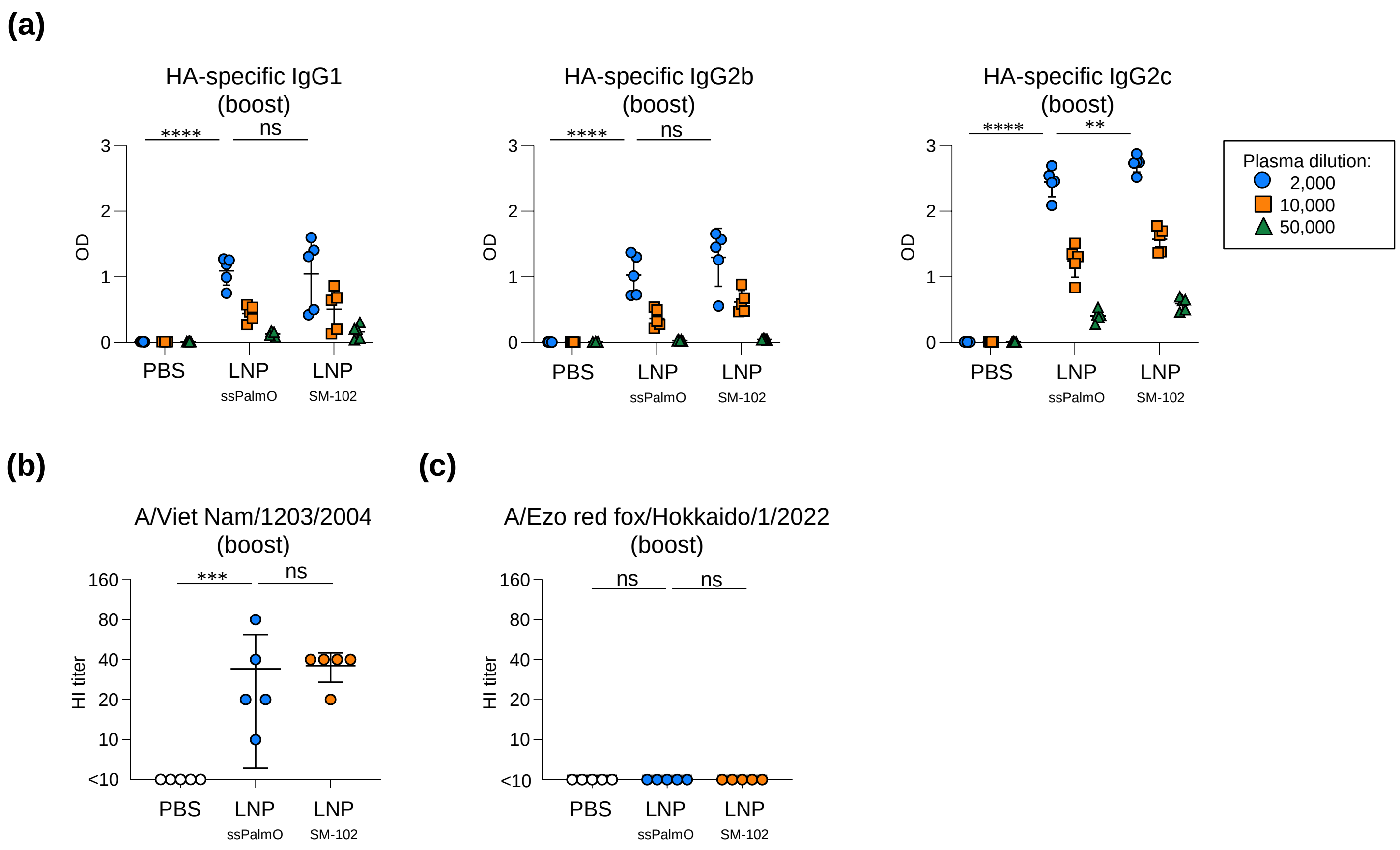

Figure S3. Antibody responses against HA following intramuscular immunization with HA-LNP. Mice were immunized intramuscularly with HA-LNP on days 0 (prime) and 21 (boost). (a) Plasma levels of HA-specific IgG1, IgG2b, and IgG2c on day 35 were analyzed using ELISA. (b, c) Hemagglutination inhibition titer of plasma on day 35 was assessed against H5N1 influenza A virus strains (b) A/Viet Nam/1203/2004 and (c) A/Ezo red fox/Hokkaido/1/2022. (a-c) n=5 per group. Data are means ± SD. $^{**}P < 0.01$; $^{***}P < 0.001$; $^{****}P < 0.0001$, Tukey's multiple-comparisons test .ns, not statistically significant. (a) Tukey's multiple comparison test was performed at a dilution of 2,000.

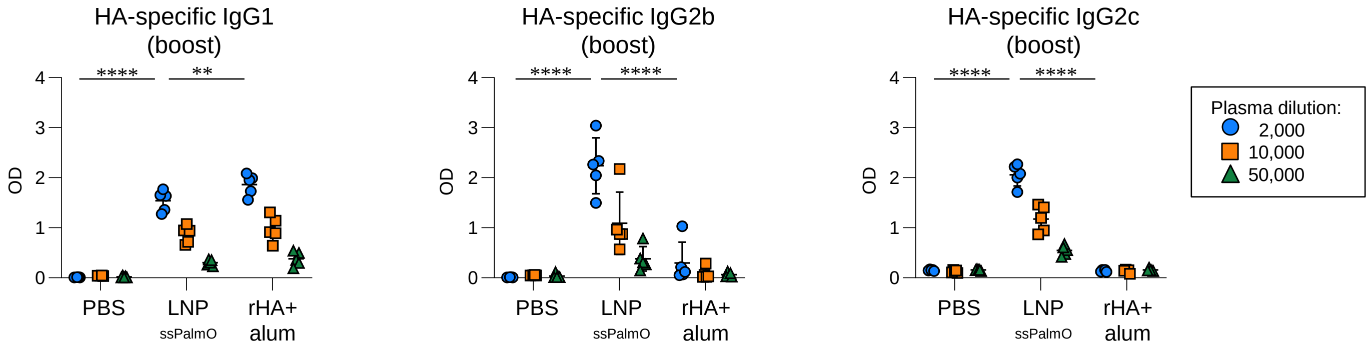

Figure S4. Comparison of antibody responses with rHA plus alum.

Mice were subcutaneously immunized with HA-LNP or rHA plus alum on days 0 (prime) and 21 (boost). Plasma levels of HA-specific IgG1, IgG2b, and IgG2c on day 35 were evaluated using ELISA. n=5 per group. Data are means ± SD. $^{**}P < 0.01$; $^{****}P < 0.0001$, Tukey's multiple-comparisons test. ns, not statistically significant. Tukey's multiple comparison test was performed at a dilution of 2,000.

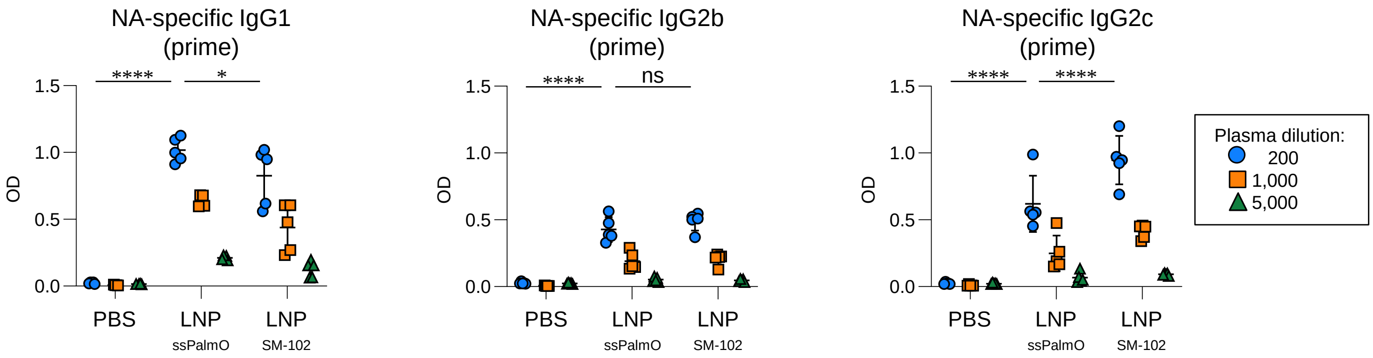

Figure S5. Antibody responses against NA following primary immunization with NA-LNP.
Mice were subcutaneously immunized with NA-LNP on day 0 (prime). The levels of NA-specific IgG1, IgG2b, and IgG2c in plasma on day 14 were measured using ELISA. The data are shown in Figure 2e. n=5 per group. Data are means ± SD. $^{*}P$ < 0.05; $^{****}P$ < 0.0001, Tukey's multiple-comparisons test. ns, not statistically significant. Tukey's multiple-comparison test was performed at a dilution of 200.

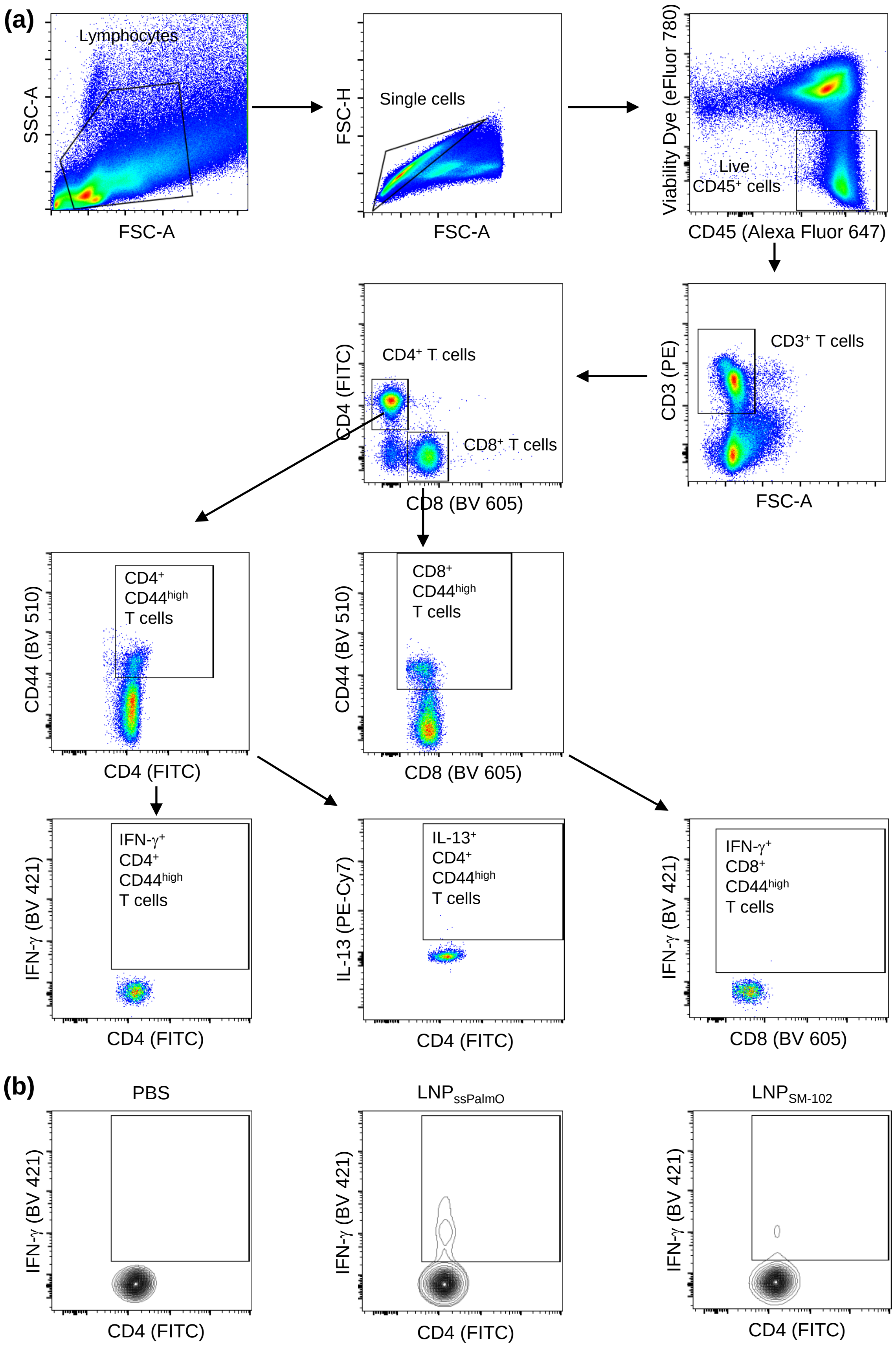

Figure S6. Flow cytometry analysis for cytokine-producing T cells.
(a) Gating strategy and (b) representative plot of IFN-γ$^+$ CD44^{high} CD4$^+$ T cells. The data are related to those in Figure 3.

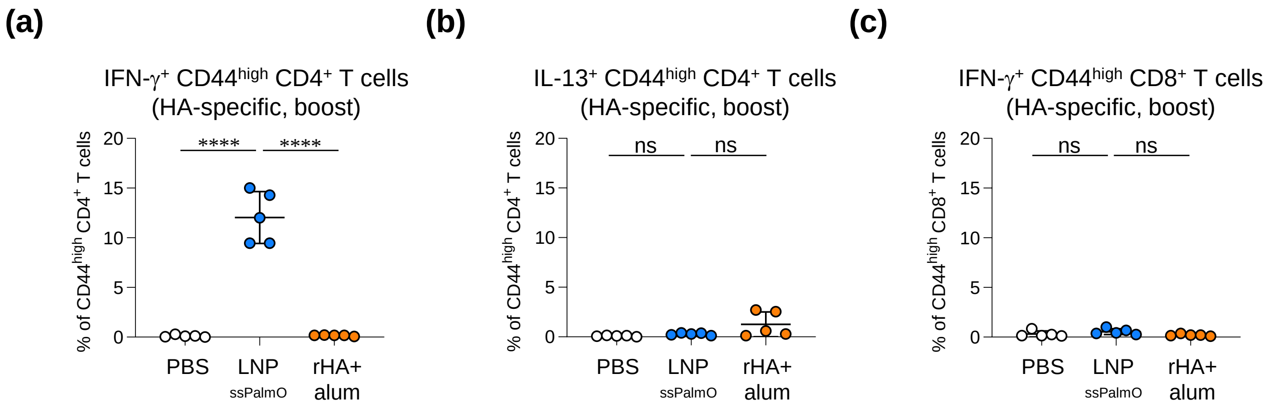

Figure S7. Comparison of T cell responses with rHA plus alum.

Mice were subcutaneously immunized with HA-LNP or rHA plus alum on days 0 (prime) and 21 (boost). On day 35, splenocytes from immunized mice were restimulated with HA. The intracellular cytokine levels in (a) IFN-γ^+ CD44^{high} CD4$^+$ T cells, (b) IL-13$^+$ CD44^{high} CD4$^+$ T cells, and (c) IFN-γ^+ CD44^{high} CD8$^+$ T cells were evaluated. (a-c) n=5 per group. The data represent the means ± SD. $^{****}P < 0.0001$, Tukey's multiple-comparisons test. ns, not statistically significant.

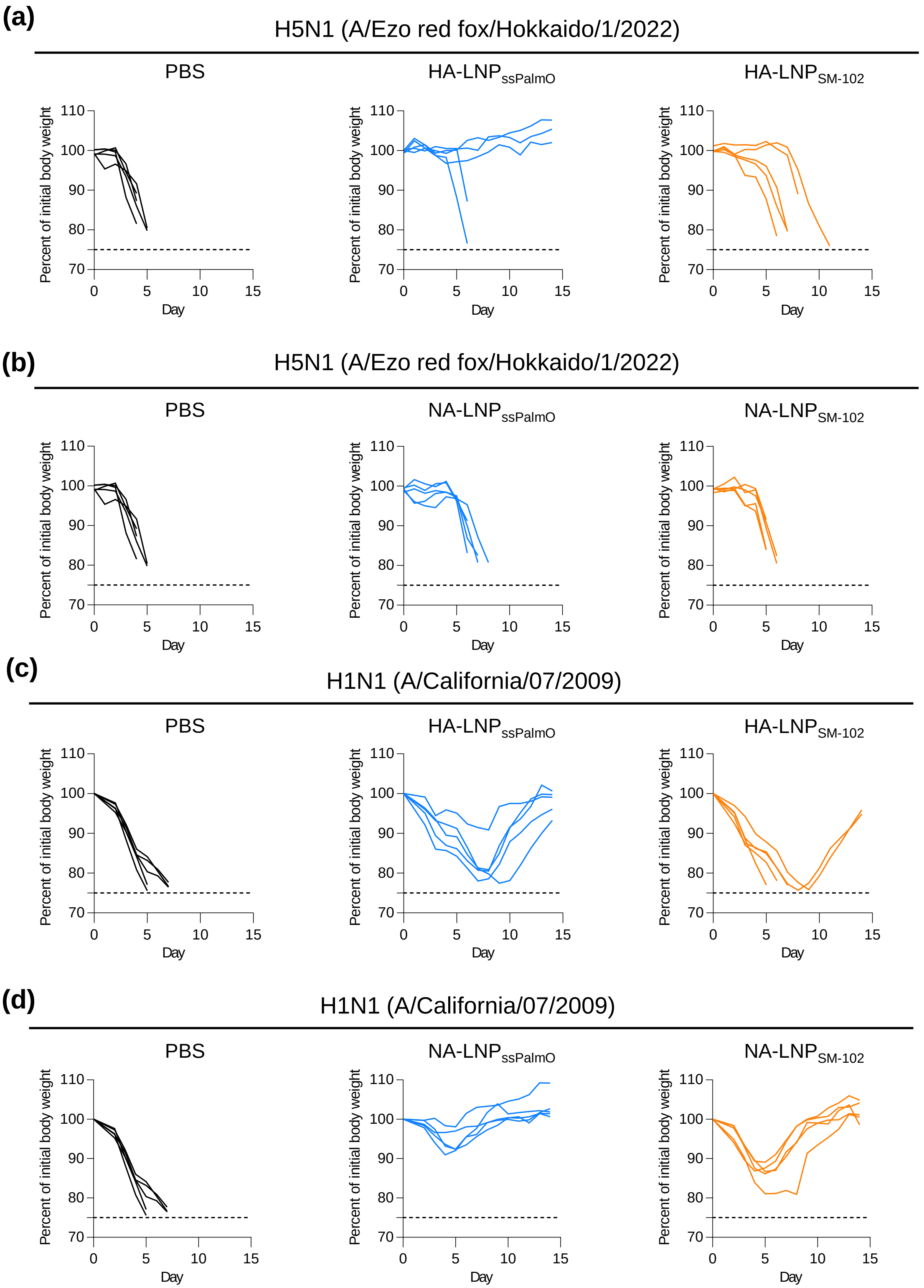

Figure S8. Body weight change in individual mouse after challenge with H5N1 or H1N1 influenza A virus after vaccination with mRNA-LNP.

Mice were subcutaneously immunized with (a, c) HA-LNP or (b, d) NA-LNP on day 0 (prime) and 21 (boost). On the day 35, the mice were intranasally challenged with (a, b) H5N1 A/Ezo red fox/Hokkaido/1/2022 or (c, d) H1N1 A/California/07/2009. Body weight loss in individual mouse was monitored for 14 days after viral challenge. The data are shown in Figure 4. (a-d) n=5 per group.

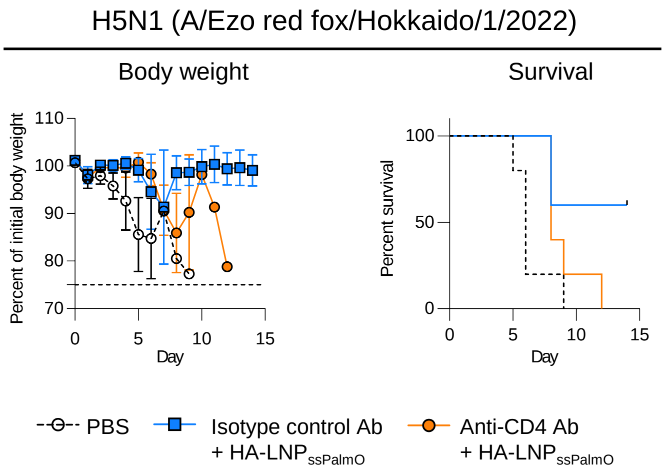

Figure S9. Contribution of CD4$^+$ T cells to cross-protection against heterologous H5N1 influenza virus in mice immunized with HA-LNP$_{ssPalmO}$.

Mice were subcutaneously immunized with HA-LNP$_{ssPalmO}$ on day 0 (prime) and day 21 (boost). On day 35, the mice were intranasally challenged with H5N1 A/Ezo red fox/Hokkaido/1/2022. CD4$^+$ T cells were depleted by anti-CD4 antibody on day 34 and 38. Body weight loss and survival of mice was monitored for 14 days after viral challenge. n=5 per group. Data presented as mean ± SD.

(a)

(b)

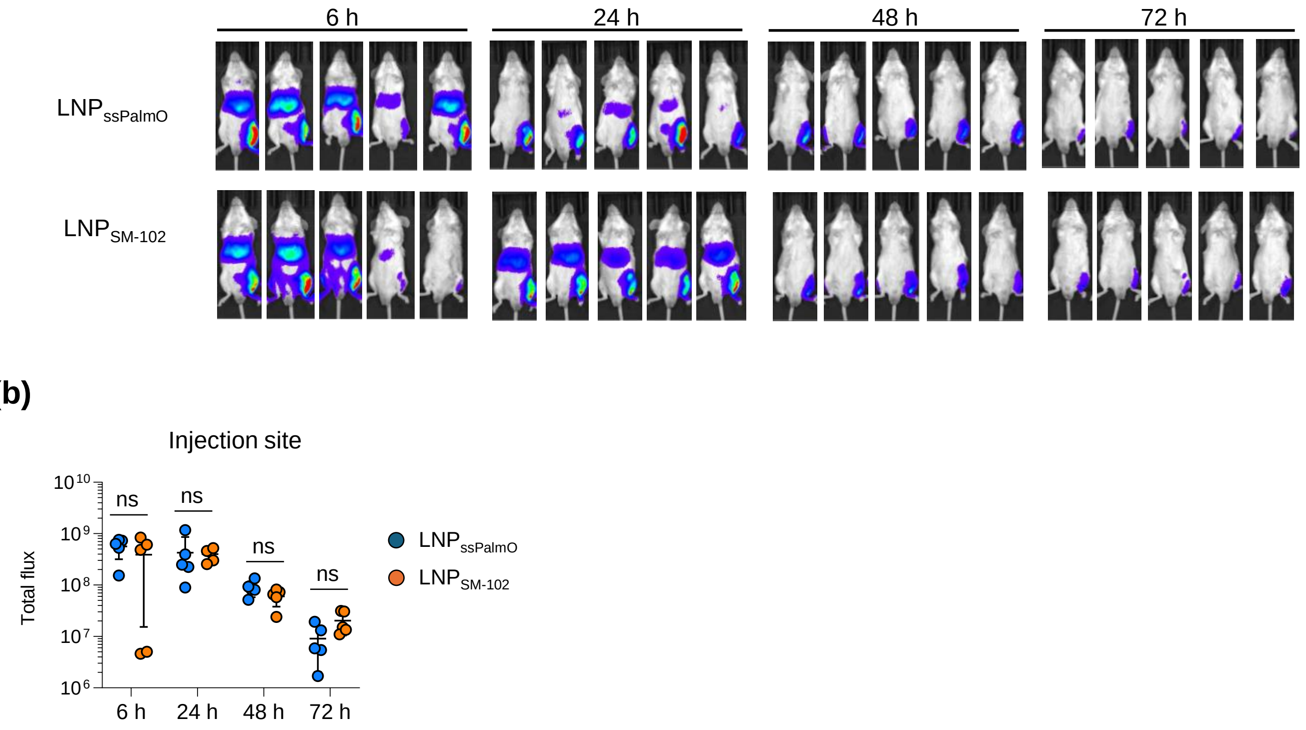

Figure S10. Antigen expression following repeated intramuscular injection with mRNA-LNP. Mice were intramuscularly injected with Luc-LNPs on days 0 (prime) and 21 (boost). At the indicated time points after the booster injection, luminescence was measured using *in vivo* imaging system. (a) Whole images captured on post injection 6, 24, 48, and 72 h. (b) Total flux at the injection site for all images was quantitated. (a-b) n=5 per group. Data are means ± SD. ns, not statistically significant.

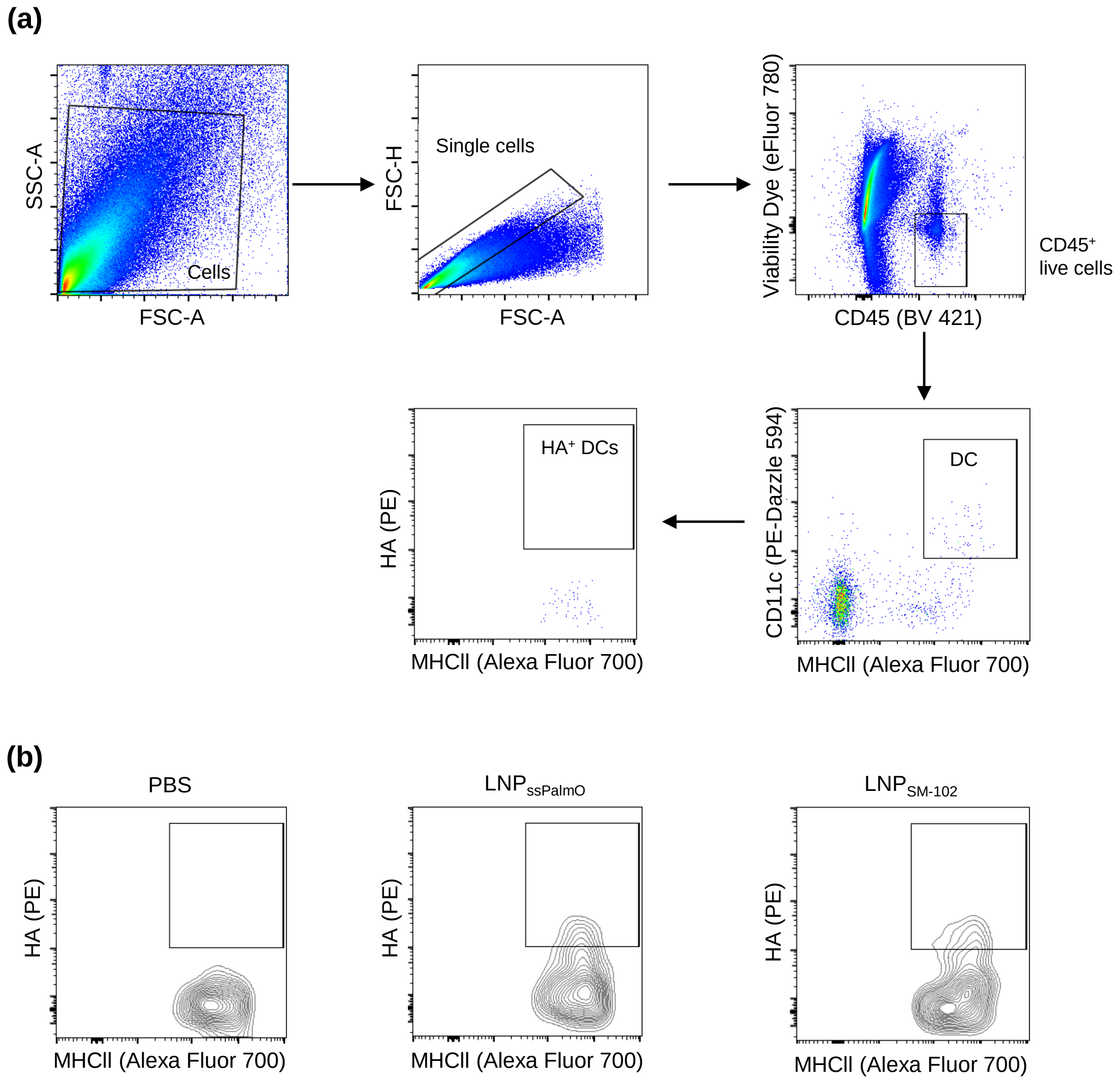

Figure S11. Flow cytometry analysis of HA expression in DCs.
(a) Gating strategy and (b) representative plot of HA⁺ DCs. The data are related to those in Figure 5h.

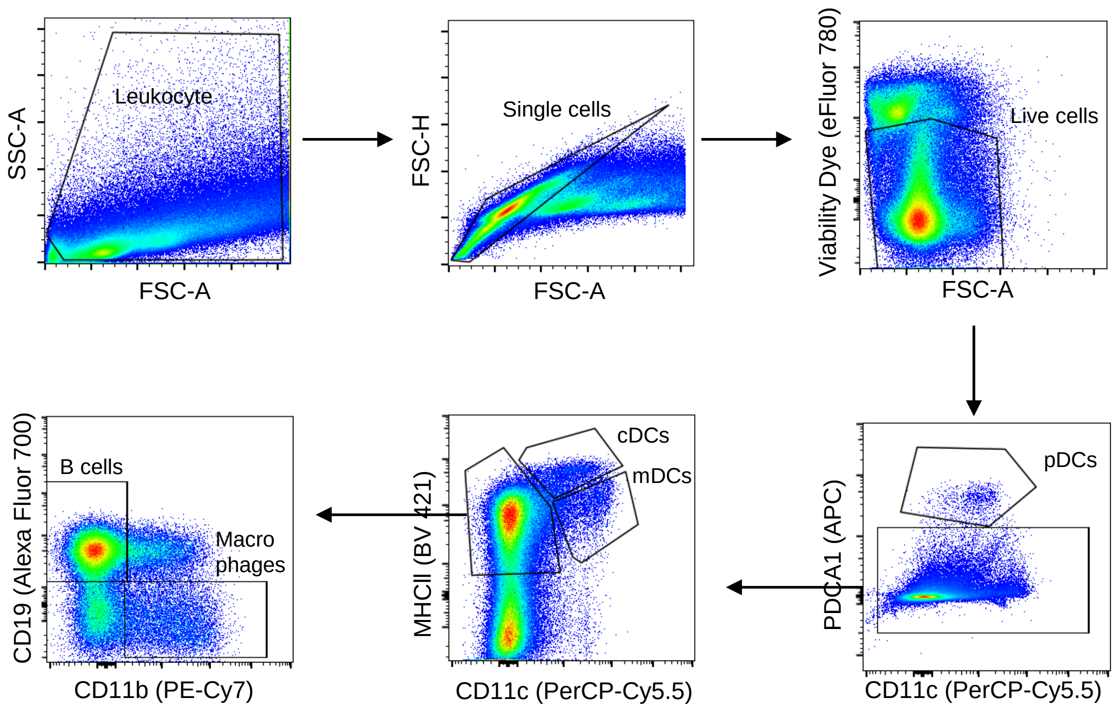

Figure S12. Gating strategy for the flow cytometry analysis of antigen-presenting cell activation. The data are related to those in Figure 6.

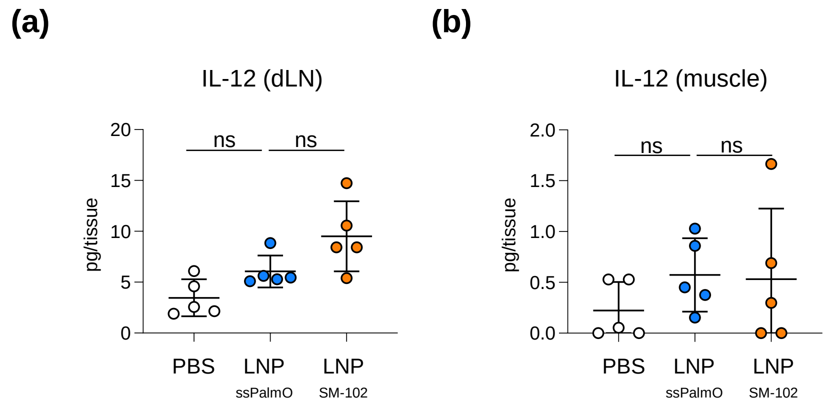

Figure S13. IL-12 levels following immunization with mRNA-LNP.
Mice were (a) subcutaneously or (b) intramuscularly immunized with HA-LNP on day 0. At 6 h after immunization, the level of IL-12 in (a) dLN or (b) muscle were measured. (a, b) n=5 per group. Data are presented as mean ± SD. ns, not statistically significant.

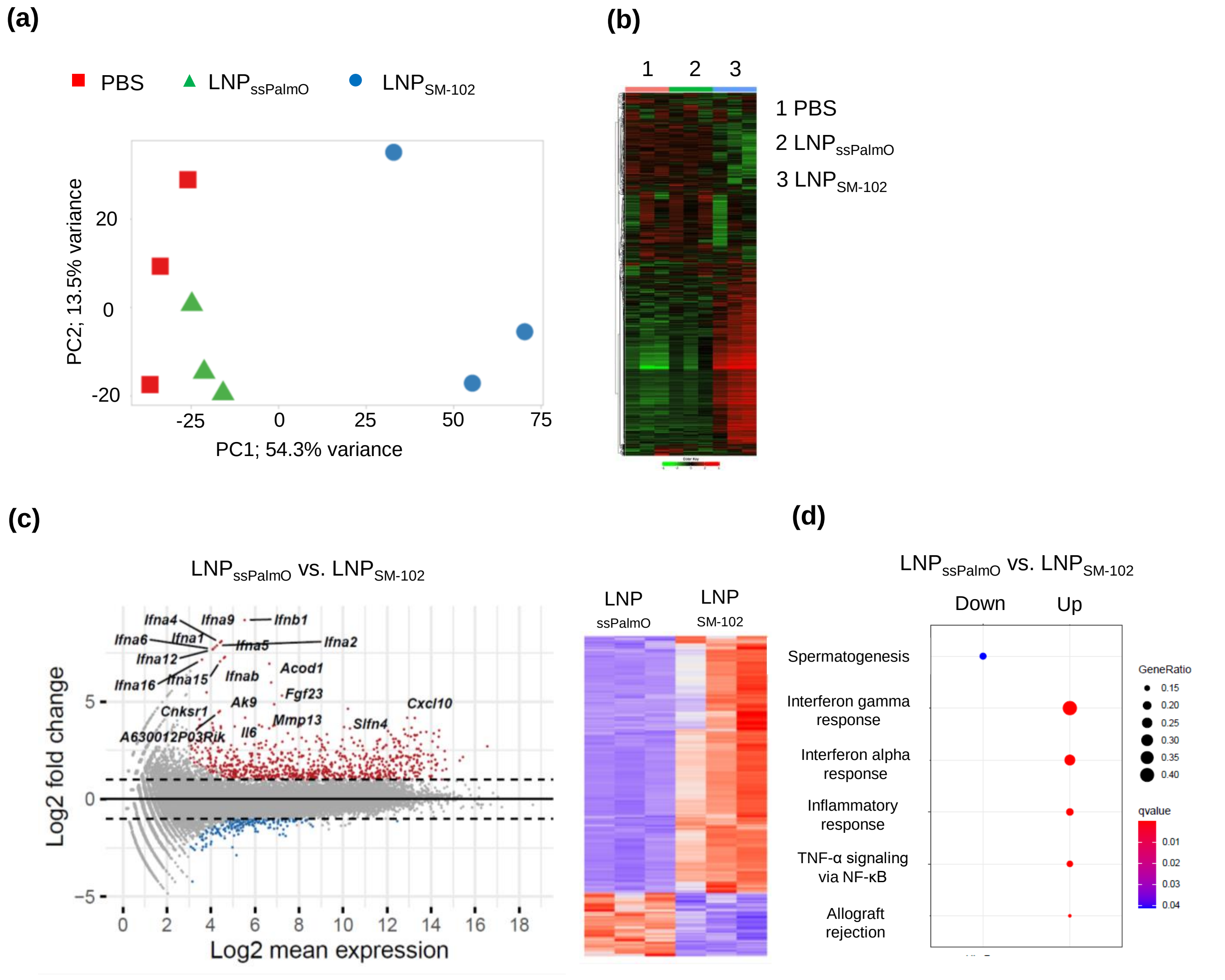

Figure S14. Transcriptional signatures in draining lymph nodes following subcutaneous immunization with mRNA-LNP.
Mice were subcutaneously immunized with HA-LNP and draining lymph nodes were analyzed using RNA sequencing after 6 h. (a) Principal component analysis of differentially expressed genes (DEGs). (b) Heatmap analysis of the expression of the top 2000 DEGs. (c) Mean-average (MA) plot and (d) enrichment analysis of DEGs at 6 h. (a-d) n = 3 per group.

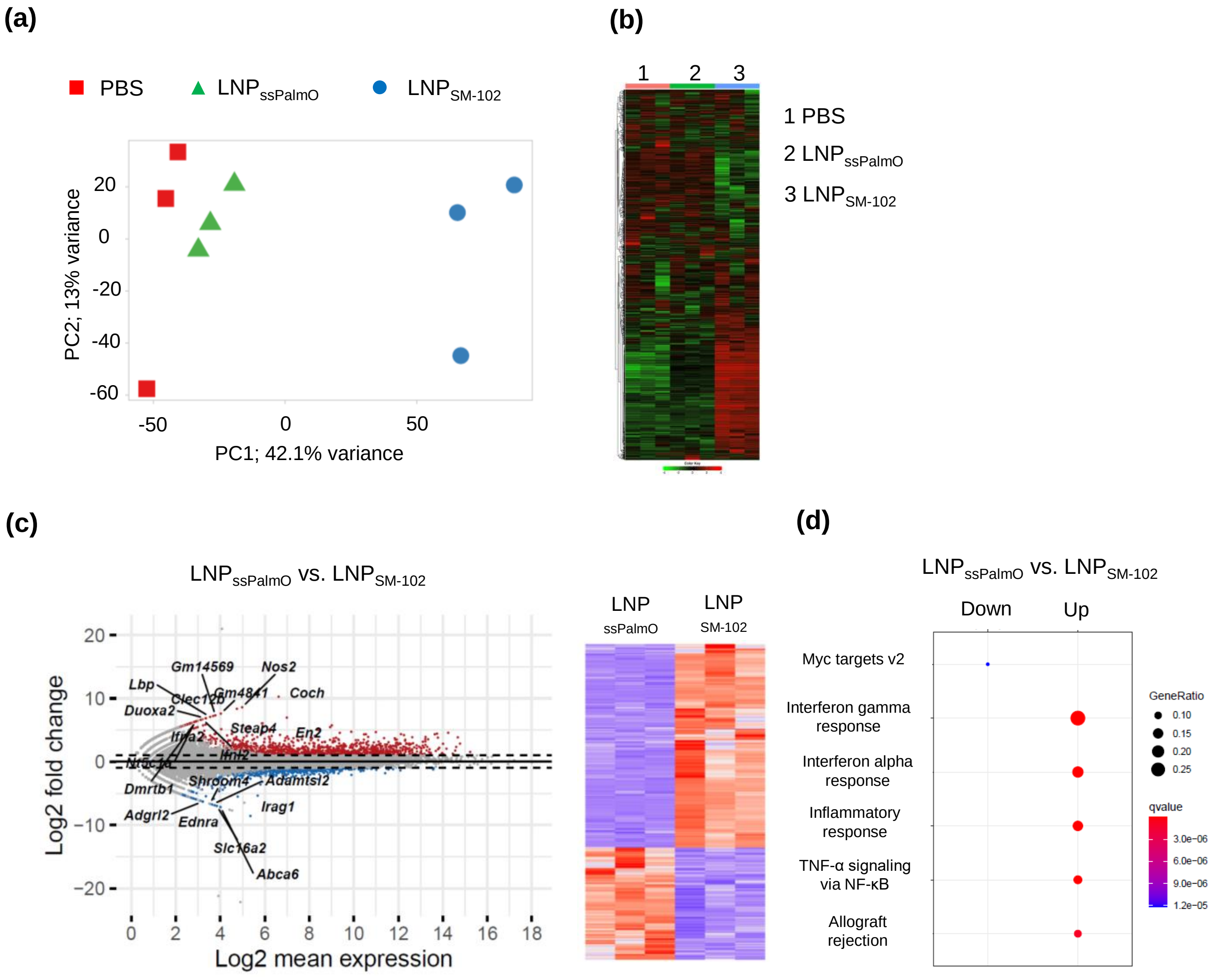

Figure S15. Transcriptional signatures in DCs from draining lymph nodes following subcutaneous immunization with mRNA-LNP.
Mice were subcutaneously immunized with HA-LNP. After 6 h, DCs from draining lymph nodes were analyzed using RNA sequencing. (a) Principal component analysis of DEGs. (b) Heatmap analysis of expression of the top 2000 DEGs. (c) MA plot and (d) enrichment analysis of DEGs at 6 h. (a-d) n = 3 per group.

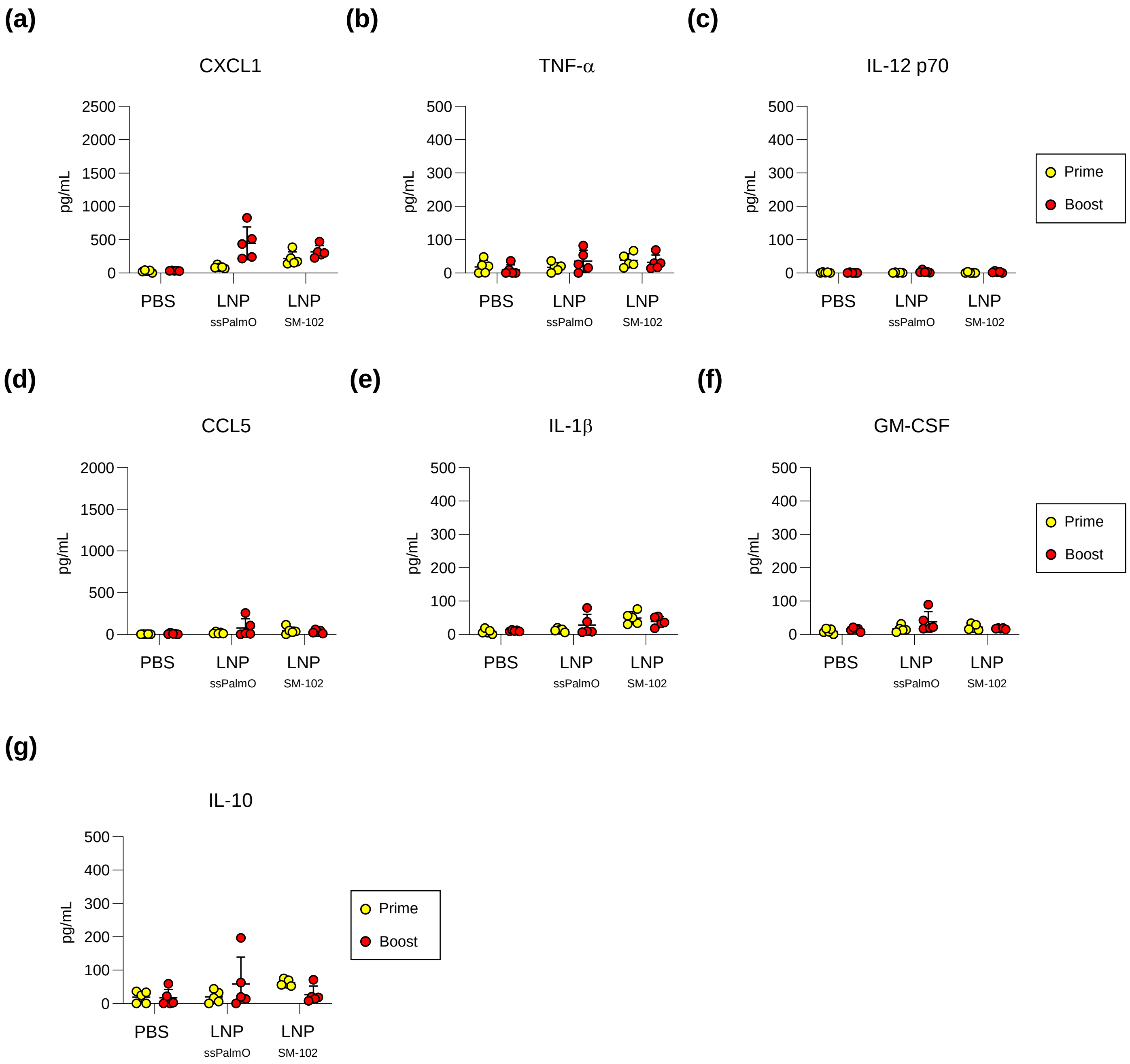

Figure S16. Inflammatory cytokine levels following subcutaneous immunization with mRNA-LNP. Mice were subcutaneously immunized with HA-LNP on days 0 (prime) and 21 (boost). (a-g) At 6 h after primary and booster immunization, the level of (a) CXCL1, (b) TNF-α, (c) IL-12 p70, (d) CCL5, (e) IL-1β, (f) GM-CSF, and (g) IL-10 in plasma were measured. (a-g) n=5 per group. Data are means ± SD.

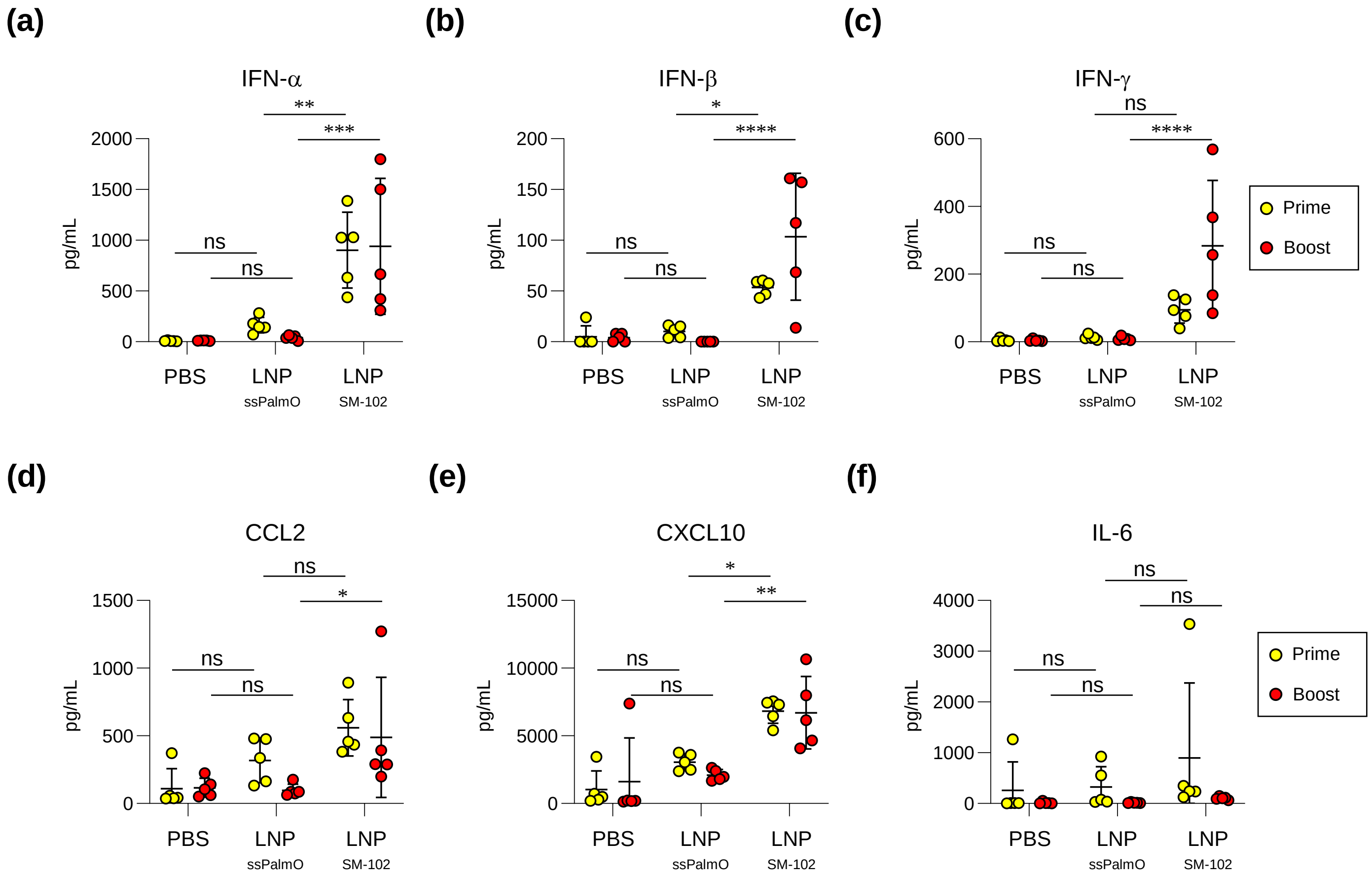

Figure S17. Inflammatory cytokines in the blood following intramuscular immunization with mRNA-LNP.
Mice were immunized intramuscularly with HA-LNP on days 0 (prime) and 21 (boost). At 6 h after primary and booster immunizations, the levels of (a) IFN-α, (b) IFN-β, (c) IFN-γ, (d) CCL2, (e) CXCL10, and (f) IL-6 were measured. (a-f) n=5 per group. Data are means ± SD. $^{*}P < 0.05$; $^{**}P < 0.01$; $^{***}P < 0.001$; $^{****}P < 0.0001$, Tukey's multiple-comparisons test. ns, not statistically significant.

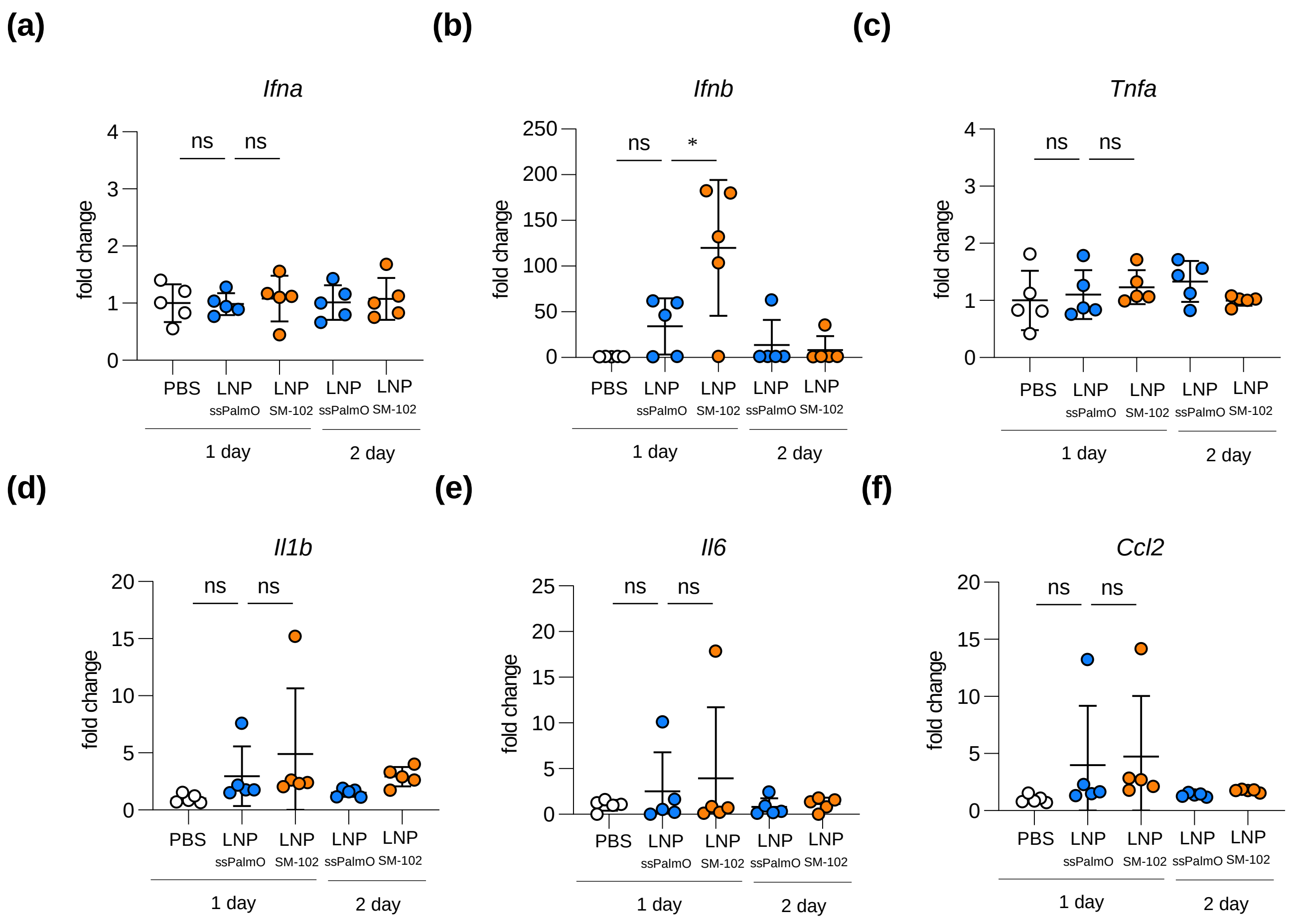

Figure S18. Inflammatory cytokines in heart tissues following intramuscular immunization with mRNA-LNP.
Mice were immunized intramuscularly with spike-LNP on day 0 (prime). At days 1 and 2 after primary immunizations, the mRNA levels of (a) *Ifna*, (b) *Ifnb*, (c) *Tnfa*, (d) *Il1b*, (e) *Il6*, and (f) *Ccl2* were measured. (a-f) n=5 per group. Data are presented as mean ± SD. $^{*}P < 0.05$; Tukey's multiple-comparisons test. ns, not statistically significant.

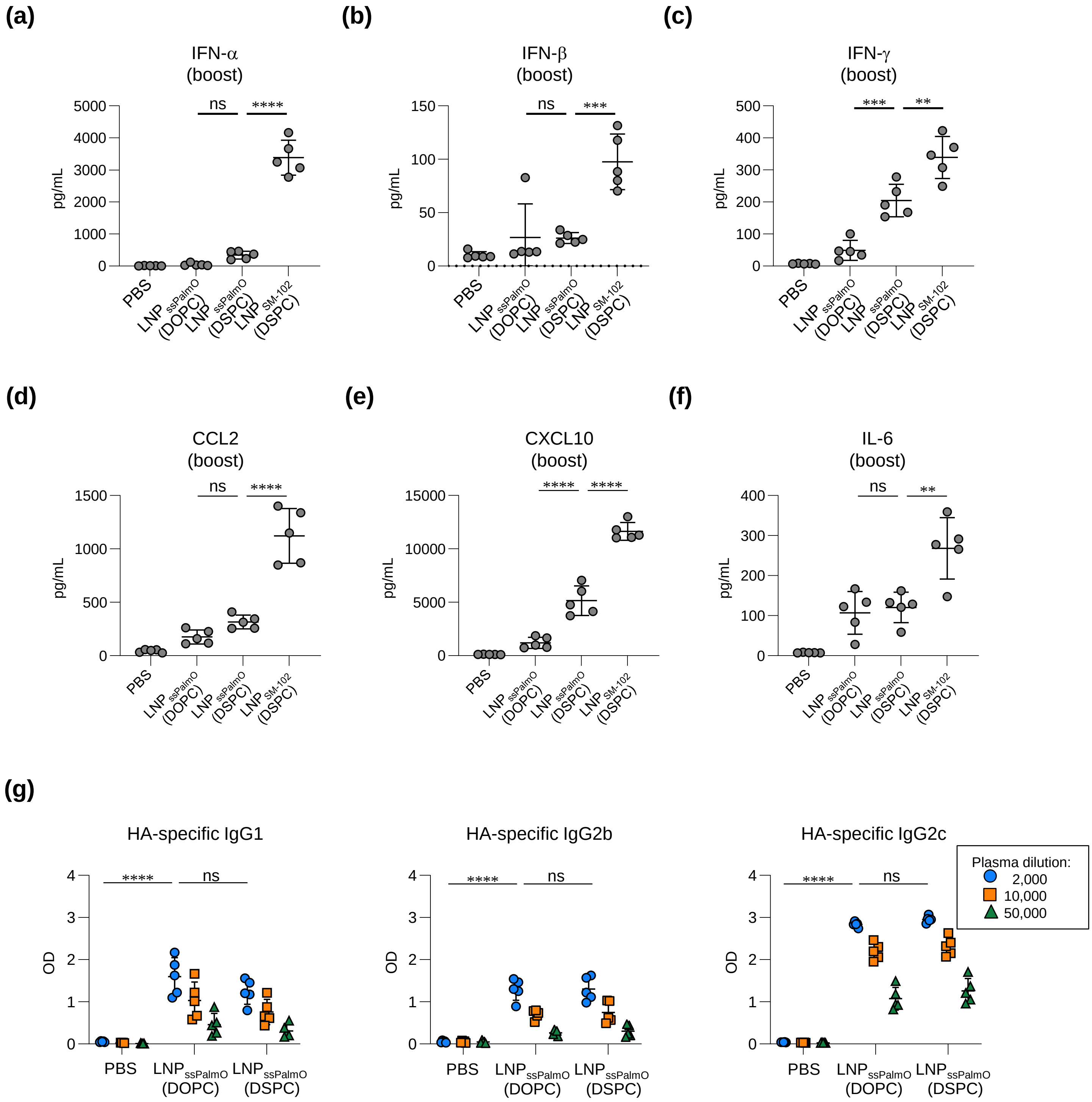

Figure S19. Comparison of inflammatory cytokines and antibody responses between mRNA-LNP$_{ssPalmO}$ with DSPC and mRNA-LNP$_{ssPalmO}$ with DOPC.
Mice were subcutaneously immunized with HA-LNP$_{ssPalmO}$ with DOPC, LNP$_{ssPalmO}$ with DSPC, or LNP$_{SM-102}$ with DSPC on days 0 (prime) and 21 (boost). (a-f) Blood was collected 6 h after boosting. Levels of (a) IFN-α, (b) IFN-β, (c) IFN-γ, (d) CCL2, (e) CXCL10, and (f) IL-6 were measured. (g) Plasma levels of HA-specific IgG1, IgG2b, and IgG2c on day 35 were evaluated using ELISA. (a-g) n=5 per group. Data are means ± SD. $^{**}P < 0.01$; $^{***}P < 0.001$; $^{****}P < 0.0001$, Tukey's multiple-comparisons test. ns, not statistically significant. (g) Tukey's multiple comparison test was performed at a dilution of 2,000.

ATGGAAAAGATCGTGCTGCTGTTCGCCATCGTGTCCCTGGTCAAGAGCGACCAGAT
CTGCATCGGCTACCACGCCAACAACAGCACCGAACAGGTGGACACCATCATGGAAA
AGAATGTGACCGTGACACACGCCCAGGACATCCTCGAGAAGAAGCACAACGGCAA
GCTGTGCGACCTGGACGGCGTGAAGCCTCTGATCCTGAGAGACTGTTCTGTGGCC
GGATGGCTGCTGGGCAACCCTATGTGTGACGAGTTCATCAACGTGCCCGAGTGGTC
CTACATCGTGGAAAAGGCTAACCCCGTGAACGACCTGTGCTACCCCGGCGACTTCA
ACGACTACGAGGAACTGAAGCACCTCCTGAGCAGAATCAACCACTTCGAGAAGATC
CAGATCATCCCCAAGAGCAGCTGGTCCTCTCACGAGGCTTCTCTGGGAGTGTCTAG
CGCATGTCCATACCAGGGCAAGTCCAGCTTCTTCAGAAACGTCGTGTGGCTGATCA
AGAAGAACAGCACATACCCCACCATCAAGCGGAGCTACAACAACACCAATCAAGAG
GACCTGCTGGTGCTGTGGGGCATCCACCATCCTAACGATGCCGCCGAGCAGACCA
AGCTGTACCAGAATCCTACCACCTACATCAGCGTGGGCACCAGCACACTGAACCAG
AGACTGGTGCCTAGAATCGCCACCAGATCCAAAGTGAACGGCCAGAGCGGCAGAAT
GGAATTCTTCTGGACCATCCTGAAGCCTAACGACGCCATCAACTTCGAGAGCAACG
GCAACTTTATCGCCCCTGAGTACGCCTACAAGATCGTGAAGAAGGGCGACAGCACA
ATCATGAAGTCCGAGCTGGAATACGGCAACTGCAACACCAAGTGTCAGACCCCTATG
GGCGCTATCAACTCCAGCATGCCCTTCCACAACATCCATCCTCTGACCATCGGCGA
GTGCCCCAAATACGTGAAGTCCAACAGACTGGTCCTGGCCACCGGCCTGAGAAACT
CTCCACAGCGCGAGCGCAGAAGAAAGAAGAGAGGCCTGTTCGGCGCTATCGCCGG
CTTTATTGAAGGCGGCTGGCAAGGCATGGTGGACGGATGGTACGGATACCACCACA
GCAACGAGCAAGGCTCTGGCTACGCCGCCGACAAAGAGAGCACACAGAAAGCCAT
CGACGGCGTCACCAACAAAGTGAATAGCATCATCGACAAGATGAACACCCAGTTCG
AGGCCGTGGGCAGAGAGTTCAACAACCTGGAAAGAAGGATCGAGAACCTGAACAA
GAAAATGGAAGATGGCTTCCTGGACGTGTGGACCTACAACGCTGAGCTGCTCGTGC
TGATGGAAAACGAGAGAACCCTGGACTTCCACGACTCCAACGTGAAGAACCTGTAC
GACAAAGTGCGGCTGCAGCTGAGAGACAACGCCAAAGAACTCGGCAACGGCTGCT
TCGAGTTCTACCACAAGTGCGACAACGAGTGCATGGAAAGCGTGCGGAACGGCAC
CTACGACTACCCTCAGTACAGCGAGGAAGCCAGACTGAAGAGGGAAGAGATCAGC
GGAGTGAAGCTGGAATCCATCGGCATCTACCAGATCCTGAGCATCTACAGCACCGT
GGCCTCTTCTCTGGCCCTGGCTATTATGGTGGCTGGCCTGAGCCTGTGGATGTGCT
CTAACGGCAGCCTGCAGTGCAGAATCTGCATCTGA

Figure S20. Sequence for H5N1 HA.

ATGAACCCCAACCAGAAGATCATCACCATCGGCAGCATCTGCATGGTCACCGGCATC
GTGTCTCTGATGCTGCAGATCGGCAACATGATCAGCATCTGGGTGTCCCACAGCATC
CACACCGGAAACCAGCACCAGAGCGAGCCTATCAGCAACACCAACTTTCTGACCGA
GAAGGCCGTGGCCTCTGTGAAGCTGGCTGGCAACTCTAGCCTGTGTCCTATCAACG
GCTGGGCCGTGTACAGCAAGGACAACAGCATCAGGATCGGCTCCAAGGGCGACGT
GTTCGTGATCAGAGAGCCCTTCATCAGCTGCAGCCACCTGGAATGCAGAACATTCTT
CCTGACACAAGGCGCCCTGCTGAACGACAAGCACAGCAACGGCACCGTGAAGGAC
AGAAGCCCTCACAGAACCCTGATGAGCTGCCCTGTGGGCGAAGCCCCATCTCCTTA
CAACAGCAGATTCGAGTCCGTGGCTTGGAGCGCCTCTGCTTGTCACGATGGCACCA
GCTGGCTGACAATCGGCATCAGCGGACCTGATAACGGCGCTGTGGCTGTGCTGAA
GTACAACGGCATCATCACCGACACCATCAAGAGCTGGCGGAACAACATCCTGAGAA
CCCAAGAGTCCGAGTGCGCCTGTGTGAACGGCAGCTGTTTCACCGTGATGACAGA
CGGCCCTTCTAACGGCCAGGCCAGCCACAAGATCTTCAAGATGGAAAAGGGCAAAG
TGGTCAAGAGCGTGGAACTGGACGCCCCTAACTACCACTACGAGGAATGCAGCTGC
TACCCCAACGCCGGCGAGATCACCTGTGTGTGCAGAGATAACTGGCACGGCAGCAA
CAGACCTTGGGTGTCCTTCAACCAGAACCTGGAATACCAGATCGGCTATATCTGCAG
CGGCGTGTTCGGCGACAACCCCAGACCTAATGATGGCACAGGCAGCTGCGGACCC
GTGTCTAGCAATGGTGCTTACGGCGTGAAGGGCTTCAGCTTTAAGTACGGCAACGG
CGTGTGGATCGGCAGGACCAAGAGCACCAACTCCAGATCCGGCTTCGAGATGATCT
GGGACCCTAACGGCTGGACCGAGACAGACAGCAGCTTCAGCGTGAAGCAGGACAT
CGTGGCCATCACCGATTGGAGCGGCTACAGCGGCTCTTTCGTGCAGCACCCTGAAC
TGACAGGCCTGGACTGCATCAGACCCTGTTTCTGGGTCGAGCTGATCAGAGGCAGA
CCCAAAGAGAGCACCATCTGGACCAGCGGCAGCAGCATCTCTTTCTGCGGCGTGAA
CAGCGACACCGTCGGATGGTCTTGGCCTGATGGTGCTGAGCTGCCTTTCACCATCG
ACAAGTGA

Figure S21. Sequence for H5N1 NA.

Table S1. Antibodies used for ELISA and flow cytometry analysis.

ELISA

	Clone	Catalog number	Dilution	Source
HRP anti-mouse IgG1		1070-05	1:8000	SouthernBiotech
HRP anti-mouse IgG2b		1090-05	1:5000	SouthernBiotech
HRP anti-mouse IgG2c		1078-05	1:8000	SouthernBiotech

Germinal center B cell analysis

	Clone	Catalog number	Dilution	Source
Anti-mouse CD16/CD32	93	101302	1:200	BioLegend
Fixable Viability Dye eFluor 780		65-0865-18	1:1000	Thermo Fisher Scientific
Alexa Fluor 647 anti-mouse/human GL7	GL7	144606	1:200	BioLegend
Alexa Fluor 700 anti-mouse CD19	6D5	115528	1:200	BioLegend
PE/Cy7 anti-mouse CD95	SA367H8	152618	1:200	BioLegend

Intracellular cytokine in T cells

	Clone	Catalog number	Dilution	Source
Anti-mouse CD16/CD32	93	101302	1:200	BioLegend
Fixable Viability Dye eFluor 780		65-0865-18	1:1000	Thermo Fisher Scientific
Alexa Fluor 647 anti-mouse CD45	30-F11	103124	1:200	BioLegend
PE anti-mouse CD3	145-2C11	100308	1:200	BioLegend
FITC anti-mouse CD4	GK1.5	100406	1:200	BioLegend
BV 605 anti-mouse CD8a	53-6.7	100744	1:200	BioLegend
BV 510 anti-mouse CD44	IM7	103044	1:200	BioLegend
BV 421 anti-IFN-γ	XMG1.2	505830	1:200	BioLegend
PE/Cy7 anti-IL-13	eBio13A	25-7133-82	1:200	Thermo Fisher Scientific

HA expression

	Clone	Catalog number	Dilution	Source
Anti-mouse CD16/CD32 antibody	93	101302	1:200	BioLegend
Fixable Viability Dye eFluor 780		65-0865-18	1:1000	Thermo Fisher Scientific
BV421 anti-mouse CD45	30-F11	103134	1:200	BioLegend
Alexa Fluor 700 anti-mouse I-A/I-E	M5/114.15.2	107622	1:200	BioLegend
PE-Dazzle 594 anti-mouse CD11c	N418	117348	1:200	BioLegend
anti-HA		11062-T62	1:1000	Sino Biological
PE anti-rabbit IgG	Poly4064	406421	1:1000	BioLegend

Activation of antigen presenting cells

	Clone	Catalog number	Dilution	Source
Anti-mouse CD16/CD32 antibody	93	101302	1:200	BioLegend
Fixable Viability Dye eFluor 780		65-0865-18	1:1000	Thermo Fisher Scientific
PerCP/Cy5.5 anti-mouse CD11c	N418	117328	1:200	BioLegend
APC anti-mouse PDCA1	927	127016	1:200	BioLegend
Alexa Fluor 700 anti-mouse CD19	6D5	115528	1:200	BioLegend
BV421 anti-mouse I-A/I-E	M5/114.15.2	107632	1:200	BioLegend
PE anti-mouse CD86	GL-1	105008	1:200	BioLegend
PE/Cy7 anti-mouse CD11b	M1/70	101216	1:200	BioLegend

Isolation of DCs

	Clone	Catalog number	Dilution	Source
Fixable Viability Dye eFluor 780		65-0865-18	1:1000	Thermo Fisher Scientific
Alexa Fluor 488 anti-CD90.2	30-H12	105316	1:500	BioLegend
Alexa Fluor 700 anti-mouse I-A/I-E	M5/114.15.2	107622	1:200	BioLegend
PE-Dazzle 594 anti-mouse CD11c	N418	117348	1:200	BioLegend
PE-Cy7 anti-mouse CD19	6D5	115520	1:200	BioLegend
BV421 anti-mouse CD45	30-F11	103134	1:200	BioLegend

Table S2. Primers for RT-PCR.

	Forward	Reverse
Ifna	5′-GGACTTTGGATTCCCGCAGGAGAAG-3′	5′-GCTGCATCAGACAGCCTTGCAGGTC-3′
Ifnb	5′-AACCTCACCTACAGGGCGGACTTCA-3′	5′-TCCCACGTCAATCTTTCCTCTTGCTTT-3′
Tnfa	5′-CCTCTCATGCACCACCATCAA-3′	5′-TTCTGAGACAGAGGCAACCTG-3′
Il1b	5′-TCTTTGAAGTTGACGGACCC -3′	5′-TGAGTGATACTGCCTGCCTG-3′
Il6	5′-CTGTAGCTCATTCTGCTCTGGA-3′	5′-CAACTGGATGGAAGTCTCTTGC-3′
Ccl2	5′-CACTCACCTGCTGCTACTCA-3′	5′-TCTTGAGCTTGGTGACAAAAACTAC-3′
Gapdh	5′-CAGGTTGTCTCCTGCGACTT-3′	5′-AGCCGTATTCATTGTCATACCAGG-3′
